# A comprehensive map of single-base polymorphisms in the hypervariable *LPA* kringle IV type 2 copy number variation region[S]

**DOI:** 10.1194/jlr.M090381

**Published:** 2018-11-09

**Authors:** Stefan Coassin, Sebastian Schönherr, Hansi Weissensteiner, Gertraud Erhart, Lukas Forer, Jamie Lee Losso, Claudia Lamina, Margot Haun, Gerd Utermann, Bernhard Paulweber, Günther Specht, Florian Kronenberg

**Affiliations:** Division of Genetic Epidemiology* Department of Medical Genetics, Molecular and Clinical Pharmacology, Medical University of Innsbruck, Innsbruck, Austria; Division of Human Genetics,† Department of Medical Genetics, Molecular and Clinical Pharmacology, Medical University of Innsbruck, Innsbruck, Austria; First Department of Internal Medicine,§ Paracelsus Private Medical University, Salzburg, Austria; Department of Database and Information Systems,** Institute of Computer Science, University of Innsbruck, Innsbruck, Austria

**Keywords:** genetics, genomics, apolipoproteins, apolipoprotein (a), lipoproteins, lipoprotein (a), molecular biology, copy number variation

## Abstract

Lipoprotein (a) [Lp(a)] concentrations are among the strongest genetic risk factors for cardiovascular disease and present pronounced interethnic and interindividual differences. Approximately 90% of Lp(a) variance is controlled by the *LPA* gene, which contains a 5.6-kb-large copy number variation [kringle IV type 2 (KIV-2) repeat] that generates >40 protein isoforms. Variants within the KIV-2 region are not called in common sequencing projects, leaving up to 70% of the *LPA* coding region currently unaddressed. To completely assess the variability in *LPA*, we developed a sequencing strategy for this region and report here the first map of genetic variation in the KIV-2 region, a comprehensively evaluated ultradeep sequencing protocol, and an easy-to-use variant analysis pipeline. We sequenced 123 Central-European individuals and reanalyzed public data of 2,504 individuals from 26 populations. We found 14 different loss-of-function and splice-site mutations, as well as >100, partially even common, missense variants. Some coding variants were frequent in one population but absent in others. This provides novel candidates to explain the large ethnic and individual differences in Lp(a) concentrations. Importantly, our approach and pipeline are also applicable to other similar copy number variable regions, allowing access to regions that are not captured by common genome sequencing.

Lipoprotein(a) [Lp(a)] is one of the strongest genetic risk factors for CVD. Approximately 25% of the Caucasian population present concentrations that increase their risk for CVD by >2- to 3-fold (1, 2). Lp(a) presents extraordinary large inter- and intrapopulation variation (3, 4). In Caucasians, Lp(a) concentrations are highly right-skewed and show an ∼1,000-fold interindividual range (0.3–300 mg/dl; median: 10–12 mg/dl) (1), and even within Europe concentrations vary by 2- to 3-fold (4–7). Conversely, in most African populations, median Lp(a) concentrations are ∼4–5 times higher, and the distribution is nearly Gaussian (3). Asian populations show intermediate values (4–7).

The *LPA* gene locus explains >90% of the Lp(a) variance (1). It consists of 10 homologous kringle IV (KIV) domains (types 1–10) followed by 1 kringle V domain (1) and a protease domain. KIV-2 is encoded by a 5.6 kb-sized copy number variation that contains 2 exons (sized 160 and 182 bp) spaced by a 4 kb-sized intron and can be present in 1 to >40 copies per allele (1, 8, 9). The combination of three silent changes at positions 14 (A>G), 41 (T>C), and 86 (A>T) of the first KIV-2 exon (10) defines three KIV-2 subtypes (i.e., haplotypes) (10, 11) that have been reported to occur at different relative percentages among ethnicities (12). The KIV-2 repeat number explains 30% to 70% (1) of Lp(a) variance in an inverse, nonlinear manner (4). In Europeans, low molecular weight isoforms (≤22 total KIV repeats) are associated with 4- to 5-fold higher Lp(a) concentrations (median: ∼45 mg/dl) than high molecular weight isoforms (>22 KIV repeats; median: 8–10 mg/dl) (13). Intriguingly, the same isoform size is associated with a more than 100-fold range in Lp(a) concentrations in the general population (14) but with only a 2.5-fold range within families (14, 15). This suggests the existence of additional genetic elements in *LPA* that independently modify Lp(a) concentrations.

However, only few bona fide functional and/or loss-of-function variants in *LPA* have been identified so far. A frequent splice-site mutation in KIV-8 has been shown to account for approximately 25% of all null alleles in Caucasians (16), and three additional, rarer splice-site variants have been recently described (12, 17, 18). Mutation screenings in *LPA* are hampered by the fact that KIV-2 encompasses up to 70% of the coding sequence. Due to the ambiguous mapping of sequencing reads in this region, variants in KIV-2 are not covered by current reference data sets such as the 1000 Genomes Project (19), the Exome Aggregation Consortium (20), the Genome Aggregation Database (20), or the Trans-Omics for Precision Medicine program or by recent large-scale sequencing efforts (17). Indeed, few mutation screenings in the *LPA* KIV-2 have been performed until now (12, 21, 22), although the importance of the region has been reinforced by the identification of a relatively common nonsense mutation in KIV-2 [variant p.(Arg21Ter); minor allele frequency = 1.6% (11)] and, more recently, by the identification of a frequent variant (variant 4925G>A) that lowers Lp(a) by up to 70% and indeed explains 19% of the Lp(a) variance in the low molecular weight carriers (23). Given the large contribution of the KIV-2 region to the total coding sequence of *LPA*, it might contain further novel genetic variants that provide new insights in Lp(a) genetics. For example, some restriction patterns of the so-called DraIII restriction polymorphism are associated with very specific Lp(a) concentrations (24), but the underlying base change has not been identified.

Early studies either applied laborious cloning approaches (11) or leveraged the high homology in KIV-2 to amplify all repeats simultaneously as an amplicon mixture by using primers that bind in every repeat (12, 22). This amplicon mixture was then subjected to Sanger sequencing (a concept known as batch sequencing; see supplemental Fig. S1A–C for an explanation of this concept and its implication for data analysis) (22). Any mutation present only in a subset of kringles will be present only in a subset of amplicons and therefore detectable as a low-level mutation underlying the principal sequencing signal [similar to mitochondrial DNA heteroplasmies (25) or somatic mutations (26)]. However, the detection of 1 mutant base in up to 80 repeats (1.2% mutant fraction) was far beyond the sensitivity of Sanger sequencing (27), and accordingly early studies observed only few variants (22). Modern next-generation sequencing (NGS) technologies provide unprecedented sensitivity in detecting variants present in down to 1% of all amplicons (26, 27) (we refer to the detected fractional representation of a mutant sequencing read among all reads herein as “mutation level” or “variant level”), if backed by sophisticated bioinformatics (25). We have established an ultradeep sequencing method for the KIV-2 region and *1*) present here a technical evaluation of the sequencing protocol; *2*) make our *LPA* KIV-2 sequencing analysis pipeline freely available to the community; *3*) report observed pitfalls in KIV-2 sequencing; *4*) provide a comprehensive overview for all KIV-2 variants observed in an extended analysis of our recent batch-sequencing data set (23) and link them to previous, less standardized studies; and *5*) report the variants observed in the 26 worldwide populations from 1000 Genomes.

## METHODS

### Batch amplification of KIV-2

Sequencing was done following a batch-sequencing approach (23, 28) using the KIV-2 batch amplification primer published by Noureen et al. (12). Primer set 1 (primers 421U_2 and 422L; amplicon PCR5104) is located in the small interkringle intron and spans the long intrakringle intron (see supplemental Fig. S2 for an overview). Primer set 2 (primers 422U and 421L; amplicon PCR2645) is located in the long intrakringle intron and spans the interkringle intron (supplemental Fig. S2). PCR conditions are given in supplemental Table S1. Primers are given in supplemental Table S2.

### NGS protocol

Sequencing libraries (plasmids and population sample genomic DNA) for NGS were prepared as described previously (23) following the Illumina TruSeq Nano library protocol. Sequencing was performed on an Illumina MiSeq system using reagent kit v2 with 500 cycles. A detailed step-by-step pipetting protocol is provided at https://github.com/genepi/lpa-pipeline. Technical details can be found in supplemental Methods.

### Reference sequence and definition of KIV-2B-specific variants

The haplotype of three silent variants in the first KIV-2 exon (supplemental Fig. S3) defines three KIV-2 subtypes (KIV-2A, KIV-2B, and KIV-2C) (10, 11). We reference all data of the PCR5104 amplicon to the sixth repeat in hg19 (chr6:161,033,785–161,038,888), while the PCR2645 reference is the preceding interkringle intron (chr6:161,037,674–161,040,318). Because the *LPA* RefSeq sequence contains six KIV-2 repeats, variants cannot be uniquely mapped to a position when using RefSeq as a reference as would be required by Human Genome Variation Society nomenclature (29). We therefore reference all DNA positons in this article to the two KIV-2 sequences specified above. See supplemental Methods for a more in-depth definition and discussion of the reference sequence definition. Moreover, because exon numbering is not unique in the copy number repeat, we name the two exons in KIV-2 using the convenient nomenclature proposed by Noureen et al. (12): exon 421 stands for KIV-2, exon 1, and exon 422 stands for KIV-2, exon 2.

KIV-2B presents also several intronic differences (supplemental Fig. S4, supplemental Alignment). In this article we thus use the wording “KIV-2B variant” for all differences to the reference (criteria specified in detail in supplemental Methods), while the three exonic KIV-2B variants described by McLean et al. (10) and by Parson et al. (11) are referred to as “canonical KIV-2B variants.” Importantly, all KIV-2B variants represent one molecular haplotype being present in a linked manner on each KIV-2B amplicon molecule. Therefore, all KIV-2B variants shall always be present at the same mutation level.

### Cloning of KIV-2A and KIV-2B

A KIV-2A and KIV-2B amplicon were isolated by PCR subcloning and used to generate defined clone mixes. Because the KIV-2A and the KIV-2B haplotypes differ by several positions, PCR amplification on these mixes generates amplicon mixes with dozens of differences distributed over the whole amplicon. These differences were used to generate calibration data for the bioinformatic analysis pipeline. Technical details are given in supplemental Methods.

### NGS validation experiment

In order to calibrate and benchmark our bioinformatic analysis pipeline, the KIV-2A and KIV-2B plasmids were accurately quantified using a Qubit 3.0 fluorometer with the Qubit High-Sensitivity Kit (Thermo Fisher Scientific) and mixed at the following ratios: 50:50, 90:10, 95:5, 97.5:2.5, 98.5:1.5, and 99:1 (corresponding to a fractional representation of the KIV-2B plasmid of 50%, 10%, 5%, 2.5%, 1.5%, and 1%, respectively). The type B plasmid always represented the minor component to mimic the in vivo situation, where KIV-2B repeats are rarer than KIV-2A repeats (12). The mixtures were then used as a template for PCR amplification using three different polymerases: Herculase II Fusion (Agilent Technologies), LongAmp Taq DNA Polymerase (LA; NEB), and the LongRange PCR Kit (LR; QIAGEN) (supplemental Table S1). These products were used as templates for Illumina library preparation (see below). To assess reproducibility, all Herculase libraries were prepared three times, including the PCR amplification, and subjected to ultradeep MiSeq sequencing (complete process replicates). Additionally, in a second experiment, the 50% mixes for all three polymerases were again prepared in duplicates, including the PCR, as well as the 1% mixes of Herculase II and LR.

### Populations for de novo amplicon sequencing (“discovery set”)

A total of 123 samples from two Central-European populations were used to generate KIV-2 variability data using a targeted amplicon-sequencing approach [batch-sequencing approach (22, 23)]. The methods used for Lp(a) quantification [ELISA in plasma (4, 30)] and isoform size quantification [agarose-based Western blot (30)] have been described previously (23, 31, 32) and are summarized in supplemental Methods. The studies complied with the Declaration of Helsinki and were accepted by the respective institutional review boards.

### Data analysis (*LPA* pipeline)

When mapping all KIV-2 reads to only one reference repeat, mutations present in a fraction of kringles show behavior to mitochondrial DNA heteroplasmies, where mutations are only present in a few copies of the mitochondrial genome. Because mutations are only present in a subset of kringles, each position in the pileup file can consist of a mixture of different bases. The *LPA* pipeline consists of several computational steps resulting in an annotated variant file. Input samples are accepted in FASTQ format and mapped to the sixth reference repeat (KIV-2A) as described above. Predefined filter values for base quality (Q20), mapping (Q20), and alignment quality (Q30) are applied, and a pileup format is compiled from the intermediate BAM file by estimating base occurrences for each position strand independently. The pileup format is used to call variants down to 1% (referred to as “low-level variants” or “low-level mutations”) by using a method originally developed for detecting mitochondrial DNA heteroplasmy (25). The method integrates a maximum likelihood model (33) to take sequencing errors per base into account and distinguish low-level variants from read errors. Different metrics (e.g., strand bias check, coverage checks for both strands and alleles) are applied to refine the detected variants (25). A minimum coverage of 780× was defined to detect a variant at 1% following a confidence interval for binomial proportions. A variant with a 1% mutation level (i.e., fractional representation of the variant base among all reads) can be estimated with a 95% confidence interval, which does not include 0 at a minimum coverage of 340×. Because our tool requires a mutation to be confirmed in both read directions, a total coverage of 780× is required to differentiate a variant from noise with high confidence.

The identified variants are annotated using an annotation file that provides the location of genetic elements (exons, introns, etc.) and exon reading frame. The exon reading frame is used to annotate amino acid mutations automatically. Amino acids are translated starting from the first amino acid, which is fully encoded in exon 421 (i.e., a proline) because the first base of the preceding triplet is encoded by the previous KIV repeat (i.e., in KIV-1 for the first KIV-2 repeat) and, therefore, technically unknown to our analysis pipeline. Accordingly, our amino acid numbering is shifted by −1 compared to Parson et al. (11). Therefore, the KIV-2 nonsense variant named “p.R21X” by Parson et al. (11) is named p.(Arg20Ter) in our data. Importantly, the annotation file format is a simple tab-delimited text file and allows customization of the annotation by the user. The annotation for our own reference will be constantly maintained and made available at https://github.com/genepi/lpa-pipeline.

The pipeline has been implemented on top of Cloudgene (34), a framework for executing computational pipelines and workflows graphically and on the command line. The complete pipeline can be executed locally. For reproducibility, all necessary steps and data sets have been made available at the website provided above. The positions 2472 to 2505 in the PCR5104 reference sequence were excluded from all considerations because they are located in a microsatellite.

### Genetic variability in KIV-2 in 1000 Genomes

1000 Genomes is a public data resource that includes genome sequencing data from 2,504 individuals from 26 populations worldwide. High-coverage exome sequencing data of all individuals is available as well as high-coverage whole genome sequencing (WGS) data from a subset of 150 individuals (Illumina Polaris project). We extracted all reads mapping to the *LPA* KIV-2 region (GRCh37; chr6:161,033,785–161,066,618) from these data sets and reanalyzed them using our analysis pipeline. Bioinformatic details are given in supplemental Methods, and the scripts are available at the website provided above.

### Statistics

All statistical analyses were done using R 3.3.2 (https://www.r-project.org). Proportions were tested using the two-sample test for equality of proportions as implemented in R (prop.test function). In our discovery set, we inverse-normal transformed Lp(a) concentrations for the preliminary assessment of the effects of the DraIII variant on Lp(a) concentrations to account for the highly skewed Lp(a) distribution. The concentration-determining smaller isoform present in the Western blot was used to adjust the models for the apo(a) isoform, as done previously (23, 35). The effect of KIV-2B variants was determined by assigning a KIV-2B carrier status (yes/no) to each sample based on whether the three canonical KIV-2B variants were present (*n* = 100), without further grouping based on variant level. The impact of being a KIV-2B carrier on inverse-normal transformed Lp(a) concentrations was then determined by a general linear regression model that was adjusted for the shorter isoform (35). GC percentage was calculated using the DNA/RNA GC content calculator at http://www.endmemo.com/bio/gc.php.

## RESULTS

### KIV-2A and KIV-2B plasmids and pipeline validation

Noureen et al. (12) recently published batch-sequencing amplicons that specifically amplify only the KIV-2 repeat. We used these to create plasmids containing KIV-2A and KIV-2B sequences for amplicons spanning both the interkringle intron (amplicon PCR2645) and intrakringle intron (amplicon PCR5104) (supplemental Fig. S2). The inserts were resequenced by Sanger sequencing.

Overall, the PCR5104 plasmids differed by 25 (KIV-2A) and 76 (KIV-2B) positions compared with the reference and by 73 positions from each other (supplemental Table S3; not considering indels and the microsatellite region in the central intronic region of LPA5104). Sixty variants were then defined as KIV-2B-specific (see Methods for the proper definition of the terms “KIV-2B variant” and “canonical KIV-2B variant”) and used for the evaluation of our bioinformatic variant analysis pipeline (see below). The PCR2645 plasmids showed 8 (KIV-2A) and 116 (KIV-2B) differences to the reference and 120 to each other (supplemental Table S4). The differences between KIV-2A and KIV-2B plasmids mostly matched those expected from the alignment of the six KIV-2 repeats present in the human reference genome hg19, corroborating the hg19 reference sequence in this region. All variants were also detected by MiSeq sequencing of the unmixed plasmids except the two insertions at positions 325 (AAG insertion in KIV-2B) and 353 (adenine insertion in KIV-2B).

### Bioinformatic aspects and a freely available variant-calling pipeline for KIV-2

Variants in single KIV-2 repeats behave like mitochondrial heteroplasmies in that they are present only in a subset of amplicons and respective sequencing reads. This allowed us to adapt our established mitochondrial heteroplasmy analysis pipeline [mtDNA-Server (25)] to *LPA* KIV-2 analysis. Technical adaptions of the mtDNA-Server pipeline comprised *1*) an improved variant-calling method that is specifically adapted to KIV-2 and incorporates what has been learned from the calibration experiments described below; *2*) uploading user-specified reference files (reference sequence and position annotation file); *3*) detecting multiallelic sites (i.e., more than two observed alleles at one position) that could potentially arise from superimposing all repeats to one reference; *4*) developing a standalone version; and *5*) adding a generic variant annotation step.

The complete bioinformatic analysis pipeline is freely available and has been integrated into the workflow system Cloudgene (34), which allows running the pipeline locally (thus avoiding transferring data to remote servers outside the user’s institution). Importantly, despite being an NGS analysis pipeline, it can be easily installed locally and creates a graphical, user-friendly point-and-click interface upon execution. It automatically creates tabular variant reports that annotate known variant positions as well as the effects of coding variants using a position annotation file. The annotation file for our reference sequence is preloaded in the pipeline and will be constantly maintained, but the annotation file can be easily customized to add additional annotations or to other amplicons. The preloaded annotation file also annotates the variants caused purely by the superposition of KIV-2A and KIV-2B amplicons to allow discerning genuine novel variants from simple KIV-2B variants.

### Base alignment quality recalibration has a detrimental effect on KIV-2B mutation detection

To calibrate and benchmark our pipeline, two *LPA* plasmids containing a KIV-2A and KIV-2B insert were mixed at different ratios (50:50 to 99:1), with the KIV-2B plasmid always representing the minor component to mimic the in vivo situation. As all KIV-2B-specific variants are located on the same molecule, they shall all present approximately the same mutation level (explained in supplemental Fig. S5).

In mitochondrial DNA analysis we had previously found that base alignment quality (BAQ) scoring (36) increases mutation detection precision by reducing false SNP calls caused by misalignments around indel regions (25) in the event of challenging sequence data. Conversely and surprisingly, we observed that in KIV-2 batch sequencing, BAQ reduces the sensitivity of detecting KIV-2B-specific variants (**Table 1**). This is due to the fact that several differences between subtypes A and B are represented by dinucleotide to pentanucleotide stretches of consecutive differences (see some examples in supplemental Fig. S4). When these sequences are mapped back to one single reference repeat, variants originating from different repeats are merged into one sequence and create a specific mutation pattern characterized by several consecutive low-level mutations. Such mutation patterns are deemed as highly unlikely by BAQ recalibration and are thus pruned without notice. However, in KIV‑2 batch sequencing, these patterns arise from the inevitable co-amplification of KIV-2A and KIV-2B regions and represent an intrinsic feature of the sequencing approach. Therefore, these positions are indeed genuine base variants. The use of BAQ thus has a detrimental effect on mutation detection in the KIV-2 region and should be avoided (see next section).

**TABLE 1.** Precision, sensitivity, and specificity values of our variant-calling pipeline with and without BAQ recalibration

	With BAQ			Without BAQ		
	Precision	Sensitivity	Specificity	Precision	Sensitivity	Specificity
50% Mix						
Herculase II Fusion	79.67	93.88	99.59	99.53	100	99.99
NEB LongAmp	77.76	94.12	99.54	98.28	99.41	99.97
Qiagen LongRange Kit	77.94	97.65	99.53	98.85	100	99.98
10% Mix						
Herculase II Fusion	77.65	90.98	99.56	100	99.22	100
NEB LongAmp	75.00	91.77	99.48	98.84	100	99.98
Qiagen LongRange Kit	75.85	92.35	99.50	99.42	100	99.99
5% Mix						
Herculase II Fusion	80.21	92.16	99.61	100	99.22	100
NEB LongAmp	73.33	90.59	99.44	100	100	100
Qiagen LongRange Kit	73.79	89.41	99.46	98.82	98.82	99.98
2.5% Mix						
Herculase II Fusion	67.83	54.51	99.58	100	61.57	100
NEB LongAmp	75.96	92.94	99.50	100	100	100
Qiagen LongRange Kit	64.71	51.77	99.52	98.11	61.18	99.98
1.5% Mix						
Herculase II Fusion	55.74	29.02	99.61	97.56	30.59	99.99
NEB LongAmp	75.73	91.77	99.50	100	100	100
Qiagen LongRange Kit	57.38	41.18	99.48	97.56	47.06	99.98
1% Mix						
Herculase II Fusion	61.76	28.71	99.70	100	29.41	100
NEB LongAmp	61.11	38.82	99.58	97.22	41.18	99.98
Qiagen LongRange Kit	61.36	38.24	99.59	98.65	41.18	99.99

**Fig. 1.**
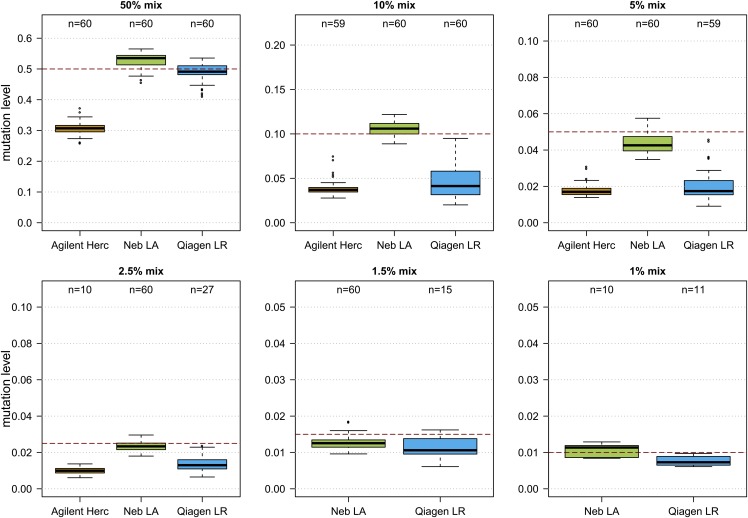
Impact of the polymerase on the KIV-2B variation level detected in plasmid mixes. Precision of the evaluated PCR polymerases in reproducing the expected mutation level of KIV-2B variants in mixes of two plasmids containing either a KIV-2A or KIV-2B insert (the rationale is detailed in supplemental Fig. S5). Sixty base differences distributed over the KIV-2B insert were evaluated (see the Methods section for an explanation of this number). Herculase II Fusion tended to underamplify the minor component in the mix (i.e., the KIV-2B variants). It did not detect any variants in the 1.5% or 1% mix. Because no level below 1.2% is expected in the population (1 mutant kringle in 80 KIV-2 repeats), the analysis pipeline requires a minimum of 1% for a variant to pass quality control to guarantee specific detection (25). Therefore, at very low mixture levels the number of variants shown in the plots decreases because some bases (e.g., those in difficult sequence contexts) may be underamplified and thus fall below the 1% threshold.

The values are given in percentages and report the performance of the pipeline in calling correctly all 60 KIV-2B variants of the PCR5104 (see Methods for an explanation of this number; see also Fig. 1, supplemental Table S1). Precision was best without BAQ.

### The PCR amplification protocol modifies the mutation level of KIV-2B variants

In the PCR5104 fragment, the template mixture experiments revealed an impact of the amplification protocol used for library template amplification on the mutation detection sensitivity. Amplification using Herculase II Fusion led to an underestimation of the variant level of all mutations located on the KIV-2B plasmid (**Fig. 1**, supplemental Fig. S1D, E), while the NEB LongAmp polymerase recapitulated the expected mutation level best. No impact of the polymerase was observed for PCR2645 (supplemental Fig. S6). No pronounced difference in the GC content was observed between KIV-2A and KIV-2B (∼44.3% vs. 44.1% in hg38; supplemental Table S5).

**Fig. 2.**
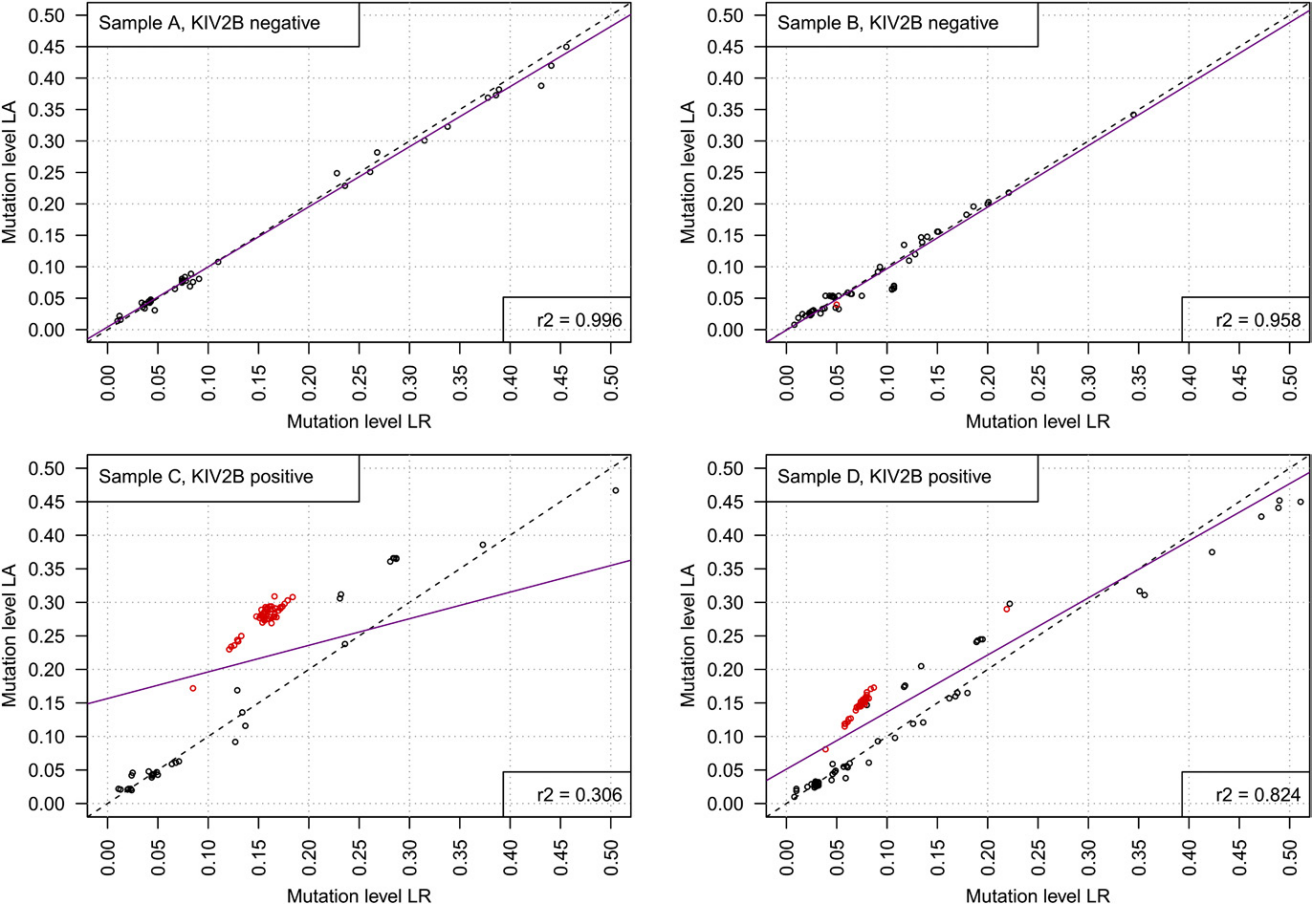
Variant levels detected in four genomic DNA samples by LA and LR. Native human samples recapitulate the differential amplification of KIV-2B. Red dots represent the 60 KIV-2B-specific variants specified in the Methods section, while black dots represent other variants in any other KIV-2 repeat detected (i.e., variants located not on amplicons stemming from KIV-2B repeats; see supplemental Fig. S1 for a detailed explanation of the rationale and mechanism). The upper panels show mutation levels of all variants detected by batch ultradeep sequencing in two SAPHIR individuals without any KIV-2B variants (i.e., no KIV-2 kringle is a KIV-2B): both polymerases give the same results. The lower panels show mutation levels of all variants detected by ultradeep sequencing in two SAPHIR individuals with KIV-2B variants at two different levels. The levels of the KIV-2B variants (red) differ between the polymerases, with LA showing levels about twice as high as LR, while the levels of non-KIV-2B variants (black) are similar between the two polymerases. Magenta: regression line.

The impact of the amplification protocol is also recapitulated in human genomic DNA samples. We amplified and deep-sequenced four human genomic samples with all three polymerases. **Figure 2** shows the variant levels detected by the polymerases NEB LongAmp and Qiagen LongRange PCR Kit. The Herculase polymerase was excluded from further considerations because it detected only very few KIV-2B variants. The correlation of the variant levels was excellent (*r*^2^ = 0.996 and *r*^2^ = 0.958) in the samples that did not have KIV-2B repeats (as defined by the detection of the respective base differences to KIV-2A) but only low to moderate (*r*^2^ = 0.306 and *r*^2^ = 0.824) in the samples that contained KIV-2B kringles at various levels. The difference in *r*^2^ in the latter samples was driven by the fact that all variants from KIV-2B (Fig. 2, red dots) were differentially amplified, while the non-KIV-2B variants (Fig. 2, black dots) remained unchanged. The two samples differed in the mean KIV-2B variation level, which is determined by the number of KIV-2 repeats present (i.e., the size of the isoform), thus skewing the regression line to different extent.

Because all KIV-2 variants are mapped to one single KIV-2 reference repeat, variations that are consecutive in the reference do not necessarily originate from the same KIV-2 repeat. This allows evaluating whether the observed shift in variation level is just sequence context-dependent or indeed originates from differential amplification of the specific KIV-2 subtype amplicons. Variants located closely together showed a concordant polymerase-based shift in variation level only if they were both KIV-2B-specific. Conversely, if two closely adjacent variations (e.g., positions 141 and 146 in supplemental Table S6) originated from different KIV-2 subtypes, the shift was observed only in the KIV-2B variant. This observation seemed to be independent from the absolute mutation level (supplemental Table S6).

### Variation patterns in the de novo-sequenced amplicon data set

We sequenced the PCR5104 fragment (spanning 92% of the KIV-2 region) in 123 samples from the Salzburg Atherosclerosis Prevention Program in Subjects at High Individual Risk (SAPHIR) (31) and the German Chronic Kidney Disease (32) study populations. To enrich for functional variants, these samples have been selected following a discordant-phenotype approach with 80 samples showing pronounced deviations from the inverse association between the number of KIV repeats and Lp(a) concentrations [i.e., low concentrations in individuals with low molecular weight apo(a) phenotypes or high concentrations in patients with high molecular weight apo(a) phenotypes], while the rest showed the expected relationship between the Lp(a) concentration and apo(a) isoform. The samples are described in detail in Coassin et al. 2017 (23).

**Fig. 3.**
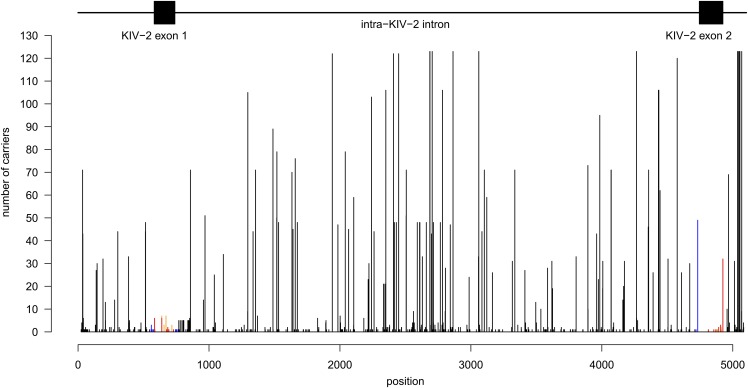
Number of discovery samples with a variant at a given position (excluding KIV-2B variants). Black: noncoding variants; blue: near-splice site variants (±25 bp; *n* = 11); orange: silent variants; and red: nonsynonymous and nonsense variants. The location of intron-exon structures is given above the histogram. The frequent missense variant at the 3′ end of exon 2 is KIV-2 4925G>A^23^. The KIV-2B variants are shown in supplemental Fig. S7.

After excluding the CA microsatellite in the center of the KIV-2 region, 513 unique single-nucleotide variants were observed in the KIV-2 region. Of these, 425 variants were novel and not caused simply by sequence differences between KIV-2A and KIV-2B or between the five different KIV-2A sequences present in the human reference genome (supplemental Table S7). Fifteen positions showed three alleles (supplemental Table S8). Several frequent intra-KIV-2 differences were observed, with some positions being variable in most to all sequenced individuals (**Fig. 3**, supplemental Fig. S7), albeit at widely varying levels (supplemental Fig. S8). Two nonsense [p.(Arg21Ter) (11), p.(Cys32Ter) (novel)], twelve nonsynonymous [including 4925G>A (23)], and eight silent variants (in addition to the three canonical KIV-2B variants) were observed (Fig. 3, supplemental Table S9, supplemental Fig. S9). When expressing mutation density as *n* variants per total exon bases sequenced (excluding the three canonical KIV-2B variants), mutation density was similar between KIV-2 exon 1 and 2 (*P* = 1.00; 11 variants in 160 bp vs. 12 variants in 182 bp). Conversely, when also considering the number of carriers for each exonic mutation (excluding the canonical KIV-2B variants) exon 2 was more variable [32 variants in 19,680 bases sequenced from exon 1 (123 samples × 160 exonic bp) vs. 59 in 22,386 bases sequenced from exon 2 (123 samples × 182 exonic bp); *P* = 0.034]. This was, however, driven by the frequent KIV-2 exon 422 variant p.(Thr114Ala) (amplicon position 4925G>A) (23).

After excluding both KIV-2B variants and 4925G>A, no significant difference in mutation density was observed between exon 1 and 2 (*P* = 0.31). The number of carriers for the exonic non-KIV-2B variants ranged from 1 to 32 (supplemental Fig. S9). All were present only in ∼1–2 repeats per individual (estimated by multiplying the observed NGS level with the sum of genomic KIV-2 repeats estimated by quantitative PCR). Excluding the KIV-2B-specific intronic variants at positions 555, 558, 564, and 565, a depletion of frequent intronic variants close to the splice site (defined as ±25 bp exon-flanking intronic sequence) was observed: 13 variants were observed within ±25 bp of the splice sites, but only 4733G>A (39.8% carrier frequency; *n* = 49) and 560T>C (*n* = 3 carriers) were present in more than 1 carrier (supplemental Fig. S9). 4733G>A is located only 10 bp upstream from the 5′ splice site of the second KIV-2 exon and appears to be rather frequent. In the 43 controls from our previous study (23), 4733G>A shows a significant association with reduced Lp(a) (*P* = 0.0052; *n* = 23 carriers) after adjusting for the apo(a) isoform and 4925G>A carrier status.

### Reliability of the genome reference sequence in the KIV-2 region

In the discovery sample, 15 positions showed average variant levels >0.5, indicating that at these positions the variant base might indeed be the wild-type base when defined by frequency (supplemental Tables S7, S10). At three amplicon positions (1942, 2449, 4576), the variant bases were even present at a >0.95 level with small variance. Because the sequence of the reference KIV-2A repeats differ among each other (supplemental Table S7, supplemental Alignment), this observation could also be caused simply by which KIV-2 repeat of the human genome reference sequence is selected as a reference for the read alignment. This indeed explains several of these positions, but not all. The amplicon positions 35, 1356, 3103, 4071, 4072, and 4358 are not present in any of the reference kringles but show an average variation level of 0.62 ± 0.25. The fact that they occurred at the same level suggests that they might mark a novel haplotype.

### 1000 Genomes data analysis

The Lp(a) trait differs markedly between ethnicities and populations (3). To gain insights in the variability of the KIV-2 region among different populations worldwide, we extracted all reads mapping to the *LPA* KIV-2 from the 1000 Genomes phase 3 exome data set (19) and the 1000 Genomes Polaris high-coverage WGS data set (37) and subjected them to our analysis pipeline. The Polaris data set is markedly smaller than the 1000 Genomes exome data set but also contains intronic sequences.

**Fig. 4.**
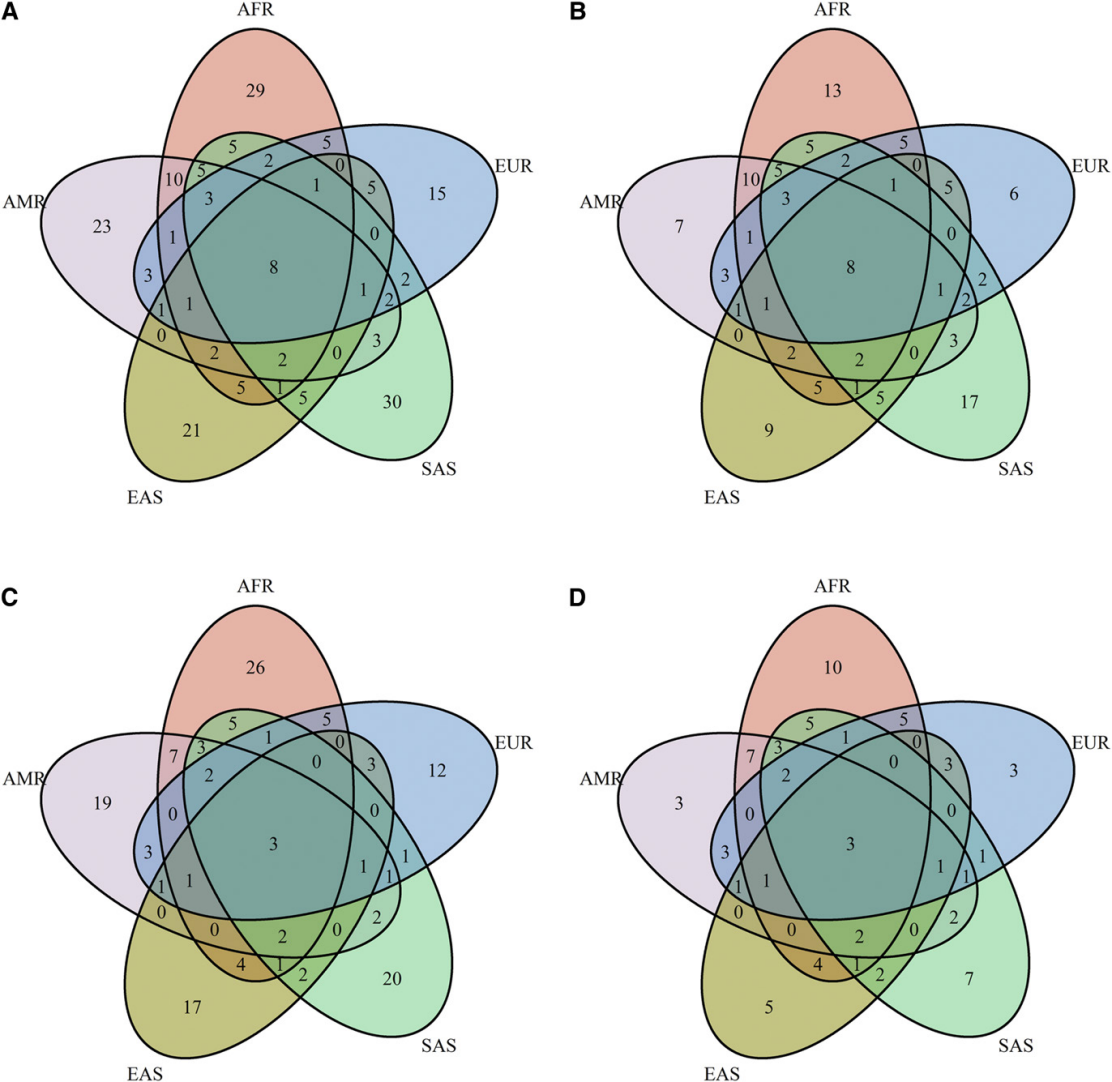
Venn diagrams for the exonic KIV-2 variation in 1000 Genomes. Venn diagram showing (on an amino acid level) the overlap of unique coding (A, B) and unique missense variants (C, D) observed in the different super populations of the 1000 Genomes exome data set. Panels A and C show all variants, while panels B and D are restricted to variants observed at least twice. A consistent portion of variants is exclusive to single populations. Variants according to supplemental Table S11. The figure based on the DNA level is shown in supplemental Fig. S13. AFR, African; AMR, Admixed American; EAS, East Asian; EUR, European; SAS, South Asian.

We observed a large amount of exonic sequence variability, including 10 nonsense, 129 nonsynonymous, and 62 silent mutations as well as 3 mutations at the canonical intronic splice-site bases (**Table 2**, supplemental Tables S9, S11). Most coding and missense variation was rare (supplemental Table S11) and exclusive to single populations (**Fig. 4**).

**TABLE 2.** Number of variants found in our study

	1000 Genomes Exome and Polaris WGS	Our Sequencing Set	Overlap
Nonsense mutations	10/9	2/2	1/1
Nonsynonymous SNPs*^a^*	129/115	12/12	12/11
Synonymous SNPs*^b^*	62/56	11/11	8/8
Canonical splice-site mutations	3/3	0/0	0/0
Noncoding SNPs*^c^*,*^d^*	1,122/937	488/488	304/278

The number of unique mutations of the respective variant classes found in the batch-sequenced discovery data set and the public 1000 Genomes data sets are shown. The numbers are reported as total number of variants observed/number of variants confirmed by at least one sample with >780× coverage at the variant position. Details for the exonic variants stratified per population are given in supplemental Tables S7 and S9.

a Includes the 4925G>A variant (23), which also causes a p.(Ala114Thr) substitution.

b Includes the three canonical KIV-2B variants.

c 1000 Genomes data are from the Polaris data set only.

d Includes the KIV-2B-specific intronic variants.

All exonic variants being present in all super populations were silent except for p.(Pro1Leu) and p.(Ala114Thr) [KIV-2 4925G>A (23)] (see supplemental Table S11 for a complete list and the number of carriers per variant). The density of variants differed between the exons and populations, with Africans and Europeans showing the highest variant density (supplemental Table S12). This was mostly driven by some variants that were frequent in the given population but (nearly) absent in the others [e.g., p.(Glu3Asp), p.(Gly7Glu), p.(Ser24Tyr), p.(Ser42Leu), p.(Thr46Ile), p.(Tyr49Cys), p.(Thr86Met), and p.(Gln87Glu) in Africans or Pro1Leu, Thr46Asn, and p.(Ala114Thr)/4925G>A (23) in Europeans]. The nonsense variants were mostly rare and exclusive to single populations. Only p.(Arg20Ter) [p.R21X in Parson et al. (11)] was present at appreciable frequencies (1.1% to 3.4%) in multiple super populations (Europeans, Admixed Americans, and South Asians) (supplemental Table S11). All canonical splice-site mutations were located at the splice sites of exon 421 (donor at exon 421 +1: 11× G>A and 1× G>C; acceptor at exon 421 −2: 3× A>C; supplemental Table S11). Interestingly, the splice-site variant KIV-2 4925G>A, which is frequent in Europeans and associated with pronounced Lp(a) reduction (23), was present in all super populations but East Asians (except for two carriers in Japanese) (supplemental Table S11).

### Frequency of KIV-2B kringles between populations

Based on the canonical KIV-2B variants in exon 1, 100 samples (81%) of our batch-sequencing discovery set of European origin carried KIV-2B kringles. No effect of being a KIV-2B carrier was observed on inverse-normal transformed Lp(a) in an isoform-adjusted model (*P* = 0.994). The KIV-2B variation levels and accordingly the number of repeats that were KIV-2B varied widely even within our relatively homogenous discovery set (supplemental Fig. S10). Due to its aggregated nature, KIV-2 batch PCR sequencing cannot distinguish between KIV-2B and KIV-2C kringles (sequence patterns given in supplemental Fig. S3) when both subtypes occur in the same sample because the positions 594 and 621 are present in both subtypes and the variant levels thus sum up. However, the third canonical KIV-2B variant at position 666 is present only in KIV-2B, and thus a lower variation level at this position could be expected. Indeed, when estimating the number of KIV-2B/C repeats in an individual by multiplying the NGS mutation level with the total number of KIV-2 repeats in the genome approximated by quantitative PCR, 18 samples show >0.8 KIV-2C repeats (supplemental Table S13). This suggests that approximately 1/7 of the discovery samples carry 1–2 KIV-2C kringles in addition to KIV-2B. The opposite situation (more KIV-2C than KIV-2B) was not observed.

**Fig. 5.**
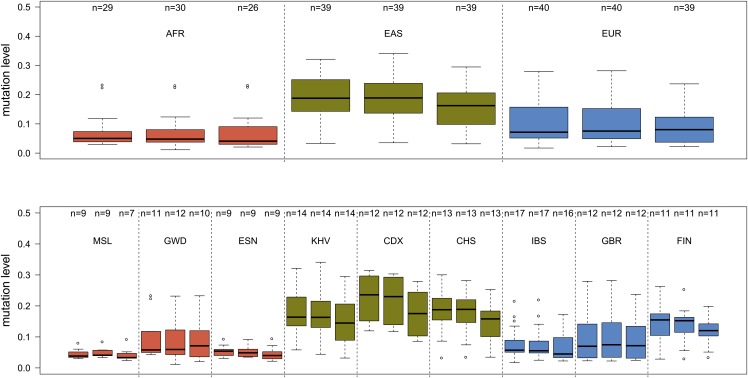
KIV-2B mutation levels in the 1000 Genomes Polaris WGS data set. The boxplots report the mutation level for each of the positions 594, 621, and 666 (from left to right) for the super populations and single populations of the Polaris WGS data set. The upper panel reveals pronounced differences in KIV-2B levels within the continental groups. Because Asians and Finns tend to present more KIV-2 repeats than Europeans (4, 12), the higher mutation levels are not just due to a lower number of KIV-2 repeats. AFR, African; CDX, Chinese Dai in Xishuangbanna, China; CHS, Southern Han Chinese; EAS, East Asian; ESN, Esan in Nigeria; EUR, European; FIN, Finnish in Finland; GBR, British in England and Scotland; GWD, Gambian in Western Divisions in the Gambia; IBS, Iberian population in Spain; KHV, Kinh in Ho Chi Minh City, Vietnam; MSL, Mende in Sierra Leone.

The in silico analysis of KIV-2 subtypes from 1000 Genomes data appeared more challenging. Several samples of the exome data set showed widely varying differences among the three canonical positions, probably due to differences in capturing efficiency and specificity, and thus this data set was not evaluated further regarding KIV-2B levels. Conversely, the data of the WGS data set appeared more stable. The intraindividual frequency of KIV-2B kringles varies between populations and was highest in Asians and lowest in Africans. Within Europeans, a clear shift from Southern Europe (Iberian population in Spain) to Northern Europe (Finnish population in Finland) was observed (**Fig. 5**, supplemental Table S14). Thirty-two samples did not present variation at position 666 but still showed variation at positions 594 and 621 (supplemental Table S14). Such samples were twice as frequent in African samples than in Europeans and East Asians (∼14% vs. ∼7%; *P* = 0.054).

### Identification of the base change underlying the KIV-2 DraIII polymorphism

Mancini et al. (24) described the occurrence of a restriction-length polymorphism in KIV-2. Specific digestion patterns have been associated with distinct levels of Lp(a), but the underlying base change has not been identified yet. Therefore, these findings cannot currently be linked with recent genome-wide data sets.

**Fig. 6.**
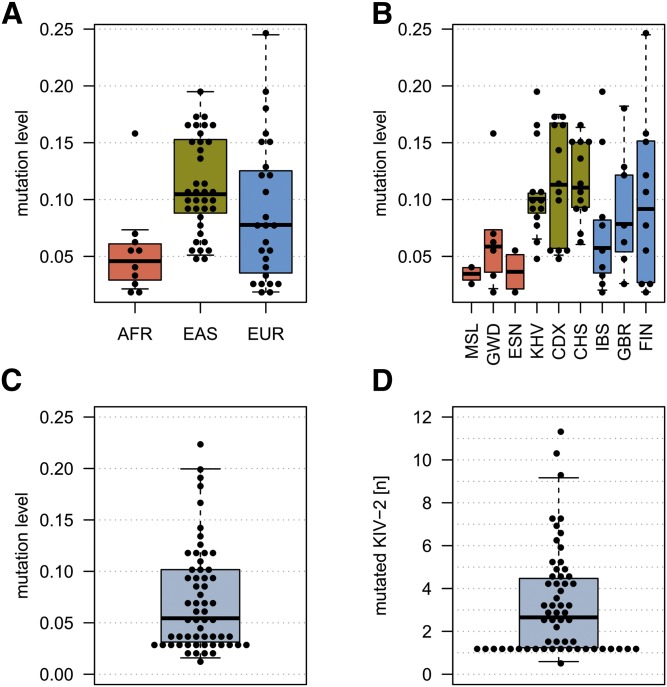
Mutation levels of the variant causing the DraIII polymorphism in different data sets. A, B: Super and single populations, respectively, of the Polaris WGS data set. C, D: Our discovery set. Each dot represents one carrier. D: Estimated number of KIV-2 repeats carrying variant 2106 based on sequencing level and PCR quantification of the sum of KIV-2. Each dot represents an individual. AFR, African; CDX, Chinese Dai in Xishuangbanna, China; CHS, Southern Han Chinese; EAS, East Asian; ESN, Esan in Nigeria; EUR, European; FIN, Finnish in Finland; GBR, British in England and Scotland; GWD, Gambian in Western Divisions in the Gambia; IBS, Iberian population in Spain; KHV, Kinh in Ho Chi Minh City, Vietnam; MSL, Mende in Sierra Leone.

We scanned the KIV-2 sequence for variants located in degenerated DraIII restriction sites. This identified a G>A change at position 2106, which creates a DraIII site. When replicating the PCR and digestion protocol of Mancini et al. (24), we indeed observed the same digestion fragments reported by Mancini et al. (24) in carriers of this variant (supplemental Fig. S11). In line with Mancini et al. (24), the DraIII/2106G>A variant was present in a largely varying number of KIV-2 copies (**Fig. 6**). Accordingly, the 1000 Genomes WGS data set also shows a pronounced heterogeneity in the variant levels at this position (Fig. 6). We finally explored in our discovery set whether the 2106G>A variant mutation level can act as an indirect readout of the DraIII restriction pattern association signal. No association of the variation level with Lp(a) concentrations was found in our limited batch-sequencing set (isoform and phenotype group-adjusted model on inverse-normal Lp(a); *P* = 0.15; *n* = 59 carriers).

## DISCUSSION

*LPA* is a major genetic risk locus for CVD (1, 2), aortic valve calcification (38), stroke (39), and probably diabetes mellitus (40), but its genetics still present several poorly understood aspects, such as the enormous interindividual and interethnic variance, variable expression and even nonexpression of single alleles, and unknown factors seemingly overriding the effect of the apo(a) isoform on Lp(a) in discordant-phenotype individuals (23). While 167 genes are necessary to explain 50% to 70% of the heritability of LDL, HDL, or total cholesterol (41), ∼90% of the Lp(a) variance is determined by one single gene locus (42, 43), namely *LPA*. Genome-wide association studies have found dozens of variants in a large region around *LPA* being associated with Lp(a) concentrations (35, 44, 45), but few causal SNPs have been mapped so far. Because the KIV-2 copy number variation can make up ∼70% of the gene sequence, most of the *LPA* gene sequence is currently not captured by available genotyping and sequencing approaches. Therefore, several variants might still hide in this region. In addition to providing novel causal variants, a mapping and finally haplotyping of this region could also contribute to a better understanding of its evolution and mutational mechanisms and even allow the imputation of KIV-2 base variants.

We provide here a framework for future in-depth studies in this region. This includes a technical assessment of the batch-sequencing protocol, a ready-to-use and easy-to-install pipeline allowing KIV-2 data analysis along with reduced bioinformatic support, a map of the variation in 123 de novo batch-sequenced samples, and variation data from an analysis of 2,504 individuals from 26 populations worldwide.

### Technical and bioinformatic aspects of mutation detection in KIV-2

To properly evaluate the sensitivity of the NGS batch-sequencing approach and calibrate the analysis pipeline, we cloned two different subtypes of KIV-2 that differ by 81 and 120 positions to each other. Sequencing of controlled mixes revealed an unexpected and surprising effect of the PCR amplification protocol on the mutation detection sensitivity, with one polymerase underamplifying specifically the KIV-2B haplotype. All templates were generated from the same plasmid template mixtures, thus excluding human error in preparing the mixtures. This suggests an intrinsic difference in the amplification efficiency of minor mixture components between the different protocols, possibly in combination with the use of a relatively long PCR fragment (because the effect was seen only in the longer of the two fragments). Such effects are recognized in expression profiling, where it is well-known that different reverse transcriptases provide different sensitivity for low-abundance transcripts (46–48), but DNA polymerases have been less extensively profiled for this aspect. Although it might be a specific feature of KIV-2 sequencing, a more general occurrence of such effects could be highly relevant for somatic mutation profiling and indicates that low-level mutation detection requires careful technical validation.

A second important technical aspect we observed was the deleterious impact of the widely used BAQ recalibration, which, triggered by the occurrence of closely adjacent variations in KIV-2 batch sequencing, erroneously prunes genuine variants without giving notice. Although the reason for this behavior can be clearly pinpointed, it might not be commonly recognized in the community. Similar adjacent mutations can also occur in other repetitive regions of the genome if sequenced by a similar approach. For example, this can be the case in somatic mutation profiling of tumor tissue. Therefore, the KIV-2 region represents a note of caution showing the importance of thoroughly validating bioinformatic procedures in low-level mutation detection because variants might escape detection even simply due to technical reasons. Indeed, we became aware of this issue only because we could rely on the clones as a gold standard.

### A freely available variant-calling pipeline for the *LPA* KIV-2 region

We provide a data analysis pipeline that already incorporates several improvements tailored to *LPA*, such as the dismissal of BAQ and capability of calling multiallelic sites. Using customizable reference data, it annotates coding variants and flags variants that simply arise from coamplifying KIV-2A and KIV-2B kringles or from differences between different types of KIV-2A repeats (supplemental Table S7 and supplemental alignment). Thereby, it prevents misinterpreting such variants as novel ones. Additional annotations will be constantly included and curated (e.g., the DraIII variant). In combination with the best-performing polymerase, our *LPA* analysis pipeline shows excellent specificity and precision (positive predictive value) down to a 1.5% mutation level, with an inflection of sensitivity at 1% due to a hard limit set at 1%. In cases in which a low-level variant is slightly underamplified and falls below 1%, it is thus pruned from the results file. Given that we do not expect variants below 1.2% (1 variant in 80 repeats), we decided to focus on specificity rather than sensitivity and therefore opted for a hard limit. Importantly, while having been designed using *LPA* as a case study, this analysis pipeline is completely customizable and can also be used for mutation detection in any other hypervariable copy number variation region with similar features as *LPA.*

### Patterns of KIV-2 variability in different populations

Using this pipeline, we first reanalyzed 123 amplicon-sequenced samples from our recent discordant-phentoype study (23) and then reassessed publicly available exome and WGS data from 1000 Genomes. After excluding variants caused by superimposing KIV-2A and KIV-2B haplotypes, we observed 426 novel variants in our discovery set. We show that previous claims of an extraordinary high conservation in the KIV-2 region (22, 28) might have been due to insufficient sensitivity of the used Sanger-sequencing approach [as has been also noted previously (12)]. We could also not confirm a mutation frequency bias between the two exons after excluding the KIV-2B variants and 4925G>A. Some variants were present at a high level in several samples (supplemental Table S10), hinting toward the existence of additional subtypes. This is also supported by the KIV-2A we cloned. The five KIV-2A repeats in the reference slightly differ from each other, but the cloned KIV-2A repeat appeared to be a mixture of the second and fourth KIV-2 repeat of the human reference sequence (showing the differences at positions 1655–1656 but no AAA at position 2505; see supplemental alignment). This indicates that even within the reference repeats probably more haplotypes exist than currently appreciated. However, overall, the reference sequence in hg19 appeared to be reliable with few complete differences to our sequencing data.

The reanalysis of the 1000 Genomes data finally revealed 203 exonic variants, and 190 (92%) of these have not been previously reported (supplemental Tables S9, S15). Of note, we were able to confirm some variants that had been discarded by previous studies as potential artifacts due to insufficient coverage in their clone banks (11). We also observed that two variants that have been found in an South African individual by cloning (12) [positions 4774 and 4801 in our data and positions 31 and 58 in Noureen et al. (12)] are indeed highly frequent in Africans but absent in other 1000 Genomes super populations (supplemental Table S11). All exonic variants were present at very low variation levels.

At some positions more than one base change has been observed. This was also the case for the two canonical splice-site mutations, which we observed, that were both located at the splice donor of exon 421 (position 741). The G>A change has been described previously (12), but not the G>C change. Both in 1000 Genomes and in our own discovery set we observed several loss-of-function variants that may contribute to the widespread observation of null alleles. Indeed, *LPA* presents in the Exome Aggregation Consortium (20) a pLi value (i.e., the probability of a gene for being intolerant for loss-of-function mutations) of 0, indicating that loss-of-function mutations might accumulate in *LPA*. This is also supported by recent phenome-wide studies on *LPA* loss-of-function mutations that did not observe deleterious phenotypes (49). Finally, we observed different levels of KIV-2B haplotype variants among the 26 1000 Genomes Project populations and even a shift within Europe (Fig. 5). This might support differences in the genetic history of the KIV-2 repeat. Given that KIV-2B levels are lowest in populations with (relatively) high Lp(a) (Africa, Southern Europe) and highest in populations with relatively low Lp(a) (Asia, Northern Europe), it could be speculated that they are etiologically linked to hitherto unknown Lp(a)-lowering variants that cause the different Lp(a) levels between populations.

Finally, we were able to pinpoint the base change underlying the DraIII restriction polymorphism (24). This polymorphism has been reported to be present in a subset of kringles and generates several different restriction fragment combinations, some of which are associated with very distinct Lp(a) levels, but the causal variant has not been identified. As for several pre-genome era publications, the link to current genome-scale studies is, however, difficult to establish. The identification of the underlying base and the availability of modern technology now allow linking old KIV-2 data to new study designs may open new research opportunities. Indeed, in accordance with Mancini et al. (24), we found a large variance in the number of mutation-bearing kringles, although we observed only a suggestive association with Lp(a) in our limited data set. Larger data sets will be required to establish whether the SNP-based determination of DraIII may act (incompletely) as a high throughput-accessible surrogate for the various digestion patterns.

### Limitations of the study

Given the size and complexity of the *LPA* KIV-2 region, our study also inevitably presents some limitations that warrant mentioning. Our discovery set has been selected in the course of a discordant-phenotype approach study (23) and is thus not unbiased. Nevertheless, it provides data for >100 individuals. This makes it the largest data set on KIV-2 variation available until now, providing the first map of genetic variation in this nearly unaddressed genomic region. We are confident that it still captures the location of most frequent variants and will ease future experimental designs in this complex genome region. Moreover, we complemented the discovery data set with two unbiased 1000 Genomes data sets that are definitely not influenced by a selection process due to certain Lp(a) concentrations or KIV repeat numbers. Each of the three data sets has advantages and drawbacks. The discovery data set is relatively small but has Lp(a) phenotype data available and is highly specific to the KIV-2 region because it is amplicon-based. The 1000 Genomes exome data is very large but restricted to the exons and probe capturing-based, which could bias mutation levels. Thus, it provides a comprehensive census of KIV-2 variants rather than absolute precision in the mutation levels. Generally, without information about the number of KIV-2 repeats, these mutation levels are of limited utility. Finally, the 1000 Genomes Polaris WGS data set is smaller than the exome data set, but it is more unbiased and also contains intronic data.

Importantly, calling variants from the 1000 Genomes data presents the limitation that (nearly) identical kringles (supplemental Fig. S12) may not be properly differentiated by the alignment algorithm and reads may thus also map with mismatches to the wrong kringle. These mismatches can cause genuine interkringle differences to be called as KIV-2 mutations. However, such false variants would appear in every sample. This appears not to be the case. Moreover, the canonical KIV-2B variants are also the wild-type bases in KIV-3, but we still have not observed any KIV-2B variants in ∼15% of the exome samples. Similarly, not every exonic difference between KIV-1 to KIV-10 is found in the data. For example, 584C>T could also be caused by an admixture of KIV-5 sequences, but this would also cause the change 597 G>C, which is, however, not observed (supplemental Table S9). Therefore, although it cannot be completely excluded that some variants may arise from an admixture of other kringles and this may even be inevitable if a read shows 100% homology to KIV-2, we are confident that most variants will still be genuine. In supplemental Table S9, we explicitly annotate each exon position with information regarding whether the observed base is the wild-type base in another kringle and which other bases are found at these positions in other kringles.

## CONCLUSION

Our study represents a first census of variation in the highly complex *LPA* KIV-2 region. It provides the first reference data set for variation patterns in this complex region and reveals a large amount of potentially functional variation in a region that encompasses up to 70% of the coding sequence of *LPA*. Our free variant-calling pipeline with an easy-to-use graphic interface now also allows similar studies in laboratories without access to strong bioinformatic support. It will thus support future studies aiming to map causal variants that create extreme Lp(a) levels or underlie the many independent genome-wide association study hits (35) in the *LPA* region.

Importantly, our observations also extend beyond Lp(a) research. Coding copy number variations are still elusive and poorly captured by genome-wide sequencing or genotyping experiments due to the ambiguous mapping of reads or probes. In this article, we comprehensively and thoroughly worked through such a clinically highly relevant copy number variation region. Our observations may also provide input to researchers investigating similar genes containing hypervariable coding copy number variations and/or highly homologous regions such as *MUC1* (50) (i.e., tumor marker CA-15-3), *MUC5AC* (51), *KCNJ12*/*KCNJ18* (52), or *CT47* (53).

## Supplementary Material

Supplemental Data
