# Supplementary material for: A comprehensive map of single-base polymorphisms in the hypervariable LPA kringle IV type 2 copy number variation region
Source: J Lipid Res. 2018 Nov 9;60(1):186–99. doi: 10.1194/jlr.M090381 (PMC6314250; doi:10.1194/jlr.M090381)
Supplement: Supplemental Data [file supp_60_1_186__index.html]

A comprehensive map of single base polymorphisms in the hypervariable LPA Kringle IV-2 copy number variation region — A comprehensive map of single-base polymorphisms in the hypervariable LPA kringle IV type 2 copy number variation region — Supplemental Data 

# A comprehensive map of single-base polymorphisms in the hypervariable *LPA* kringle IV type 2 copy number variation region

## Supplemental Data

- Supplemental Material (.pdf, 1.3 MB) - Entire File mit Supplemental Material
