# Supplementary material for: A comprehensive map of single-base polymorphisms in the hypervariable LPA kringle IV type 2 copy number variation region
Source: J Lipid Res. 2018 Nov 9;60(1):186–99. doi: 10.1194/jlr.M090381 (PMC6314250; doi:10.1194/jlr.M090381)
Supplement: Supplemental Data [file 10.1194_M090381_jlr.M090381-1.pdf]

SUPPLEMENTARY MATERIALS TO:

A comprehensive map of single base polymorphisms in the hypervariable *LPA* Kringle-IV-2 copy number variation region

Running title: Sequencing of the LPA KIV-2 repeat

Stefan Coassin [1*], Sebastian Schönherr [1*], Hansi Weissensteiner [1], Gertraud Erhart [1], Lukas Forer [1], Jamie Lee Losso [1], Claudia Lamina [1], Margot Haun [1], Gerd Utermann [2], Bernhard Paulweber [3], Günther Specht [4], Florian Kronenberg [1,5 §]

* shared first author

§ corresponding author

[1] Division of Genetic Epidemiology, Department of Medical Genetics, Molecular and Clinical Pharmacology, Medical University of Innsbruck, Innsbruck, Austria
[2] Division of Human Genetics, Department of Medical Genetics, Molecular and Clinical Pharmacology, Medical University of Innsbruck, Innsbruck, Austria
[3] First Department of Internal Medicine, Paracelsus Private Medical University, Salzburg, Austria
[4] Department of Database and Information Systems, Institute of Computer Science, University of Innsbruck, Innsbruck 6020, Austria
[5] The German Chronic Kidney Disease study

Address of correspondence:
Florian Kronenberg, MD
Division of Genetic Epidemiology
Department of Medical Genetics, Molecular and Clinical Pharmacology
Medical University of Innsbruck
Schöpfstr. 41, A-6020 Innsbruck, AUSTRIA
Phone: (+43) 512 9003-70560, Fax: (+43) 512 9003-73560 or -73561
E-mail: Florian.Kronenberg@i-med.ac.at

1 Supplemental Tables

Supplemental Table S1: PCR conditions batch amplification.

DNA concentrations and PCR cycles are given for the calibration plasmids; for details on the discovery sample set see ref. (1).

	LPA5104	LPA5104	LPA5104	LPA2645	LPA2645	LPA2645
**Product length**	5,104	5,104	5,104	2,645	2,645	2,645
**Reaction volume for values given below [µl]**	20	25	25	50	25	60
**Enzyme**	Agilent Herculase II Fusion	NEB LongAmp	Qiagen LR PCR Kit	Agilent Herculase II Fusion	NEB LongAmp	Qiagen LR PCR Kit
**Initial denaturation**	95 °C, 2 min	94 °C, 3 min	93 °C, 2 min	95 °C, 2 min	94°C, 3 min	93 °C, 3 min
**Denaturation**	95 °C, 20 sec	94 °C, 30 s	93 °C, 15 s	95 °C, 20 s	94°C, 30 sec	93 °C, 15 sec
**Annealing**	62.8 °C, 20 sec	64 °C, 30 s	64.5 °C, 30 s	56 °C, 20 s	55°C, 30 sec	56, °C 30 sec
**Extension**	72 °C, 3 min	65 °C, 5 min	68 °C, 5 min	72 °C, 1:45 min	65°C, 1:45 min	68 °C, 3 min
**Final extension**	72 °C, 3 min	65 °C, 10 min	68 °C, 10 min	72 °C, 3 min	65°C, 10 min	68 °C, 10 min
**Number of cycles**	30	30	35	30	35	35
**ID Primer fw** [1]	421U_2	421U_2	421U_2	422U	422U	422U
**ID Primer rv** [1]	422L	422L	422L	421L	421L	421L
**Final primer conc. [µM each]**	0.25	0.4	0.6	0.25	0.4	0.6
**Final dNTP conc. [mM]**	0.25	0.3	0.5	0.25	0.3	0.5
**Enzyme mix [µl]**	0.4	1	0.25	1	1	0.48
**DNA input [ng]**	8	8	8	8	8	16

[1] **Primer sequences are given in Supplemental Table S2**

Supplemental Table S2: Primer sequences

Primer ID	Sequence 5' – 3'	Citation
**421U**	TTGGCTTTCATGATCAACG	(2)
**421L**	TTTTTCTGACAATCGGAATATAC	(2)
**421U_2**	TCAGGATGCAGGGCATGAG	(2)
**422L**	CACCAGAAATCACTCCGCTG	(2)
**422U**	AGAAACAAACCTACTAAACCTGACAG	(2)

Supplemental Table S3: Differences in plasmids PCR5104 as determined by Sanger sequencing.

Position	Reference base	Plasmid KIV-2A	Plasmid KIV-2B	defined as KIV2-B (for calibration experiment)
21	G	G	A	yes
31	T	T	C	yes
35	C	G	C	no
50	C	C	G	yes
70	C	C	T	yes
91	T	T	C	yes
105	C	C	T	yes
112	C	C	T	yes
132	C	C	T	yes
141	A	A	G	yes
166	G	G	A	yes
197	G	G	T	yes
202	A	A	C	yes
203	A	A	T	yes
204	A	A	C	yes
213	C	C	T	yes
236	G	G	T	yes
239	C	C	T	yes
254	G	G	A	yes
260	G	G	C	yes
265	C	C	T	yes
271	G	G	C	yes
287	C	C	A	yes
302	C	C	T	yes
311	T	T	G	yes
318	C	C	G	yes
321	A	A	G	yes
325.1	:	:	A	no
325.2	:	:	A	no
325.3	:	:	G	no
328	A	A	G	yes
333	A	A	T	yes
340	G	G	C	yes
346	C	C	T	yes
353.1	:	:	A	no
355	G	G	T	yes
373	A	A	T	yes
395	A	A	T	yes

Position	Reference base	Plasmid KIV-2A	Plasmid KIV-2B	defined as KIV2-B (for calibration experiment)
399	G	G	T	yes
410	T	T	A	yes
412	C	C	G	yes
417	G	G	C	yes
421	T	T	A	yes
427	G	G	T	yes
433	C	C	T	yes
443	A	A	C	yes
458	T	T	C	yes
459	G	G	A	yes
461	A	A	G	yes
467	A	A	G	yes
473	G	G	T	yes
478	G	G	T	yes
514	C	C	T	yes
516	A	G	A	no
529	G	G	A	yes
534	T	T	G	yes
535	C	C	T	yes
539	G	G	T	yes
555	C	C	:	no
558	G	G	T	yes
564	C	C	T	yes
565	C	C	T	yes
594	A	A	G	yes
621	T	T	C	yes
666	A	A	T	yes
859	A	G	A	no
1356	T	C	T	no
1532	A	G	A	no
1655	T	:	:	no
1656	G	:	:	no
1942	A	T	T	no
2409	A	T	T	no
2416	G	A	G	no
2449	T	C	C	no
2498	A	A	:	no
2499	C	C	:	no
2500	A	A	:	no

Position	Reference base	Plasmid KIV-2A	Plasmid KIV-2B	defined as KIV2-B (for calibration experiment)
2501	C	C	:	no
2502	A	A	:	no
2503	C	C	:	no
2505	A	:	A	no
2506	A	:	A	no
2591	A	G	A	no
2609	G	A	G	no
2611	C	G	C	no
2660	A	G	A	no
2687	A	G	G	no
2688	T	G	G	no
2705	A	G	G	no
2714	G	C	G	no
2784	T	T	C	yes
3061	G	A	A	no
3103	G	C	G	no
3336	A	G	A	no
4071	A	C	C	no
4072	C	A	A	no
4265	C	T	T	no
4355	T	T	C	yes
4358	A	G	G	no
4576	C	G	G	no

Supplemental Table S4: Differences in plasmids PCR2645 as determined by Sanger sequencing.

See the Methods section of the main manuscript for the definition of "KIV-2B-specific variants" for the calibration experiment.

Position	Reference base	Plasmid KIV-2A	Plasmid KIV-2B	defined as KIV-2B (for calibration experiment)
143	C	T	T	no
236	A	G	A	no
314	A	A	G	yes
915	T	T	G	yes
923	A	A	G	yes
930	A	A	T	yes
933	A	A	G	yes
947	C	C	T	yes
987	G	G	T	yes
991	T	T	G	yes
1008	G	G	A	yes
1022	C	C	T	yes
1030	G	G	T	yes
1034	C	C	G	yes
1036	A	A	G	yes
1042	A	A	C	yes
1045	T	T	C	yes
1052	T	T	C	yes
1053	G	G	C	yes
1056	G	G	C	yes
1062	C	C	T	yes
1069	C	C	T	yes
1079	C	C	T	yes
1080	C	C	T	yes
1102	A	A	G	yes
1118	C	C	G	yes
1120	C	C	T	yes
1127	C	C	T	yes
1128	A	A	G	yes
1129	A	A	T	yes
1130	G	G	C	yes
1131	C	C	G	yes
1139	C	G	C	no
1145	C	C	T	type_b
1170	T	T	G	type_b
1175	A	A	T	type_b
1179	A	G	A	no
1198	C	C	T	yes
1199	T	T	C	yes

Position	Reference base	Plasmid KIV-2A	Plasmid KIV-2B	defined as KIV-2B (for calibration experiment)
1200	G	G	A	yes
1202	G	G	A	yes
1226	C	C	T	yes
1242	G	C	C	no
1254	G	G	A	yes
1262	G	G	A	yes
1266	C	C	T	yes
1287	C	C	T	yes
1299	G	G	A	yes
1318	C	C	T	yes
1349	A	A	G	yes
1371	A	A	G	yes
1404	A	A	G	yes
1405	G	G	T	yes
1415	C	C	T	yes
1418	A	A	C	yes
1426	A	A	G	yes
1451	G	G	A	yes
1461	T	T	C	yes
1465	C	G	C	no
1480	C	C	G	yes
1500	C	C	T	yes
1521	T	T	C	yes
1535	C	C	T	yes
1542	C	C	T	yes
1562	C	C	T	yes
1571	A	A	G	yes
1596	G	G	A	yes
1627	G	G	T	yes
1632	A	A	C	yes
1633	A	A	T	yes
1634	A	A	C	yes
1643	C	C	T	yes
1666	G	G	T	yes
1669	C	C	T	yes
1684	G	G	A	yes
1690	G	G	C	yes
1695	C	C	T	yes
1701	G	G	C	yes
1717	C	C	A	yes
1732	C	C	T	yes
1741	T	T	G	yes
1748	C	C	G	yes
1751	A	A	G	yes
1755.10	:	:	A	no
1755.20	:	:	A	no
1756.10	:	:	G	no

Position	Reference base	Plasmid KIV-2A	Plasmid KIV-2B	defined as KIV-2B (for calibration experiment)
1758	A	A	G	yes
1763	A	A	T	yes
1770	G	G	C	yes
1776	C	C	T	yes
1783.10	:	:	A	no
1785	G	G	T	yes
1803	A	A	T	yes
1825	A	A	T	yes
1829	G	G	T	yes
1840	T	T	A	yes
1842	C	C	G	yes
1847	G	G	C	yes
1851	T	T	A	yes
1857	G	G	T	yes
1863	C	C	T	yes
1873	A	A	C	yes
1888	T	T	C	yes
1889	G	G	A	yes
1891	A	A	G	yes
1897	A	A	G	yes
1903	G	G	T	yes
1908	G	G	T	yes
1944	C	C	T	yes
1946	A	G	A	no
1959	G	G	A	yes
1964	T	T	G	yes
1965	C	C	T	yes
1969	G	G	T	yes
1985	C	C	:	yes
1988	G	G	T	yes
1994	C	C	T	yes
1995	C	C	T	yes
2024	A	A	G	yes
2051	T	T	C	yes
2096	A	A	T	yes
2289	A	G	A	no

Supplemental Table S5: GC content of the PCR5104 fragment of the 6 reference KIV-2 repeats in the human genome reference sequence hg19.

Calculated using http://www.endmemo.com/bio/gc.php

Region	GC [%]	Length	Region	GC [%]	Length
**KIV-2 repeat 1**	44.36	5099	**KIV-2 repeat 4**	44.32	5096
**KIV-2 repeat 2**	44.4	5096	**KIV-2 repeat 5**	44.37	5098
**KIV-2 repeat 3 (KIV-2B)**	44.12	5101	**KIV-2 repeat 6**	44.33	5104

Supplemental Table S6: Comparison of the variation levels measured by two different polymerases in genomic DNA samples.

Comparison of the variation levels measured by two different polymerases in the region 1-666 of the PCR5104 fragment (most KIV-2B variants are clustered in this region, see Supplemental Figure S6) in four genomic DNA samples. The levels measured by one polymerase markedly differs from the other polymerase when a variant is located on the KIV-2B haplotype, but is virtually unchanged if the variant is *not* located on the KIV-2B haplotype. The effect is unlikely to be a purely sequence context-specific, as visible at closely located variants like e.g. positions 141 and 146 or positions 271-287. LA: NEB LongAmp Taq DNA Polymerase; LR QIAGEN LongRange PCR Kit.

Pos	Is a KIV-2B variant?	Sample 1		Sample 2		Sample 3		Sample 4	
		Neb LA	Qiagen LR	Neb LA	Qiagen LR	Neb LA	Qiagen LR	Neb LA	Qiagen LR
21	yes			0.281	0.155			0.157	0.081
31	yes			0.284	0.156			0.160	0.081
35	**no**	**0.999**	**0.999**			**0.999**	**0.999**	**0.453**	**0.491**
37	**no**	**0.032**	**0.048**			**0.027**	**0.035**		
50	yes			0.280	0.156			0.156	0.080
70	yes			0.279	0.156			0.154	0.079
91	yes			0.290	0.160			0.163	0.081
105	yes			0.286	0.158			0.154	0.079
112	yes			0.285	0.157			0.152	0.079
132	yes			0.287	0.157			0.157	0.079
141	yes			0.294	0.158			0.160	0.080
146	**no**			**0.021**	**0.025**			**0.029**	**0.029**
166	yes			0.291	0.158			0.153	0.079
191	**no**			**0.048**	**0.050**			**0.120**	**0.127**

Pos	Is a KIV-2B variant?	Sample 1		Sample 2		Sample 3		Sample 4	
		Neb LA	Qiagen LR	Neb LA	Qiagen LR	Neb LA	Qiagen LR	Neb LA	Qiagen LR
197	yes			0.285	0.156			0.153	0.079
202	yes			0.278	0.152			0.148	0.075
203	yes			0.280	0.149			0.149	0.075
204	yes			0.283	0.154			0.151	0.076
213	yes			0.290	0.154			0.153	0.075
236	yes			0.310	0.167			0.167	0.081
239	yes			0.287	0.160			0.151	0.077
254	yes			0.291	0.163			0.155	0.079
260	yes			0.281	0.153			0.147	0.073
265	yes			0.295	0.164			0.155	0.079
271	yes			0.295	0.162			0.155	0.079
280	**no**	**0.109**	**0.111**			**0.026**	**0.026**	**0.027**	**0.031**
287	yes			0.293	0.160			0.152	0.079
302	yes				0.157			0.152	0.077
304	**no**					**0.036**	**0.050**		
311	yes				0.153			0.146	0.074
318	yes			0.235	0.124			0.120	0.060
321	yes			0.231	0.122			0.116	0.059
328	yes			0.173	0.086			0.082	0.040
333	yes			0.243	0.131			0.127	0.063
340	yes			0.245	0.130			0.123	0.062
346	yes			0.251	0.134			0.128	0.065
355	yes			0.237	0.127			0.120	0.059
373	yes			0.291	0.165			0.156	0.078
386	**no**	**0.230**	**0.237**			**0.149**	**0.141**	**0.166**	**0.171**
392	**no**							**0.033**	**0.033**
395	yes			0.292	0.168			0.156	0.078

Pos	Is a KIV-2B variant?	Sample 1 Neb LA	Sample 1 Qiagen LR	Sample 2 Neb LA	Sample 2 Qiagen LR	Sample 3 Neb LA	Sample 3 Qiagen LR	Sample 4 Neb LA	Sample 4 Qiagen LR
399	yes			0.292	0.168			0.156	0.077
410	yes			0.271	0.155			0.140	0.070
412	yes			0.277	0.160			0.147	0.073
417	yes			0.274	0.157			0.145	0.071
421	yes			0.276	0.157			0.144	0.071
427	yes			0.283	0.167			0.150	0.076
433	yes			0.294	0.173			0.158	0.079
443	yes			0.292	0.173			0.154	0.078
458	yes			0.012					
467	yes			0.279	0.163			0.147	0.073
473	yes			0.289	0.171			0.152	0.077
478	yes			0.295	0.175			0.157	0.080
514	yes			0.299	0.177			0.158	0.083
515	**no**					**0.054**	**0.048**		
516	**no**	**0.252**	**0.262**			**0.101**	**0.094**		
529	yes			0.276	0.161			0.146	0.075
539	yes			0.279	0.166			0.149	0.079
555	yes				0.168			0.156	0.080
564	yes			0.279	0.169			0.155	0.079
565	yes			0.280	0.167			0.156	0.079
594	yes			0.304	0.180			0.172	0.085
621	yes			0.309	0.185			0.174	0.088
640	**no**							**0.029**	**0.029**
666	yes			0.270	0.164			0.148	0.075

Supplemental Table S7: Variants, which likely stem from differences between the various kringle repeats of the human genome reference sequence hg19 (without the KIV-2B positions).

See also Supplemental Alignment File. SD: standard deviation.

Pos	Ref	Variant	observed variant level in carriers (mean)	observed variant level in carriers (SD)	number of carriers
1942	**A**	T	0.98	0.02	122
2041	**A**	G	0.05	0.02	79
2241	**C**	A	0.11	0.06	103
2351	**C**	G	0.24	0.10	106
2409	**A**	T	0.68	0.20	122
2449	**T**	C	0.96	0.04	122
2687	**A**	G	0.74	0.16	123
2688	**T**	G	0.75	0.16	123
2705	**A**	G	0.60	0.17	123
2784	**T**	A	0.43	0.16	106
2864	**G**	A	0.38	0.19	123
3061	**G**	A	0.60	0.24	123
3122	**A**	C	0.04	0.02	59
3961	C	T	0.03	0.01	43
4265	**C**	T	0.64	0.21	123
4358	**A**	G	0.62	0.25	71
4433	**C**	T	0.11	0.06	106
4436	**A**	G	0.12	0.06	106
4576	**C**	G	0.96	0.05	120
5037	**T**	G	0.14	0.07	123
5045	**A**	G	0.14	0.07	123
5052	**A**	T	0.14	0.07	123
5055	**A**	G	0.14	0.07	123
5069	**C**	T	0.13	0.08	123

Supplemental Table S8: Triallelic positions

Pos	reference	alternate allele 1	alternate allele 2
481	G	A	T
640	C	A	T
853	C	G	T
1110	C	G	T
2002	C	A	T
2222	G	A	C
2241	C	A	G
2376	T	C	G
2555	A	C	G
2622	G	A	C
2858	A	C	G
2920	C	A	G
3312	G	A	C
3640	C	A	G
5018	C	G	T

Supplemental Table S9: Exonic variants detected.

The number of observations and the coverage of each variant is given. The table contains the variants both from the de-novo sequenced discovery set and the 1000G data. Empty cells mean, that the respective variant has not been observed in the respective dataset. For variants observed multiple times, the maximum coverage (supporting the validity of a variant) is given. A coverage >780X can be considered as high confidence variant. The column "Prev. cit." reports previous descriptions of the variant. The variants at position 594, 621 and 666 define the KIV-2 subtypes B and C(3, 4). . Pos.: position; Var.: variant base; cov.: coverage; “conserved” means that all KIV show the same base at the given position. ssSNP: splice site SNP, nsSNP: non-synonymous SNP, sSNP: synonymous SNP

Pos	Ref	Var	Prev. cit.	Locus	Amino acid	Variant type	Discovery set [n, max. cov.]	1000G Exome [n ; max. cov.]	1000G Polaris WGS [n ; max. cov.]	Maximum coverage observed in 1000 Genomes	Variant base is wild type in another kringle	Other base differences present at this position in any other kringle IV (KIV-1; KIV-3 to -10)
579	A	C		exon 421	splice site	ssSNP		2 ; 15855	1 ; 1129	15855	no	conserved
583	C	A		exon 421	Pro1Thr	nsSNP		1 ; 1078		1078	no	G in KIV10
584	C	T		exon 421	Pro1Leu	nsSNP	6 ; 122218	32 ; 34750	3 ; 1151	34750	KIV-5	
585	G	A		exon 421	Pro1Pro	sSNP		4 ; 31650		31650	KIV-1, 4, 6-10	
585	G	T		exon 421	Pro1Pro	sSNP		1 ; 27028		27028	no	A in KIV-1, 4, 6-10
587	C	T	(3)	exon 421	Thr2Ile	nsSNP	1 ; 41954	4 ; 49798	1 ; 822	49798	no	conserved
589	G	A		exon 421	Glu3Lys	nsSNP		2 ; 57845		57845	no	conserved
591	G	T		exon 421	Glu3Asp	nsSNP		41 ; 33955	3 ; 871	33955	KIV-8	
594	A	G	(2–4)	exon 421	Gln4Gln	sSNP	100 ; 155565	2134 ; 353223	108 ; 1179	353223	KIV3, KIV-2B	
597	G	T		exon 421	Arg5Ser	nsSNP		2 ; 9733		9733	no	C in KIV1, 5-10
598	C	T		exon 421	Pro6Ser	nsSNP		2 ; 18590	2 ; 1075	18590	no	A in KIV8
602	G	A		exon 421	Gly7Glu	nsSNP		47 ; 40089	7 ; 890	40089	no	T in KIV1, 9,10; C in KIV7
605	T	G		exon 421	Val8Gly	nsSNP		1 ; 14133		14133	no	

Pos	Ref	Var	Prev. cit.	Locus	Amino acid	Variant type	Discovery set [n, max. cov.]	1000G Exome [n ; max. cov.]	1000G Polaris WGS [n ; max. cov.]	Maximum coverage observed in 1000 Genomes	Variant base is wild type in another kringle	Other base differences present at this position in any other kringle IV (KIV-1; KIV-3 to -10)
606	G	T		exon 421	Val8Val	sSNP		6 ; 24026		24026	no	C in KIV1, 6-10; A in KIV5
606	G	A		exon 421	Val8Val	sSNP		6 ; 24026	1 ; 857	24026	KIV5	
607	C	T		exon 421	Gln9X	nonsense		1 ; 6978		6978	no	conserved
607	C	G		exon 421	Gln9Glu	nsSNP		1 ; 19757		19757	no	conserved
608	A	G		exon 421	Gln9Arg	nsSNP		3 ; 28306		28306	KIV10	
609	G	A		exon 421	Gln9Gln	sSNP		2 ; 17571		17571	no	conserved
611	A	G		exon 421	Glu10Gly	nsSNP		1 ; 16808		16808	no	conserved
611	A	C		exon 421	Glu10Ala	nsSNP		1 ; 17538	1 ; 1165	17538	no	conserved
615	C	T		exon 421	Cys11Cys	sSNP		2 ; 14393		14393	no	conserved
618	C	A	(3)	exon 421	Tyr12X	nonsense		1 ; 981		981	no	conserved
619	C	A		exon 421	His13Asn	nsSNP		1 ; 1014	1 ; 975	1014	no	T in KIV5
619	C	T		exon 421	His13Tyr	nsSNP		4 ; 18322		18322	KIV5	
620	A	G		exon 421	His13Arg	nsSNP		1 ; 18878		18878	KIV8	
621	T	C	(2–4)	exon 421	His13His	sSNP	100 ; 150590	2175 ; 386463	109 ; 1180	386463	KIV3-5	A in KIV8
622	G	A		exon 421	Gly14Ser	nsSNP		3 ; 27357		27357	no	C in KIV5
623	G	T		exon 421	Gly14Val	nsSNP		4 ; 18432		18432	no	A in KIV5
624	T	C		exon 421	Gly14Gly	sSNP		1 ; 879		879	no	
624	T	G		exon 421	Gly14Gly	sSNP		5 ; 27664		27664	no	A in KIV4
625	A	G		exon 421	Asn15Asp	nsSNP		2 ; 12622		12622	KIV1, 6, 7-9	
626	A	G		exon 421	Asn15Ser	nsSNP		1 ; 4961		4961	no	conserved
627	T	C		exon 421	Asn15Asn	sSNP		1 ; 982		982	no	conserved
629	G	A		exon 421	Gly16Glu	nsSNP		1 ; 996		996	no	conserved
635	G	C		exon 421	Ser18Thr	nsSNP		1 ; 26082		26082	no	conserved
635	G	T		exon 421	Ser18Ile	nsSNP		1 ; 284		284	no	conserved
640	C	A		exon 421	Arg20Arg	sSNP	1 ; 4354	2 ; 12503		12503	no	conserved
640	C	T	(3)	exon 421	Arg20X	nonsense	6 ; 80116	25 ; 27783	2 ; 922	27783	no	conserved
641	G	C		exon 421	Arg20Pro	nsSNP		1 ; 494		494	no	A in KIV4
641	G	A		exon 421	Arg20Gln	nsSNP		3 ; 17500		17500	KIV4	

Pos	Ref	Var	Prev. cit.	Locus	Amino acid	Variant type	Discovery set [n, max. cov.]	1000G Exome [n ; max. cov.]	1000G Polaris WGS [n ; max. cov.]	Maximum coverage observed in 1000 Genomes	Variant base is wild type in another kringle	Other base differences present at this position in any other kringle IV (KIV-1; KIV-3 to -10)
642	A	C		exon 421	Arg20Arg	sSNP		1 ; 8137		8137	no	conserved
643	G	C		exon 421	Gly21Arg	nsSNP		4 ; 37213		37213	no	conserved
644	G	T		exon 421	Gly21Val	nsSNP		1 ; 546		546	no	conserved
644	G	A		exon 421	Gly21Asp	nsSNP		6 ; 19999		19999	no	conserved
645	C	G		exon 421	Gly21Gly	sSNP		1 ; 14096	1 ; 889	14096	no	conserved
645	C	T		exon 421	Gly21Gly	sSNP		1 ; 996		996	no	conserved
647	C	T		exon 421	Thr22Ile	nsSNP		2 ; 823		823	KIV10	
649	T	C	(2, 3)	exon 421	Tyr23His	nsSNP		1 ; 7274		7274	KIV8	
652	T	A		exon 421	Ser24Thr	nsSNP		1 ; 768		768	no	conserved
653	C	A		exon 421	Ser24Tyr	nsSNP		38 ; 34559	3 ; 908	34559	no	T in KIV4
656	C	A		exon 421	Thr25Asn	nsSNP		1 ; 953		953	no	T in KIV4
657	C	A		exon 421	Thr25Thr	sSNP	3 ; 86493	12 ; 33126	1 ; 835	33126	no	T in KIV4
657	C	T		exon 421	Thr25Thr	sSNP		26 ; 29742	1 ; 835	29742	KIV4	
661	G	C		exon 421	Val27Leu	nsSNP		3 ; 19124		19124	no	A in KIV8
663	C	A		exon 421	Val27Val	sSNP		1 ; 5638		5638	no	T in KIV7
666	A	T	(2, 3)	exon 421	Thr28Thr	sSNP	101 ; 152148	1819 ; 430544	104 ; 1194	430544	KIV-2B, KIV3	
666	A	C		exon 421	Thr28Thr	sSNP		237 ; 67876		67876	no	T in KIV3
671	G	A		exon 421	Arg30Lys	nsSNP		1 ; 5857		5857	no	conserved
672	A	G		exon 421	Arg30Arg	sSNP	7 ; 39695	14 ; 50620		50620	KIV1, 6, 7, 9, 10	
674	C	A		exon 421	Thr31Asn	nsSNP		1 ; 22415		22415	no	conserved
674	C	T		exon 421	Thr31Ile	nsSNP		1 ; 13109		13109	no	conserved
675	C	T		exon 421	Thr31Thr	sSNP		34 ; 42211	2 ; 895	42211	KIV5	KIV6-10
677	G	A		exon 421	Cys32Thr	nsSNP		1 ; 27448		27448	no	conserved
678	C	A		exon 421	Cys32X	nonsense	1 ; 4537				no	KIV6-10
679	C	A		exon 421	Gln33Thr	nsSNP		1 ; 21479		21479	no	conserved
679	C	T		exon 421	Gln33X	nonsense		2 ; 13875		13875	no	conserved
683	C	T		exon 421	Ala34Val	nsSNP		3 ; 48941		48941	no	conserved
684	T	C		exon 421	Ala34Ala	sSNP		1 ; 26304		26304	no	conserved

Pos	Ref	Var	Prev. cit.	Locus	Amino acid	Variant type	Discovery set [n, max. cov.]	1000G Exome [n ; max. cov.]	1000G Polaris WGS [n ; max. cov.]	Maximum coverage observed in 1000 Genomes	Variant base is wild type in another kringle	Other base differences present at this position in any other kringle IV (KIV-1; KIV-3 to -10)
686	G	C		exon 421	Trp35Ser	nsSNP	2 ; 21532	2 ; 12681		12681	no	conserved
687	G	C		exon 421	Trp35Cys	nsSNP		1 ; 207		207	no	conserved
688	T	A		exon 421	Ser36Thr	nsSNP		4 ; 27708		27708	no	conserved
690	A	G		exon 421	Ser36Ser	sSNP		1 ; 18771		18771	KIV8	C in KIV6, 7
693	T	C	(2)	exon 421	Ser37Ser	sSNP		1 ; 870		870	KIV10	
694	A	C		exon 421	Met38Leu	nsSNP	1 ; 3610	27 ; 36053		36053	no	conserved
700	C	A		exon 421	Pro40Thr	nsSNP		1 ; 849		849	no	conserved
701	C	T		exon 421	Pro40Leu	nsSNP		1 ; 9333		9333	no	conserved
703	C	A		exon 421	His41Asn	nsSNP		1 ; 735		735	no	conserved
704	A	G		exon 421	His41Arg	nsSNP		4 ; 24474	1 ; 663	24474	no	conserved
705	C	T		exon 421	His41His	sSNP		1 ; 978		978	KIV1, 8	
707	C	T		exon 421	Ser42Leu	nsSNP		35 ; 60296	35 ; 915	60296	no	G in KIV6-10; A in KIV1, 5
707	C	A		exon 421	Ser42X	nonsense		2 ; 681		681	KIV1, 5	
708	G	A		exon 421	Ser42Ser	sSNP		23 ; 85327		85327	KIV1	
708	G	T		exon 421	Ser42Ser	sSNP		1 ; 7196		7196	no	
711	T	G		exon 421	His43Gln	nsSNP		1 ; 29524		29524	no	conserved
712	A	G		exon 421	Ser44Gly	nsSNP		1 ; 31449	1 ; 753	31449	no	C in KIV6-10
715	C	T		exon 421	Arg45Trp	nsSNP		1 ; 47007		47007	no	A KIV1, 6-10
716	G	A		exon 421	Arg45Gln	nsSNP		19 ; 71595		71595	no	conserved
717	G	T		exon 421	Arg45Arg	sSNP	3 ; 38422				no	A in KIV7
719	C	T		exon 421	Thr46Ile	nsSNP		80 ; 41000	5 ; 822	41000	KIV8	
719	C	A		exon 421	Thr46Asn	nsSNP		16 ; 53827	2 ; 1179	53827	no	T in KIV8
721	C	A		exon 421	Pro47Thr	nsSNP		1 ; 745		745	KIV1, 6, 7	
721	C	T		exon 421	Pro47Ser	nsSNP		1 ; 6470		6470	no	conserved
726	A	G	(3)	exon 421	Glu48Glu	sSNP		3 ; 20052		20052	no	conserved
728	A	G		exon 421	Tyr49Cys	nsSNP		45 ; 43823	3 ; 857	43823	no	conserved
730	T	G		exon 421	Tyr50Asp	nsSNP	1 ; 26698	6 ; 21795	1 ; 897	21795	no	conserved
732	C	T		exon 421	Tyr50Tyr	sSNP		6 ; 13730	1 ; 1166	13730	KIV6, 8	

Pos	Ref	Var	Prev. cit.	Locus	Amino acid	Variant type	Discovery set [n, max. cov.]	1000G Exome [n ; max. cov.]	1000G Polaris WGS [n ; max. cov.]	Maximum coverage observed in 1000 Genomes	Variant base is wild type in another kringle	Other base differences present at this position in any other kringle IV (KIV-1; KIV-3 to -10)
732	C	A		exon 421	Tyr50X	nonsense		2 ; 7757		7757	no	T in KIV6, 8
733	C	T		exon 421	Pro51Ser	nsSNP		9 ; 54504	1 ; 871	54504	no	conserved
733	C	A		exon 421	Pro51Thr	nsSNP		1 ; 689		689	no	conserved
734	C	G		exon 421	Pro51Arg	nsSNP		1 ; 24576		24576	no	conserved
734	C	T		exon 421	Pro51Leu	nsSNP		10 ; 28518		28518	no	conserved
735	A	C		exon 421	Pro51Pro	sSNP		1 ; 27637	1 ; 814	27637	no	conserved
740	C	A		exon 421	Ala53Glu	nsSNP		1 ; 651		651	KIV10	G in KIV6, 7
740	C	T		exon 421	Ala53Val	nsSNP		5 ; 11727		11727	no	G in KIV6, 7; T in KIV10
741	G	A		exon 421	splice_site	ssSNP		11 ; 30678		30678	no	conserved
741	G	C		exon 421	splice_site	sSNP		1 ; 28504		28504	no	conserved
4746	G	A		exon 422	Gly54Asp	nsSNP		1 ; 3906		3906	no	conserved
4747	C	T		exon 422	Gly54Gly	sSNP		3 ; 9859		9859	no	conserved
4755	T	C		exon 422	Met57Thr	nsSNP		3 ; 8848		8848	no	G in KIV5-8, A in KIV4
4759	C	G		exon 422	Asn58Lys	nsSNP		1 ; 3238		3238	no	conserved
4766	A	C		exon 422	Arg61Arg	sSNP		1 ; 338		338	KIV4	
4767	G	A		exon 422	Arg61Lys	nsSNP		1 ; 9167		9167	no	conserved
4768	G	T		exon 422	Arg61Ser	nsSNP		1 ; 11363		11363	no	A in KIV4
4770	A	G		exon 422	Asn62Ser	nsSNP		1 ; 2380		2380	no	conserved
4773	C	T		exon 422	Pro63Leu	nsSNP		1 ; 5341		5341	no	conserved
4774	A	T	(2)	exon 422	ProPro	sSNP		61 ; 14236	6 ; 821	14236	no	conserved
4781	G	T		exon 422	Val66Leu	nsSNP		2 ; 7018		7018	no	conserved
4782	T	C		exon 422	Val66Ala	nsSNP		2 ; 10033		10033	no	A in KIV5-8 and 10; G in KIV9
4785	C	T		exon 422	Ala67Val	nsSNP		1 ; 3873		3873	KIV10	
4787	G	T		exon 422	Ala68Ser	nsSNP		1 ; 4367		4367	no	KIV5,7-9; A in KIV6
4788	C	A		exon 422	Ala68Asp	nsSNP		5 ; 6350		6350	KIV9	G in KIV5-7,8,10

Pos	Ref	Var	Prev. cit.	Locus	Amino acid	Variant type	Discovery set [n, max. cov.]	1000G Exome [n ; max. cov.]	1000G Polaris WGS [n ; max. cov.]	Maximum coverage observed in 1000 Genomes	Variant base is wild type in another kringle	Other base differences present at this position in any other kringle IV (KIV-1; KIV-3 to -10)
4788	C	T		exon 422	Ala68Val	nsSNP		1 ; 5771		5771	no	G in KIV5-7,8,10; A in KIV9
4790	C	T		exon 422	Pro69Ser	nsSNP		1 ; 6767		6767	no	conserved
4791	C	A		exon 422	Pro69His	nsSNP		1 ; 7076		7076	no	conserved
4799	T	C		exon 422	Tyr72His	nsSNP		1 ; 8339		8339	no	conserved
4801	T	C	(2)	exon 422	Tyr72Tyr	sSNP		94 ; 62695	8 ; 812	62695	KIV5,8,9	
4803	C	A		exon 422	Thr73Lys	nsSNP		1 ; 10505	1 ; 780	10505	no	conserved
4803	C	T		exon 422	Thr73Met	nsSNP		12 ; 15452		15452	no	conserved
4804	G	C		exon 422	Thr73Thr	sSNP		1 ; 12464		12464	KIV5-8, 10	A in KIV4, 9
4804	G	A		exon 422	Thr73Thr	sSNP		7 ; 465		12464	KIV4, 9	C in KIV5-8, 10
4807	G	C		exon 422	Arg74Ser	nsSNP		2 ; 14228		14228	KIV9	A KIV4
4808	G	A		exon 422	Asp75Asn	nsSNP			1 ; 455	455	no	conserved
4811	C	T		exon 422	Pro76Ser	nsSNP		4 ; 5810		5810	no	conserved
4813	C	T		exon 422	Pro76Pro	sSNP	1 ; 43138	6 ; 12069		12069	no	G in KIV9
4813	C	G		exon 422	Pro76Pro	sSNP		2 ; 13453		13453	KIV9	
4814	G	A		exon 422	Gly77Ser	nsSNP	1 ; 38521	35 ; 18425	1 ; 542	18425	KIV3-7, 10	T in KIV8
4815	G	A		exon 422	Gly77Asp	nsSNP		7 ; 6838		6838	KIV6	
4815	G	T		exon 422	Gly77Val	nsSNP		4 ; 11675		11675	no	A in KIV6
4816	T	G		exon 422	Gly77Gly	sSNP		1 ; 3495		3495	no	C in KIV10
4831	C	G		exon 422	Tyr82X	nonsense		1 ; 8593		8593	no	conserved
4833	G	A		exon 422	Cys83Tyr	nsSNP		1 ; 2765		2765	no	conserved
4835	A	T		exon 422	Asn84Tyr	nsSNP		1 ; 13923		13923	no	conserved
4837	C	A		exon 422	Asn84Lys	nsSNP		2 ; 6698		6698	no	conserved
4837	C	T		exon 422	Asn84Asn	sSNP		1 ; 18650		18650	KIV9	conserved
4840	G	T		exon 422	Leu85Leu	sSNP		1 ; 3900		3900	no	conserved
4842	C	T		exon 422	Thr86Met	nsSNP		19 ; 12029		12029	no	conserved
4843	G	A		exon 422	Thr86Thr	sSNP		1 ; 218		218	KIV3-6, 8, 9	
4844	C	G		exon 422	Gln87Glu	nsSNP		11 ; 13096		13096	no	conserved
4846	A	G		exon 422	Gln87Gln	sSNP		1 ; 4066		4066	no	conserved

Pos	Ref	Var	Prev. cit.	Locus	Amino acid	Variant type	Discovery set [n, max. cov.]	1000G Exome [n ; max. cov.]	1000G Polaris WGS [n ; max. cov.]	Maximum coverage observed in 1000 Genomes	Variant base is wild type in another kringle	Other base differences present at this position in any other kringle IV (KIV-1; KIV-3 to -10)
4848	G	C		exon 422	Cys88Ser	nsSNP		1 ; 4201		4201	no	conserved
4854	A	G		exon 422	Asp90Gly	nsSNP		1 ; 4541		4541	no	T in KIV5-8
4855	C	T		exon 422	Asp90Asp	sSNP		11 ; 18402		18402	KIV4	G in KIV5-8; A in KIV9
4856	G	A	(2)	exon 422	Ala91Thr	nsSNP	1 ; 32712	6 ; 9110		9110	KIV5-10	
4858	A	G		exon 422	Ala91Ala	sSNP		18 ; 20814		20814	KIV7	
4859	G	C		exon 422	Glu92Gln	nsSNP		2 ; 5385	1 ; 802	5385	no	conserved
4859	G	T		exon 422	Glu92X	nonsense		1 ; 2608		2608	no	conserved
4861	A	G		exon 422	Glu92Glu	sSNP		1 ; 240		240	no	conserved
4862	G	A		exon 422	Gly93Arg	nsSNP		2 ; 6736		6736	no	T in KIV4-9
4864	G	A		exon 422	Gly93Gly	sSNP		2 ; 8738		8738	KIV5-7, 9	
4865	A	G		exon 422	Thr94Ala	nsSNP		2 ; 4412		4412	KIV9	
4867	T	G		exon 422	Thr94Thr	sSNP		1 ; 4150		4150	no	
4869	C	G		exon 422	Ala95Gly	nsSNP		5 ; 7477	1 ; 860	7477	no	T in KIV5-10
4870	C	T		exon 422	Ala95Ala	sSNP		3 ; 10892		10892	no	G in KIV10
4871	G	A		exon 422	Val96Ile	nsSNP	1 ; 18741	5 ; 15041		15041	no	C KIV5-9; T in KIV4
4871	G	C		exon 422	Val96Leu	nsSNP		1 ; 3015		3015	KIV5-9	T in KIV4
4873	C	T		exon 422	Val96Val	sSNP		1 ; 7816		7816	KIV5, 6	A in KIV9
4874	G	A		exon 422	Ala97Thr	nsSNP		3 ; 5440		5440	KIV7, 8	
4875	C	T		exon 422	Ala97Val	nsSNP		2 ; 9784		9784	KIV4	A in KIV9
4876	G	T		exon 422	Ala97Ala	sSNP		16 ; 18944	2 ; 897	18944	KIV10	C in KIV4; A in KIV5,7,8
4876	G	A		exon 422	Ala97Ala	sSNP		1 ; 224		224	KIV5,7,8	
4877	C	T		exon 422	Pro98Ser	nsSNP		2 ; 6063		6063	no	A in KIV5-9
4879	T	C		exon 422	Pro98Pro	sSNP	1 ; 15974	NA	NA	NA	no	G in KIV6
4881	C	T		exon 422	Pro99Leu	nsSNP		4 ; 18803		18803	KIV5	
4881	C	A		exon 422	Pro99Gln	nsSNP		3 ; 9917	1 ; 574	9917	no	

Pos	Ref	Var	Prev. cit.	Locus	Amino acid	Variant type	Discovery set [n, max. cov.]	1000G Exome [n ; max. cov.]	1000G Polaris WGS [n ; max. cov.]	Maximum coverage observed in 1000 Genomes	Variant base is wild type in another kringle	Other base differences present at this position in any other kringle IV (KIV-1; KIV-3 to -10)
4882	G	A		exon 422	Pro99Pro	sSNP		154 ; 79978	13 ; 916	79978	KIV3	C in KIV5-9
4884	C	T		exon 422	Thr100Ile	nsSNP		1 ; 9644		9644	no	A in KIV4
4885	T	G		exon 422	Thr100Thr	sSNP	1 ; 16899	1 ; 7416	1 ; 728	7416	KIV5-7	A in KIV8
4890	C	A		exon 422	Thr102Asn	nsSNP		4 ; 7470		7470	no	T in KIV4-7, 9,10
4890	C	T		exon 422	Thr102Ile	nsSNP		1 ; 140		140	KIV4-7, 9,10	
4891	C	A		exon 422	Thr102Thr	sSNP		1 ; 3964		3964	no	T in KIV4,6,9
4891	C	T		exon 422	Thr102Thr	sSNP		1 ; 3884		3964	KIV4,6,9	
4893	C	T		exon 422	Pro103Leu	nsSNP	2 ; 5273	41 ; 16703	2 ; 889	16703	KIV4	A in KIV10
4893	C	A		exon 422	Pro103Gln	nsSNP		1 ; 288		288	KIV10	T in KIV4
4893	C	G		exon 422	Pro103Arg	nsSNP		1 ; 9655		9655	no	
4894	G	A		exon 422	Pro103Pro	sSNP		3 ; 24118		24118	KIV5,7,9	T in KIV6
4894	G	C		exon 422	Pro103Pro	sSNP		1 ; 3893		3893	no	A in KIV5,7,9; T in KIV6
4895	G	A		exon 422	Val104Ile	nsSNP		1 ; 2788		2788	KIV3	
4898	C	T		exon 422	Pro105Ser	nsSNP		10 ; 75762	1 ; 565	75762	no	conserved
4898	C	G		exon 422	Pro105Ala	nsSNP		1 ; 168		168	no	conserved
4899	C	G		exon 422	Pro105Arg	nsSNP		1 ; 16966		16966	no	A in KIV6
4900	A	C		exon 422	Pro105Pro	sSNP		1 ; 138		138	no	conserved
4903	C	A		exon 422	Ser106Arg	nsSNP		5 ; 12905		12905	no	conserved
4907	G	T		exon 422	Glu108X	nonsense		2 ; 10694		10694	no	conserved
4908	A	G		exon 422	Glu108Gly	nsSNP	3 ; 86069	7 ; 6909	2 ; 689	6909	KIV10	
4910	G	T		exon 422	Ala109Ser	nsSNP	1 ; 40296	1 ; 252		252	no	C in KIV7, 10
4911	C	T		exon 422	Ala109Val	nsSNP		3 ; 10585		10585	KIV7	
4911	C	A		exon 422	Ala109Asp	nsSNP		8 ; 9519		9519	no	T in KIV7
4912	T	C		exon 422	Ala109Ala	sSNP	2 ; 18093	NA	NA	NA	no	conserved
4913	C	T		exon 422	Pro110Ser	nsSNP		4 ; 21577		21577	KIV4,5	conserved
4914	C	T		exon 422	Pro110Leu	nsSNP		5 ; 21791		21791	KIV4	A in KIV9
4916	T	C		exon 422	Ser111Pro	nsSNP		1 ; 7967		7967	no	conserved
4917	C	T		exon 422	Ser111Phe	nsSNP		1 ; 6627		6627	KIV4	

Pos	Ref	Var	Prev. cit.	Locus	Amino acid	Variant type	Discovery set [n, max. cov.]	1000G Exome [n ; max. cov.]	1000G Polaris WGS [n ; max. cov.]	Maximum coverage observed in 1000 Genomes	Variant base is wild type in another kringle	Other base differences present at this position in any other kringle IV (KIV-1; KIV-3 to -10)
4918	C	T		exon 422	Ser111Ser	sSNP		2 ; 5462	1 ; 829	5462	KIV3-10	
4919	G	T		exon 422	Glu112X	nonsense		2 ; 5324		5324	no	conserved
4919	G	C		exon 422	Glu112Gln	nsSNP		1 ; 4787		4787	no	conserved
4921	A	G		exon 422	Glu112Glu	sSNP		1 ; 13488		13488	no	conserved
4925	G	A		exon 422	Ala114Thr	ssSNP	32 ; 165660	172 ; 157429	11 ; 744	157429	no	conserved

Supplemental Table S10: Positions showing an average variant level >0.5

Pos	Ref	Variant	Variant level (mean)	Variant level (SD)	Number of samples	Caused by intra-reference differences
35	**C**	G	0.63	0.24	71	no
1356	**T**	C	0.60	0.23	71	no
1942	**A**	T	0.98	0.02	122	yes; only the reference KIV-2 nr 6 presents this wild type base
2409	**A**	T	0.68	0.20	122	yes; only the reference KIV-2 nr 6 and the KIV-2B of the reference present this wild type base
2449	**T**	C	0.96	0.04	122	yes; only the reference KIV-2 nr 6 presents this wild type base
2687	**A**	G	0.74	0.16	123	yes; two KIV-2 in the reference sequence harbor this wild type base and four the mutant base
2688	**T**	G	0.75	0.16	123	yes; two KIV-2 in the reference sequence harbor this wild type base and four the mutant base
2705	**A**	G	0.60	0.17	123	yes; three KIV-2 in the reference sequence harbor the wild type base and three the mutant base
3061	**G**	A	0.60	0.24	123	yes; two KIV-2 in the reference sequence (nr 2 and 4) present the mutant base as wild type base
3103	**G**	C	0.62	0.25	71	no
4071	**A**	C	0.61	0.25	71	no
4072	**C**	A	0.61	0.25	71	no
4265	**C**	T	0.64	0.21	123	yes; three KIV-2 in the reference sequence harbor the wild type base and three the mutant base
4358	**A**	G	0.62	0.25	71	no
4576	**C**	G	0.96	0.05	120	yes; only the reference KIV-2 nr 6 presents this wild type base

Supplemental Table S11: Number of carriers for each exonic mutation in each population of the 1000G exome dataset.

Due to the high coverage of all variants (Supplemental Table S7) no formal coverage limit was set. For better representation of patterns, filled cells are highlighted in green. Previous citations for each variant can be found in Supplemental Table S7.

		AFR							AMR				EAS					EUR					SAS				
variant	amino acid	ACB	ASW	ESN	GWD	LWK	MSL	YRI	CLM	MXL	PEL	PUR	CDX	CHB	CHS	JPT	KHV	CEU	FIN	GBR	IBS	TSI	BEB	GIH	ITU	PJL	STU
579 A/C	splice site													1	2												
583 C/A	Pro1Thr												1														
584 C/T	Pro1Leu	1	1						1			3	1		1		1	3	1	9	2	1	1	2	3	3	1
585 G/A	Pro1Pro				1									1	1												
585 G/T	Pro1Pro					1																					
587 C/T	Thr2Ile											1									4						
589 G/A	Glu3Lys										2																
591 G/T	Glu3Asp	2	5	5	13	4	7	5	1			2															
594 A/G	Gln4Gln	71	48	105	121	44	94	83	86	60	77	101	93	81	111	88	102	72	91	78	88	73	85	92	102	95	101
597 G/T	Arg5Ser		2																								
598 C/T	Pro6Ser						2						1		1												
602 G/A	Gly7Glu	7	3	13	7	3	10	11																			
605 T/G	Val8Gly														1												
606 G/A	Val8Val																						1			3	1
606 G/T	Val8Val												2														
607 C/G	Gln9Glu									1																	
607 C/T	Gln9X						1																				
608 A/G	Gln9Arg																						1	1			1
609 G/A	Gln9Gln																								1	1	
611 A/C	Glu10Ala														2												

		AFR							AMR				EAS					EUR					SAS				
variant	amino acid	ACB	ASW	ESN	GWD	LWK	MSL	YRI	CLM	MXL	PEL	PUR	CDX	CHB	CHS	JPT	KHV	CEU	FIN	GBR	IBS	TSI	BEB	GIH	ITU	PJL	STU
611 A/G	Glu10Gly																		1								
615 C/T	Cys11Cys																						1	1			
618 C/A	Tyr12X																1										
619 C/A	His13Asn												2														
619 C/T	His13Tyr																								1	1	2
620 A/G	His13Arg																										1
621 T/C	His13His	72	46	101	120	49	86	87	88	61	82	101	92	82	112	93	106	77	92	82	97	84	85	95	99	95	100
622 G/A	Gly14Ser	1		1														1									
623 G/T	Gly14Val		1	1								1											1				
624 T/C	Gly14Gly																			1							
624 T/G	Gly14Gly																						1	1	2		1
625 A/G	Asn15Asp	1						1																			
626 A/G	Asn15Ser																								1		
627 T/C	Asn15Asn													1													
629 G/A	Gly16Glu												1														
635 G/C	Ser18Thr																	1									
635 G/T	Ser18Ile																				1						
640 C/A	Arg20Arg								1	1																	
640 C/T	Arg20X								1			2		1				5	3	2	2	2	3	1	1	3	1
641 G/A	Arg20Gln													1		1		1									
641 G/C	Arg20Pro																		1								
642 A/C	Arg20Arg																						1				
643 G/C	Gly21Arg																						2	1			1
644 G/A	Gly21Asp	1		3				1				1															
644 G/T	Gly21Val														1												
645 C/G	Gly21Gly																			2							
645 C/T	Gly21Gly												1														

		AFR							AMR				EAS					EUR					SAS				
**variant**	**amino acid**	**ACB**	**ASW**	**ESN**	**GWD**	**LWK**	**MSL**	**YRI**	**CLM**	**MXL**	**PEL**	**PUR**	**CDX**	**CHB**	**CHS**	**JPT**	**KHV**	**CEU**	**FIN**	**GBR**	**IBS**	**TSI**	**BEB**	**GIH**	**ITU**	**PJL**	**STU**
647 C/T	Thr22Ile	1												1													
649 T/C	Tyr23His																								1		
652 T/A	Ser24Thr														1												
653 C/A	Ser24Tyr	3	5	5	12	3	5	5	1			2															
656 C/A	Thr25Asn											1															
657 C/A	Thr25Thr									1		1						1	5		2	3					
657 C/T	Thr25Thr	2	2	1	1	3	4	4	2			3								1		2		1			1
661 G/C	Val27Leu																								2		1
663 C/A	Val27Val																						1				
666 A/C	Thr28Thr	23	8	10	28	5	15	7	4	2	2	4	5	10	6	10	8	11	6	9	6	13	4	8	16	8	9
666 A/T	Thr28Thr	47	37	73	83	49	60	62	79	57	76	87	83	75	100	81	91	67	81	68	90	73	75	86	81	81	81
671 G/A	Arg30Lys																						1				
672 A/G	Arg30Arg								2	1		3						1	2		1		1		1	1	1
674 C/A	Thr31Asn	1																									
674 C/T	Thr31Ile																										1
675 C/T	Thr31Thr	2	5	3	9	7	3	4	1			2															
677 G/A	Cys32Thr									1																	
679 C/A	Gln33Thr																				1						
679 C/T	Gln33X															1										1	
683 C/T	Ala34Val											2														1	
684 T/C	Ala34Ala																								1		
686 G/C	Trp35Ser		1																		1						
687 G/C	Trp35Cys																	1									
688 T/A	Ser36Thr																						1	1	1	1	
690 A/G	Ser36Ser						1																				
693 T/C	Ser37Ser																		1								
694 A/C	Met38Leu								1	2		2						2		1	2	2	3	2	2	3	5

		AFR							AMR				EAS					EUR					SAS				
variant	amino acid	ACB	ASW	ESN	GWD	LWK	MSL	YRI	CLM	MXL	PEL	PUR	CDX	CHB	CHS	JPT	KHV	CEU	FIN	GBR	IBS	TSI	BEB	GIH	ITU	PJL	STU
700 C/A	Pro40Thr											1															
701 C/T	Pro40Leu																										1
703 C/A	His41Asn													1													
704 A/G	His41Arg		1		4																						
705 C/T	His41His															1											
707 C/A	Ser42X												1							1							
707 C/T	Ser42Leu	3	5	3	8	8	3	2	1			2				1									1		
708 G/A	Ser42Ser		1						2		8	5		1	1				1	1					1	2	
708 G/T	Ser42Ser			1																							
711 T/G	His43Gln																							1			
712 A/G	Ser44Gly			2																							
715 C/T	Arg45Trp															1											
716 G/A	Arg45Gln												3	2	3	6	1						3	1			
719 C/A	Thr46Asn	1	1															3	1	8	1	2		1			
719 C/T	Thr46Ile	9	9	23	9	12	8	14				1															
721 C/A	Pro47Thr											1															
721 C/T	Pro47Ser			1																							
726 A/G	Glu48Glu													1													2
728 A/G	Tyr49Cys	4	5	5	13	7	6	5	1			2															
730 T/G	Tyr50Asp											1							4		2						
732 C/A	Tyr50X													2													
732 C/T	Tyr50Tyr				1		2								2						1		1				
733 C/A	Pro51Thr											1															
733 C/T	Pro51Ser			2		1		1						1	3	1								1			
734 C/G	Pro51Arg				1																						
734 C/T	Pro51Leu		1	1	4			3																			1
735 A/C	Pro51Pro				2																						

		AFR							AMR				EAS					EUR					SAS				
**variant**	**amino acid**	**ACB**	**ASW**	**ESN**	**GWD**	**LWK**	**MSL**	**YRI**	**CLM**	**MXL**	**PEL**	**PUR**	**CDX**	**CHB**	**CHS**	**JPT**	**KHV**	**CEU**	**FIN**	**GBR**	**IBS**	**TSI**	**BEB**	**GIH**	**ITU**	**PJL**	**STU**
740 C/A	Ala53Glu											1															
740 C/T	Ala53Val		1		3																				1		
741 G/A	splice site					1	1			1		1		1				2	1		1	1					1
741 G/C	splice site						1																				
4746 G/A	Gly54Asp				1																						
4747 C/T	Gly54Gly																							1		2	
4755 T/C	Met57Thr												1			1	1										
4759 C/G	Asn58Lys																						1				
4766 A/C	Arg61Arg															1											
4767 G/A	Arg61Lys													1													
4768 G/T	Arg61Ser																1										
4770 A/G	Asn62Ser		1																								
4773 C/T	Pro63Leu		1																								
4774 A/T	Pro63Pro	9	5	14	1	13	11	13	1																		
4781 G/T	Val66Leu		1																		1						
4782 T/C	Val66Ala																					2					
4785 C/T	Ala67Val									1																	
4787 G/T	Ala68Ser					1																					
4788 C/A	Ala68Asp															5											
4788 C/T	Ala68Val																								1		
4790 C/T	Pro69Ser			1																							
4791 C/A	Pro69His																	1									
4799 T/C	Tyr72His				1																						
4801 T/C	Tyr72Tyr	8	5	27	21	11	15	13	1			1															
4803 C/A	Thr73Lys				2																						
4803 C/T	Thr73Met			1							1					4								4	1	1	
4804 G/A	Thr73Thr			2																				5			

		AFR							AMR				EAS					EUR					SAS				
variant	amino acid	ACB	ASW	ESN	GWD	LWK	MSL	YRI	CLM	MXL	PEL	PUR	CDX	CHB	CHS	JPT	KHV	CEU	FIN	GBR	IBS	TSI	BEB	GIH	ITU	PJL	STU
4804 G/C	Thr73Thr																			1							
4807 G/C	Arg74Ser										2																
4808 G/A	Asp75Asn																				1						
4811 C/T	Pro76Ser							2					1		1												
4813 C/G	Pro76Pro																							1		1	
4813 C/T	Pro76Pro							1		1							1		3								
4814 G/A	Gly77Ser		2						3			4						4	4	4	3	2		2	3	5	
4815 G/A	Gly77Asp	1	1			4		1																			
4815 G/T	Gly77Val						1																1		1	1	
4816 T/G	Gly77Gly									1																	
4831 C/G	Tyr82X				1																						
4833 G/A	Cys83Tyr			1																							
4835 A/T	Asn84Tyr																							1			
4837 C/A	Asn84Lys																					2					
4837 C/T	Asn84Asn																							1			
4840 G/T	Leu85Leu																						1				
4842 C/T	Thr86Met	2	1	1	6		4	1	1							1		1				1					
4843 G/A	Thr86Thr																1										
4844 C/G	Gln87Glu	1	1	2	6		1																				
4846 A/G	Gln87Gln									1																	
4848 G/C	Cys88Ser									1																	
4854 A/G	Asp90Gly									1																	
4855 C/T	Asp90Asp	2	1		5							2								1							
4856 G/A	Ala91Thr	1	2			3																					
4858 A/G	Ala91Ala	1		5		1	2	6	1		1																1
4859 G/C	Glu92Gln																			2						1	
4859 G/T	Glu92X																									1	

		AFR							AMR				EAS					EUR					SAS				
variant	amino acid	ACB	ASW	ESN	GWD	LWK	MSL	YRI	CLM	MXL	PEL	PUR	CDX	CHB	CHS	JPT	KHV	CEU	FIN	GBR	IBS	TSI	BEB	GIH	ITU	PJL	STU
4861 A/G	Glu92Glu																					1					
4862 G/A	Gly93Arg													1		1											
4864 G/A	Gly93Gly																								1		1
4865 A/G	Thr94Ala								1	1																	
4867 T/G	Thr94Thr										1																
4869 C/G	Ala95Gly		1	2		3																					
4870 C/T	Ala95Ala																						2		1		
4871 G/A	Val96Ile																2	1				2					
4871 G/C	Val96Leu									1																	
4873 C/T	Val96Val								1																		
4874 G/A	Ala97Thr							1																1		1	
4875 C/T	Ala97Val	1								1																	
4876 G/A	Ala97Ala												1														
4876 G/T	Ala97Ala		1		5	3	3	3			1	2															
4877 C/T	Pro98Ser								1														1				
4881 C/A	Pro99Gln	1		2								1															
4881 C/T	Pro99Leu										2						1	1									
4882 G/A	Pro99Pro	16	12	42	25	23	24	22	1			1													1		
4884 C/T	Thr100Ile																		1								
4885 T/G	Thr100Thr																		2								
4890 C/A	Thr102Asn							3														1					
4890 C/T	Thr102Ile																			1							
4891 C/A	Thr102Thr														1												
4891 C/T	Thr102Thr																						1				
4893 C/A	Pro103Gln										1																
4893 C/G	Pro103Arg				1																						
4893 C/T	Pro103Leu				3				1	1	1							1	2	2	2	2	3	3	5	10	7

		AFR							AMR				EAS					EUR					SAS				
variant	amino acid	ACB	ASW	ESN	GWD	LWK	MSL	YRI	CLM	MXL	PEL	PUR	CDX	CHB	CHS	JPT	KHV	CEU	FIN	GBR	IBS	TSI	BEB	GIH	ITU	PJL	STU
4894 G/A	Pro103Pro			1							1		1														
4894 G/C	Pro103Pro									1																	
4895 G/A	Val104Ile											1															
4898 C/G	Pro105Ala										1																
4898 C/T	Pro105Ser			1	4	1		3													2						
4899 C/G	Pro105Arg										1																
4900 A/C	Pro105Pro	1																									
4903 C/A	Ser106Arg				1	1							1			1	1										
4907 G/T	Glu108X																								1	1	
4908 A/G	Glu108Gly									1	1							1	3	1	1	1					
4910 G/T	Ala109Ser										1																
4911 C/A	Ala109Asp																						1		2	4	1
4911 C/T	Ala109Val	1																								2	
4913 C/T	Pro110Ser	1									1	1													1		
4914 C/T	Pro110Leu	1							1		1	1													1		
4916 T/C	Ser111Pro																										1
4917 C/T	Ser111Phe																							1			
4918 C/T	Ser111Ser														2							1					
4919 G/C	Glu112Gln						1																				
4919 G/T	Glu112X				1		1																				
4921 A/G	Glu112Glu																							1			
4925 G/A	Ala114Thr	3	2	1	6		1	2	13	13	5	7				2		12	9	17	12	16	6	8	17	12	19

Supplemental Table S12: Aggregated frequency of missense and nonsense variants (nsSNPs, nonsense and splice site variants) in the single populations.

Since coverage differs between the two exons, the numbers are split by exons. Variant frequency is given as variant density instead of MAF to account for the fact, that some individuals carried more than one missense variant. B) Aggregated frequency of nonsense variants. For overall carrier frequency calculation, sample numbers for exon 1 were taken as reference

	AFR	AMR	EAS	EUR	SAS
total samples >780X, exon 1 [n]	545	279	324	266	468
total samples >780X, exon 2 [n]	521	252	147	191	460
**A) Aggregated frequency of missense variants**					
n variants exon 1	307	28	35	51	71
n variants exon 2	90	68	18	72	132
Variant density exon 1 [1]	0.0035	0.0007	0.0007	0.0012	0.0010
Variant density exon 2 [1]	0.0010	0.0015	0.0006	0.0021	0.0016
Variant density overall [1]	0.0022	0.0011	0.0007	0.0016	0.0013
p for equal proportions between exon 1 and 2	1.16E-31	3.12E-04	1	3.41E-03	8.65E-04
**B) Aggregated frequency of nonsense variants**					
carrier nonsense [n]	4	3	3	9	13
n carrier nonsense without p.(Arg20Ter) [n]	4	0	3	0	4
Frequency carriers nonsense variants [%]	0.007	0.011	0.009	0.034	0.026

[1] n variants detected/ total exon bases sequenced

Supplemental Table S13: KIV-2B levels for each sample of the discovery set carrying KIV-2B variants.

Based on the estimate of total KIV-2 copies in the genome, the estimated number of KIV-2B repeats is given for orientation. Positions are named relative to our reference sequence, with positions 594, 621 and 666 being the canonical KIV-2B positions (positions 14, 41 and 86 in the exon 421, respectively).

	Pos. 594		Pos. 621		Pos. 666		
sample nr.	carrier KIV-2 [n], estimated	variation level NGS	carrier KIV-2 [n], estimated	variation level NGS	carrier KIV-2 [n], estimated	variation level NGS	Estimated number of KIV-2C
1	1.36	0.04	1.38	0.04	1.35	0.04	0.0
2	2.04	0.06	2.10	0.06	2.09	0.06	0.0
3	2.66	0.11	2.67	0.11	2.73	0.12	0.1
4	8.29	0.19	8.34	0.19	7.06	0.16	1.3
5	1.27	0.03	1.29	0.03	1.21	0.03	0.1
6	1.48	0.04	1.49	0.04	1.40	0.03	0.1
7	1.45	0.03	1.48	0.03	1.41	0.03	0.1
8	16.23	0.30	16.12	0.30	13.46	0.25	2.7
9	1.31	0.04	1.31	0.04	1.32	0.04	0.0
10	1.10	0.03	1.16	0.03	1.16	0.03	0.0
11	2.51	0.07	2.55	0.07	2.53	0.07	0.0
12	9.72	0.20	9.83	0.21	8.74	0.18	1.1
13	5.60	0.13	5.83	0.13	5.99	0.14	0.2
14	1.27	0.03	1.41	0.04	1.15	0.03	0.3
15	7.50	0.13	7.68	0.14	6.50	0.12	1.2
16	2.82	0.05	2.95	0.05	3.00	0.05	0.1
17	9.36	0.18	9.48	0.19	8.13	0.16	1.4

	Pos. 594		Pos. 621		Pos. 666		
sample nr.	carrier KIV-2 [n], estimated	variation level NGS	carrier KIV-2 [n], estimated	variation level NGS	carrier KIV-2 [n], estimated	variation level NGS	Estimated number of KIV-2C
18	8.12	0.17	8.45	0.18	7.70	0.16	0.8
19	7.14	0.13	7.26	0.14	6.27	0.12	1.0
20	1.59	0.05	1.61	0.05	1.60	0.05	0.0
21	3.06	0.08	3.24	0.08	3.25	0.08	0.0
22	2.98	0.07	3.07	0.07	3.07	0.07	0.0
23	3.60	0.06	3.60	0.06	3.54	0.06	0.1
24	1.38	0.03	1.46	0.03	1.44	0.03	0.0
25	2.35	0.07	2.43	0.07	2.51	0.07	0.1
26	1.38	0.03	1.42	0.03	1.40	0.03	0.0
27	9.28	0.21	9.34	0.21	7.98	0.18	1.4
28	1.10	0.03	1.08	0.03	1.00	0.03	0.1
29	3.05	0.06	3.22	0.06	3.20	0.06	0.0
30	8.26	0.18	8.31	0.18	7.39	0.16	0.9
31	2.71	0.07	2.72	0.07	2.75	0.07	0.0
32	9.90	0.22	10.08	0.22	8.80	0.19	1.3
33	3.19	0.09	3.25	0.09	3.23	0.09	0.0
34	2.89	0.07	2.88	0.07	3.00	0.07	0.1
35	1.61	0.04	1.52	0.03	1.49	0.03	0.0
36	1.34	0.04	1.27	0.04	1.29	0.04	0.0
37	1.74	0.05	1.66	0.04	1.58	0.04	0.1
38	1.56	0.04	1.61	0.04	1.47	0.04	0.1
39	8.28	0.21	8.45	0.21	6.36	0.16	2.1
40	7.22	0.15	7.31	0.16	5.93	0.13	1.4
41	1.52	0.03	1.51	0.03	1.49	0.03	0.0

	Pos. 594		Pos. 621		Pos. 666		
sample nr.	carrier KIV-2 [n], estimated	variation level NGS	carrier KIV-2 [n], estimated	variation level NGS	carrier KIV-2 [n], estimated	variation level NGS	Estimated number of KIV-2C
42	2.01	0.05	2.02	0.05	2.04	0.05	0.0
43	7.68	0.22	7.91	0.22	6.58	0.18	1.3
44	2.74	0.05	2.76	0.05	2.79	0.05	0.0
45	1.32	0.03	1.38	0.03	1.32	0.03	0.1
46	11.45	0.22	11.56	0.22	10.37	0.20	1.2
47	11.40	0.18	11.60	0.18	10.10	0.16	1.5
48	5.25	0.15	5.41	0.15	5.44	0.15	0.0
49	9.23	0.25	9.33	0.25	8.09	0.22	1.2
50	2.69	0.06	2.75	0.06	2.71	0.06	0.0
51	1.32	0.03	1.38	0.03	1.44	0.03	0.1
52	1.42	0.05	1.52	0.05	1.60	0.05	0.1
53	9.73	0.18	9.86	0.19	8.77	0.17	1.1
54	4.54	0.08	4.74	0.08	4.19	0.07	0.5
55	1.25	0.03	1.30	0.03	1.28	0.03	0.0
56	1.76	0.05	1.77	0.05	1.15	0.03	0.6
57	4.74	0.11	5.01	0.11	4.50	0.10	0.5
58	0.75	0.02	0.78	0.02	0.80	0.03	0.0
59	7.62	0.15	7.87	0.15	6.89	0.13	1.0
60	1.72	0.05	1.80	0.05	1.78	0.05	0.0
61	1.26	0.03	1.21	0.03	1.17	0.03	0.0
62	5.72	0.10	5.25	0.09	5.08	0.08	0.2
63	0.63	0.02	0.66	0.02	0.71	0.02	0.0
64	1.59	0.07	1.63	0.07	1.67	0.07	0.0
65	1.74	0.05	1.88	0.05	1.91	0.05	0.0

	Pos. 594		Pos. 621		Pos. 666		
sample nr.	carrier KIV-2 [n], estimated	variation level NGS	carrier KIV-2 [n], estimated	variation level NGS	carrier KIV-2 [n], estimated	variation level NGS	Estimated number of KIV-2C
66	0.89	0.02	0.93	0.02	0.94	0.02	0.0
67	0.79	0.02	0.86	0.02	0.87	0.02	0.0
68	4.41	0.09	4.56	0.10	4.11	0.09	0.5
69	1.36	0.04	1.42	0.04	1.41	0.04	0.0
70	1.32	0.03	1.25	0.03	1.12	0.02	0.1
71	1.05	0.02	1.09	0.03	1.13	0.03	0.0
72	1.23	0.04	1.15	0.04	1.18	0.04	0.0
73	0.94	0.02	0.99	0.03	1.00	0.03	0.0
74	2.59	0.05	2.72	0.06	2.38	0.05	0.3
75	1.31	0.03	1.38	0.03	1.43	0.03	0.1
76	3.86	0.09	4.04	0.10	3.69	0.09	0.4
77	0.65	0.02	0.69	0.02	0.69	0.02	0.0
78	3.18	0.09	3.23	0.09	2.77	0.08	0.5
79	0.61	0.01	0.64	0.01	0.66	0.01	0.0
80	1.17	0.03	1.13	0.03	1.16	0.03	0.0
81	3.60	0.08	3.53	0.08	3.32	0.08	0.2
82	0.51	0.01	0.54	0.01	0.54	0.01	0.0
83	1.27	0.02	1.31	0.02	1.36	0.02	0.0
84	6.61	0.15	6.50	0.14	6.26	0.14	0.2
85	1.08	0.02	1.19	0.03	1.26	0.03	0.1
86	2.80	0.08	2.83	0.08	2.72	0.07	0.1
87	1.20	0.04	1.28	0.04	1.32	0.04	0.0
88	0.99	0.02	1.06	0.02	1.03	0.02	0.0
89	4.83	0.08	4.97	0.09	4.60	0.08	0.4

	Pos. 594		Pos. 621		Pos. 666		
sample nr.	carrier KIV-2 [n], estimated	variation level NGS	carrier KIV-2 [n], estimated	variation level NGS	carrier KIV-2 [n], estimated	variation level NGS	Estimated number of KIV-2C
90	0.63	0.01	0.68	0.02	0.68	0.02	0.0
91	4.22	0.10	4.22	0.10	3.63	0.09	0.6
92	3.30	0.09	3.39	0.09	2.90	0.08	0.5
93	0.66	0.02	0.69	0.02	0.67	0.02	0.0
94	5.88	0.18	5.93	0.18	5.10	0.15	0.8
95	11.97	0.24	11.87	0.24	10.02	0.20	1.8
96	1.59	0.06	1.56	0.06	1.48	0.06	0.1
97	1.37	0.03	1.39	0.03	1.39	0.03	0.0
98	0.63	0.02	0.66	0.02	0.72	0.02	0.1
99	0.61	0.01	0.64	0.01	0.63	0.01	0.0
100	1.53	0.04	1.57	0.04	1.59	0.04	0.0

Supplemental Table S14: Levels of the canonical KIV-2B variants in the 1000G WGS Polaris dataset

Super population	Population	Sample ID	pos. 594	pos. 621	pos. 666	Mean
**AFR**	**ESN**	HG02973	0.040	0.036	0.030	0.035
		HG02974	0.014	0.014	0.007	0.012
		HG03099	0.041	0.035	0.021	0.032
		HG03100	0.088	0.088	0.094	0.090
		HG03120	0.074	0.080	0.072	0.075
		HG03121	0.009	0.013	0.000	0.007
		HG03129	0.029	0.014	0.000	0.014
		HG03130	0.046	0.038	0.000	0.028
		HG03168	0.031	0.038	0.029	0.033
		HG03169	0.060	0.060	0.050	0.057
		HG03300	0.015	0.011	0.000	0.009
		HG03301	0.016	0.015	0.000	0.010
		HG03306	0.017	0.018	0.000	0.012
		HG03307	0.056	0.049	0.040	0.048
		HG03520	0.036	0.036	0.029	0.034
		HG03521	0.057	0.052	0.053	0.054
	**ESN Mean**		**0.039**	**0.037**	**0.027**	**0.034**
	**GWD**	HG02561	0.229	0.228	0.231	0.229
		HG02562	0.043	0.036	0.040	0.040
		HG02620	0.021	0.016	0.000	0.012
		HG02621	0.051	0.047	0.035	0.044
		HG02721	0.022	0.019	0.000	0.014
		HG02722	0.116	0.109	0.113	0.112
		HG02768	0.116	0.124	0.120	0.120
		HG02769	0.050	0.045	0.021	0.039
		HG02771	0.013	0.011	0.000	0.008
		HG02772	0.219	0.221	0.224	0.221
		HG02851	0.110	0.122	0.090	0.107
		HG02852	0.014	0.017	0.000	0.010
		HG02982	0.007	0.004	0.002	0.004
		HG02983	0.058	0.065	0.052	0.059
		HG03039	0.050	0.054	0.036	0.047
		HG03040	0.008	0.012	0.000	0.007
		HG03246	0.006	0.006	0.005	0.006
		HG03247	0.043	0.041	0.029	0.037
	**GWD Mean**		**0.065**	**0.065**	**0.055**	**0.062**
	**MSL**	HG03063	0.034	0.041	0.033	0.036
		HG03064	0.033	0.035	0.024	0.030
		HG03069	0.016	0.016	0.000	0.011
		HG03096	0.021	0.020	0.007	0.016
		HG03097	0.021	0.021	0.000	0.014
		HG03445	0.052	0.057	0.051	0.053
		HG03451	0.012	0.014	0.000	0.009

Super population	Population	Sample ID	pos. 594	pos. 621	pos. 666	Mean
		HG03452	0.037	0.041	0.033	0.037
		HG03484	0.039	0.039	0.030	0.036
		HG03485	0.020	0.011	0.000	0.011
		HG03574	0.038	0.041	0.032	0.037
		HG03575	0.029	0.034	0.019	0.028
		HG03577	0.013	0.011	0.000	0.008
		HG03578	0.060	0.057	0.042	0.053
		HG03582	0.081	0.085	0.093	0.086
		HG03583	0.010	0.013	0.003	0.009
	**MSL Mean**		**0.032**	**0.033**	**0.023**	**0.030**
**EAS**	**CDX**	HG00844	0.279	0.260	0.192	0.244
		HG00881	0.178	0.175	0.157	0.170
		HG00982	0.015	0.014	0.000	0.010
		HG02356	0.015	0.014	0.000	0.010
		HG02372	0.143	0.138	0.086	0.122
		HG02373	0.307	0.292	0.276	0.292
		HG02374	0.234	0.234	0.231	0.233
		HG02375	0.131	0.139	0.107	0.126
		HG02381	0.292	0.288	0.257	0.279
		HG02383	0.282	0.284	0.160	0.242
		HG02384	0.012	0.016	0.000	0.009
		HG02385	0.018	0.020	0.000	0.013
		HG02386	0.118	0.117	0.086	0.107
		HG02388	0.153	0.128	0.099	0.127
		HG02391	0.229	0.222	0.188	0.213
		HG02409	0.299	0.293	0.259	0.284
	**CDX Mean**		**0.169**	**0.165**	**0.131**	**0.155**
	**CHS**	HG00448	0.128	0.115	0.103	0.116
		HG00449	0.150	0.145	0.137	0.144
		HG00583	0.085	0.074	0.071	0.077
		HG00584	0.221	0.234	0.207	0.220
		HG00595	0.020	0.015	0.000	0.011
		HG00596	0.181	0.196	0.186	0.188
		HG00610	0.019	0.018	0.000	0.012
		HG00611	0.012	0.013	0.000	0.008
		HG00613	0.297	0.280	0.253	0.277
		HG00614	0.025	0.022	0.000	0.015
		HG00650	0.011	0.012	0.000	0.008
		HG00651	0.255	0.236	0.215	0.235
		HG00656	0.157	0.149	0.136	0.147
		HG00657	0.216	0.188	0.172	0.192
		HG00662	0.231	0.217	0.173	0.207
		HG00663	0.176	0.183	0.095	0.151
		HG00683	0.034	0.035	0.034	0.034
		HG00684	0.209	0.205	0.158	0.191

Super population	Population	Sample ID	pos. 594	pos. 621	pos. 666	Mean
	**CHS Mean**		**0.135**	**0.130**	**0.108**	**0.124**
	**KHV**	HG02016	0.285	0.275	0.208	0.256
		HG02017	0.010	0.010	0.000	0.007
		HG02069	0.206	0.213	0.184	0.201
		HG02070	0.221	0.201	0.206	0.210
		HG02072	0.314	0.336	0.294	0.315
		HG02073	0.130	0.139	0.090	0.120
		HG02075	0.145	0.130	0.091	0.122
		HG02076	0.134	0.134	0.109	0.126
		HG02130	0.127	0.128	0.098	0.118
		HG02131	0.019	0.014	0.000	0.011
		HG02512	0.159	0.158	0.117	0.144
		HG02513	0.306	0.313	0.294	0.304
		HG02521	0.139	0.129	0.066	0.111
		HG02522	0.225	0.215	0.191	0.210
		HG02524	0.168	0.169	0.175	0.171
		HG02525	0.060	0.044	0.032	0.045
	**KHV Mean**		**0.165**	**0.163**	**0.135**	**0.154**
**EUR**	**FIN**	HG00181	0.180	0.184	0.158	0.174
		HG00185	0.149	0.152	0.116	0.139
		HG00190	0.012	0.009	0.000	0.007
		HG00267	0.010	0.008	0.003	0.007
		HG00273	0.121	0.126	0.120	0.122
		HG00277	0.056	0.056	0.051	0.054
		HG00280	0.017	0.023	0.000	0.014
		HG00284	0.256	0.251	0.199	0.235
		HG00351	0.028	0.030	0.034	0.031
		HG00358	0.154	0.141	0.126	0.140
		HG00366	0.172	0.153	0.141	0.155
		HG00369	0.158	0.154	0.145	0.152
		HG00371	0.088	0.105	0.091	0.095
		HG00372	0.174	0.172	0.120	0.155
	**FIN Mean**		**0.113**	**0.112**	**0.093**	**0.106**
	**GBR**	HG00096	0.023	0.022	0.029	0.025
		HG00098	0.034	0.038	0.031	0.034
		HG00101	0.090	0.093	0.080	0.088
		HG00103	0.029	0.030	0.032	0.030
		HG00107	0.018	0.019	0.000	0.013
		HG00108	0.190	0.184	0.178	0.184
		HG00109	0.056	0.059	0.063	0.059
		HG00115	0.026	0.027	0.000	0.017
		HG00116	0.066	0.085	0.081	0.077
		HG00117	0.032	0.030	0.024	0.029
		HG00119	0.119	0.115	0.110	0.115
		HG00131	0.011	0.012	0.004	0.009

Super population	Population	Sample ID	pos. 594	pos. 621	pos. 666	Mean
		HG00136	0.019	0.016	0.005	0.014
		HG00139	0.160	0.177	0.160	0.166
		HG00256	0.071	0.062	0.058	0.064
		HG00264	0.276	0.280	0.238	0.265
	**GBR Mean**		**0.076**	**0.078**	**0.068**	**0.074**
	**IBS**	HG01619	0.010	0.010	0.002	0.007
		HG01620	0.056	0.054	0.040	0.050
		HG01623	0.162	0.167	0.173	0.167
		HG01624	0.049	0.054	0.060	0.055
		HG01625	0.064	0.058	0.033	0.052
		HG01626	0.057	0.052	0.051	0.053
		HG01628	0.021	0.029	0.019	0.023
		HG01630	0.053	0.050	0.032	0.045
		HG01682	0.150	0.139	0.112	0.134
		HG01684	0.214	0.217	0.168	0.200
		HG01685	0.044	0.040	0.041	0.042
		HG01686	0.017	0.025	0.027	0.023
		HG01707	0.059	0.049	0.045	0.051
		HG01708	0.016	0.014	0.000	0.010
		HG01756	0.051	0.058	0.043	0.051
		HG01757	0.134	0.130	0.102	0.122
		HG01761	0.042	0.035	0.022	0.033
		HG01762	0.088	0.085	0.093	0.089
		HG01770	0.013	0.015	0.001	0.010
		HG01771	0.069	0.064	0.025	0.053
	**IBS Mean**		**0.069**	**0.067**	**0.055**	**0.063**

Supplemental Table S15: Exonic and splice site KIV-2 variants reported previously and comparison with our data

Region	Pos in our study	Pos reported in external study	Base change	Citation	Observed in our screening
421	587	7	C>T	(3)	yes
421	590	10	A>G	(3)	no
421	594	14	A>G	(2, 3, 5)	yes
421	600	20	T>C	(5)	no
421	600	20	T>C	(2)	no
421	605	25	T>C	(5)	no
421	613	33	T>C	(3)	no
421	614	34	G>A	(3)	no
421	615	35	C>A	(3)	no
421	621	41	T>C	(2, 3, 5)	yes
421	632	52	A>G	(3)	no
421	640	60	C>T	(3)	yes
421	642	62	A>G	(3)	no
421	649	69	T>C	(3)	yes
421	649	69	T>C	(2)	yes
421	655	75	A>T	(5)	no
421	666	86	A>T/G	(2, 3)	yes
421	681	101	A>G	(3)	no
421	692	112	C>T	(5)	no
421	693	113	T>C	(2)	yes
421	726	146	A>G	(3)	yes
421	741	"K421 +1"	G>A	(2)	yes
422	4751	8	A>G	(5)	no
422	4761	18	A>G	(5)	no
422	4777	34	T>C	(5)	no
422	4860	117	A>T	(5)	no
422	4861	118	A>T	(5)	no
422	4901	158	A>G	(5)	no
422	4774	31	A>T	(2)	yes
422	4801	58	T>C	(2)	yes
422	4856	113	G>A	(2)	yes
422	4738	"K422-6"	T>G	(2)	yes
422	4727	"K422-17"	C>G	(2)	yes

Supplemental Table S16: PCR conditions for the colony PCR

	**Colony PCR**
**Product length**	1072 bp
**Reaction vol.**	15 µL
**Enzyme**	Agilent Herculase II
**Initial denaturation**	95° C, 2 min
**Denaturation**	95° C, 20 sec
**Annealing**	58.8° C, 20 sec
**Extension**	72° C, 40 sec
**Final extension**	72° C, 3 min
**Number of cycles**	40
**ID Primer fw** [1]	421U
**ID Primer rv** [1]	421L
**Final primer conc. [µM each]**	0.25 µM
**Final dNTP conc. [mM each]**	0.25 mM
**Enzyme amount [µl]**	0.3 µL
**DNA input [ng]**	colony

[1] **Primer sequences are given in Supplemental Table S2**

2 Supplemental Figures

Supplemental Figure S1: Explanation of the concepts of “batch sequencing” and “mutation level”, respectively ”variant level” and their effect on the interpretation of sequencing mixes of KIV-2A and KIV-2B.

Panel A) This panel exemplifies the concept of batch sequencing: All KIV-2 repeats (here represented by alternating light and dark yellow boxes) are amplified simultaneously. Any point mutation (dark blue box, representing one base differing from the reference sequence) present in a subset of kringles will be reflected in the same proportion of amplicons (black lines). This can be detected and quantified by ultra-deep sequencing. In this example the “mutation level” of the KIV-2 mutation will be 20%

Panel B) Same situation as in panel A, but with two different mutations (turquois and dark blue boxes) present on different KIV-2 repeats. These two mutations are located on different repeats, but both at the same level (1 in 5, i.e. 20%).

Panel C) The blue mutation from above is here present in two kringles of five (40% mutation level), while the turquois mutation is present in one kringle of five (20%).

Panel D) In this example, also a KIV-2 subtype B (KIV-2B) is present. This subtype of KIV-2 is characterized by several differences to the reference, which are located closely together (forming a linked haplotype; see references (2, 4) and the main manuscript (Section Methods and Results for a detailed explanation. Any amplicon stemming from this repeat will contribute several mutations, all occurring at the same “mutation level” (i.e. the same fraction of the total of sequencing reads). The lower number of amplicons depicted here relates to the under-amplification of KIV-2B repeats observed in our data.

Panel E) This Panel is the merged situation of Panels C and E and is the example which most closely resembles the in-vivo situation in people carrying both KIV-2A and KIV2-B kringles (about 4/5 of the Caucasian population in our data, see the main manuscript). According to our observations described in the main manuscript, When both KIV-2A (yellow rectangles) and KIV-2B kringles (blue

rectangle) occur in the genome, the KIV-2A are equally amplified, while KIV-2B are amplified to a minor extend. Accordingly, any mutations present on a KIV-2A (blue and turquois is reflected to the same proportions in the amplicons, respectively sequencing reads, while the KIV-2B variants (red boxes) are represented to a reduced amount, because the KIV-2B kringle is amplified less efficiently. Accordingly both the KIV-2B specific variants as well as any mutation which arose by chance on the KIV-2B is represented to a minor extent in the sequencing reads (“mutation level” is reduced).

For simplification, only a haploid status is shown. Chromosomal location of a situation like Panel B cannot be resolved by NGS.

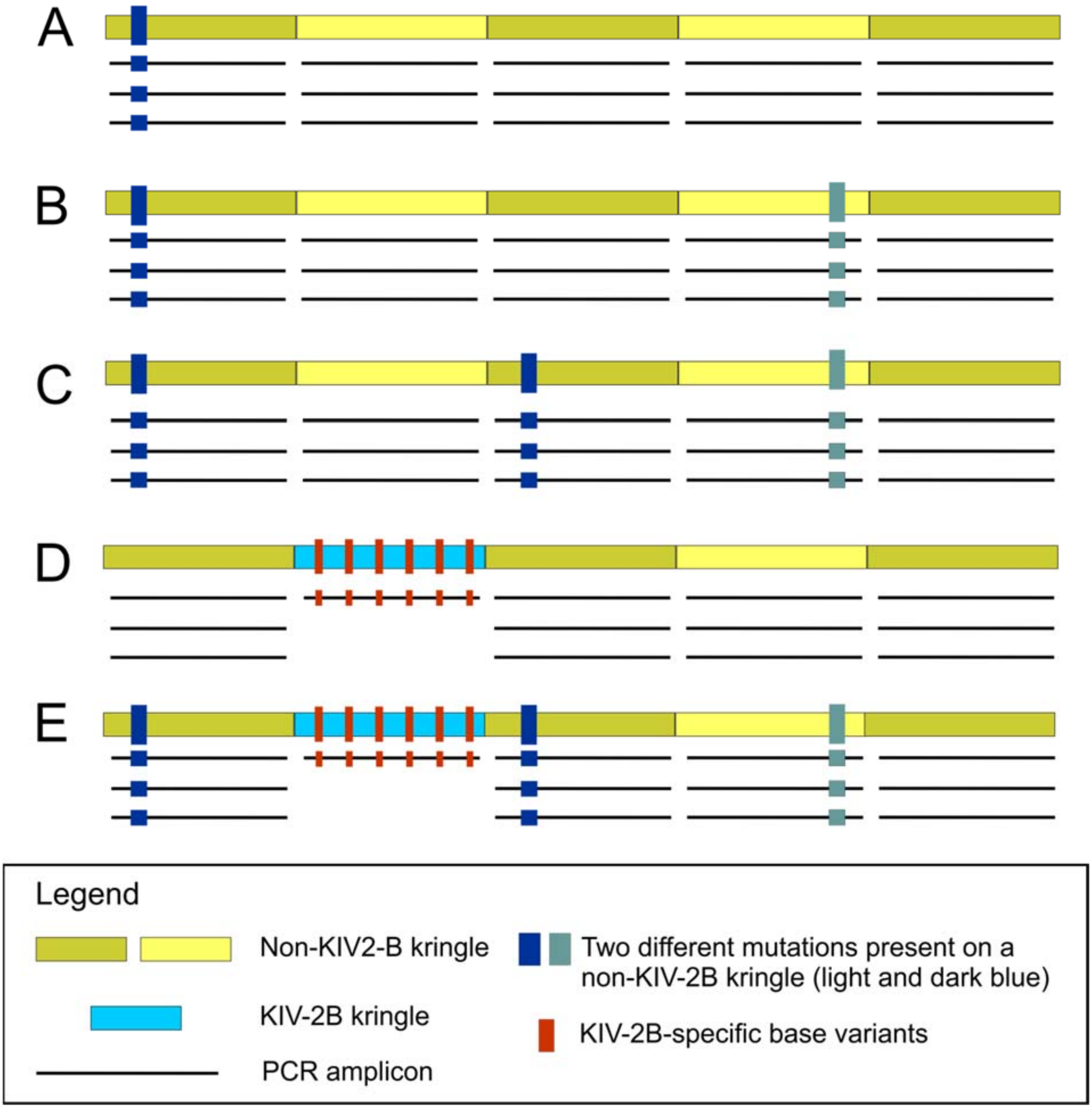

Supplemental Figure S2: PCR amplicon design.

The figure shows a portion of the KIV-2 locus in 5’ to 3’ transcription direction. Boxes with same color indicate the two exons of each KIV-2 repeat. The dotted arrows indicate the relative location of the amplicons (image is not in scale).

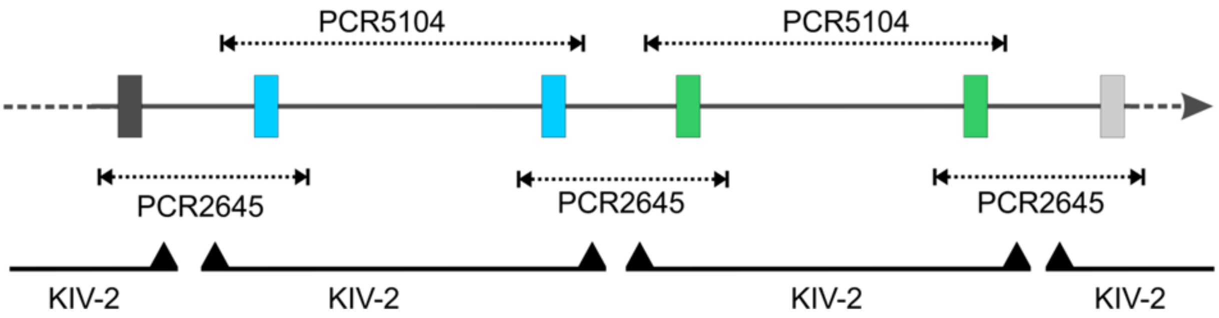

Supplemental Figure S3: Canonical KIV-2 subtype variants

Haplotypes in the KIV-2 exon 1 defining the KIV-2 subtypes A, B and C. (3, 4). The positions 14, 41 and 86 refer to exon 421 and correspond to positions 594, 621 and 666 in our PCR5104 reference sequence.

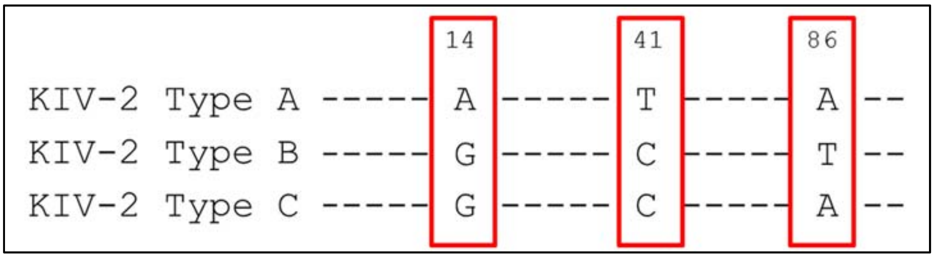

Supplemental Figure S4: Example of positions, which are erroneously pruned by the BAQ algorithm.

These variants generate stretches of consecutive low level differences in a batch sequencing approach (figure shows positions 417 to 596 of fragment PCR5104), which is recognized as highly improbable by BAQ and thus (errounously) pruned.

Alignment created using ClustalO(6).

```
KIV2B_3(-)5101bp      CTGTATGTTTTTTGGTTGGTTTTCTCCCATCCATCTGCCTACAGGTAAGGGAAAGATAAC
KIV2_5(-)5098bp       gtgtttgtttgttggtcggttttctcacatccatctgcctatggataaggaaaagagaac
KIV2_2(-)5096bp       gtgtttgtttgttggtcggttttctcacatccatctgcctatggataaggaaaagagaac
KIV2_4(-)5096bp       gtgtttgtttgttggtcggttttctcacatccatctgcctatggataaggaaaagagaac
KIV2_1(-)5099bp       gtgtttgtttgttggtcggttttctcacatccatctgcctatggataaggaaaagagaac
KIV2_6(-)5104bp       gtgtttgtttgttggtcggttttctcacatccatctgcctatggataaggaaaagagaac
                       *** ***** ***** ********* **************  * ***** ***** ***

KIV2B_3(-)5101bp      GTTCGTAATTCTCATAGACTCCTTTCTGGTTGTGTCATAAATGGCTTCACATATTTCGTT
KIV2_5(-)5098bp       ggtcgtaattctcatagactcctttctggttgtgtcacaaatggcttcacatgtttctct
KIV2_2(-)5096bp       ggtcgtaattctcatagactcctttctggttgtgtcacaaatggcttcacatgtttctct
KIV2_4(-)5096bp       ggtcgtaattctcatagactcctttctggttgtgtcacaaatggcttcacatgtttctct
KIV2_1(-)5099bp       ggtcgtaattctcatagactcctttctggttgtgtcacaaatggcttcacatgtttctct
KIV2_6(-)5104bp       ggtcgtaattctcatagactcctttctggttgtgtcacaaatggcttcacatgtttctct
                      * *********************************** ************** ****  *

KIV2B_3(-)5101bp      ATTCTCAGAGATACTCAGTTT-ATTTCTTGTGTTTTCATTTCAGCACCGACTGAGCAGAG
KIV2_5(-)5098bp       atgctcagagatactcagcttgatttcccgtgttttcatttcagcaccgactgagcaaag
KIV2_2(-)5096bp       atgctcagagatactcagcttgatttcccgtgttttcatttcagcaccgactgagcaaag
KIV2_4(-)5096bp       atgctcagagatactcagcttgatttcccgtgttttcatttcagcaccgactgagcaaag
KIV2_1(-)5099bp       atgctcagagatactcagcttgatttcccgtgttttcatttcagcaccgactgagcaaag
KIV2_6(-)5104bp       atgctcagagatactcagcttgatttcccgtgttttcatttcagcaccgactgagcaaag
                      ** *************** ** *****  **************************** **
```

Supplemental Figure S5: Concept of the sequencing calibration using KIV-2A and KIV-2B variants

This figure explains the concept of the plasmid-based calibration and evaluation of the pipeline. Two plasmids, containing either a KIV-2A or a KIV-2B insert were mixed at different ratios (50:50, 90:10, 95:5, 97.5:2.5, 98.5:1.5 and 99:1), with the representing always the minor component to recapitulate the in-vivo situation where KIV-2B repeats are rarer. These mixes were used as template for ultra-deep batch sequencing.

Each KIV-2B contains 60 differences to the reference, indicated by the red lines, which are present in al inked manner on the same molecule. Therefore they shall all occur at (approximately) the same level in the reads. This allows testing sensitivity over a broad range of sequence contexts and evaluating the stability of the mutation level determination because always 60 positions are probed simultaneously. The figure below exemplifies this for mixture levels 50:50 (50%) and 90:10 (10%). Variants to the reference present on the KIV-2A plasmid are omitted for simplification.

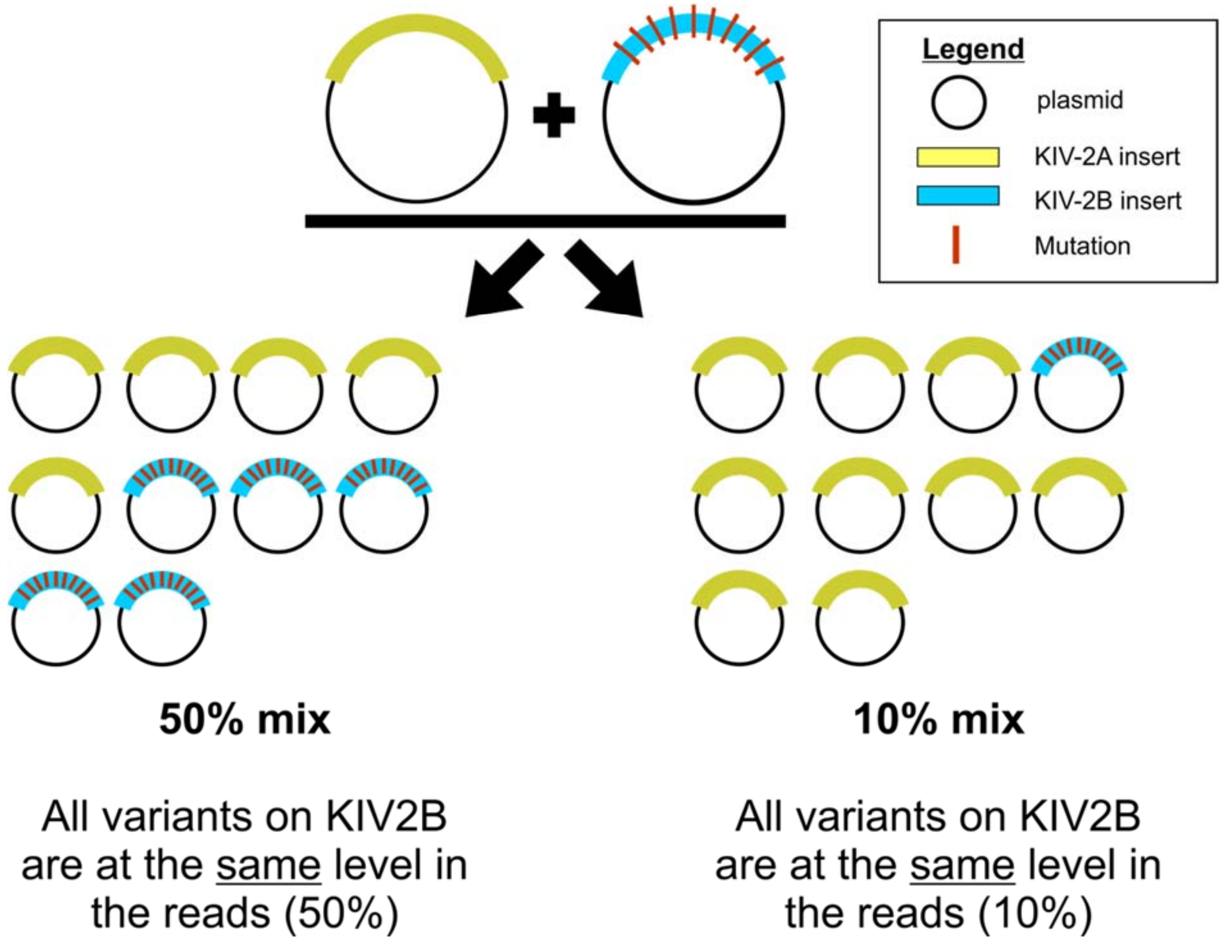

Supplemental Figure S6: Impact of the polymerase on the KIV-2B variation level detected in plasmid mixes for the inter-KIV-2 intron (fragment PCR2645).

Three different polymerases were evaluated regarding their precision in reproducing the expected variation level of mixes of plasmids containing a KIV-2A and KIV-2B insert. The expected variation level is defined by the proportion of KIV-2B plasmid in the mix. 110 base differences distributed over the insert were required to be detected (see the Methods section for an explanation of the expected number of differences). No pronounced impact of the polymerase was observed.

Since the pipeline requires a minimum of 1% for a variant to pass quality control, at very low mixture levels the number of variants on which the plots are bases (n) partially decreases, since some base may be underamplified due to technical, sequence context reasons or others. These variants are thus filtered out. Since no level below 1.2% percent is expected in the population (1 mutant kringle in 80 KIV-2 repeats) a hard limit at 1% provides a measure to guarantee the specificity of the mutation detection(7).

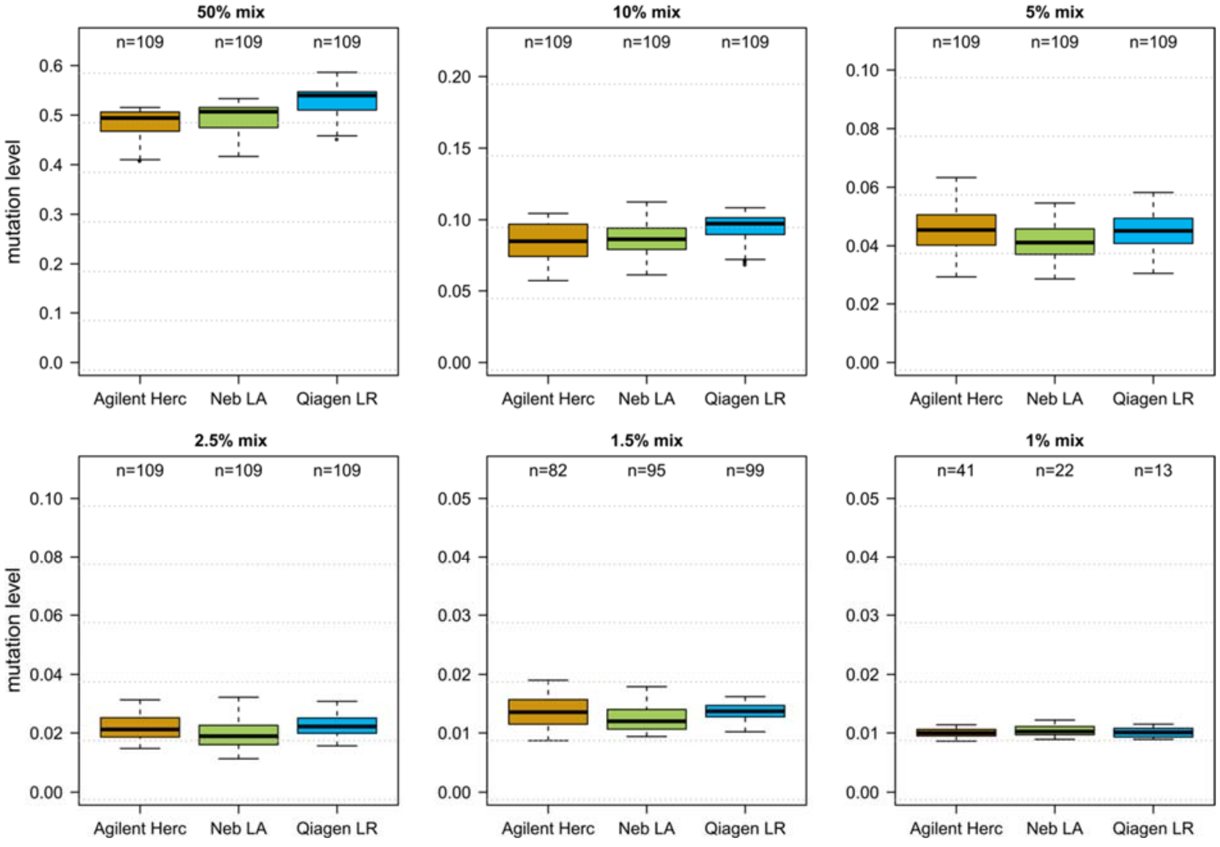

Supplemental Figure S7: Number of discovery samples with a variant at a given position.

Top panel (A): all positions (including the KIV-2B positions).

Bottom panel (B): only non KIV-2B variation (shown also in the main manuscript).

The location of intron-exon structures is given between the panels (5' to 3' is given left to right; please note that in the genomic context, *LPA* is coded on the minus strand). black: non-coding variation; blue: near-splice site variants (±25 bp), orange: synonymous variants; red: missense variants. The frequent missense variant at the 3' end of exon 2 is KIV-2 4925G>A (1). The frequent synonymous variants in exon 1 in the top panel are the three canonical KIV-2B variants.

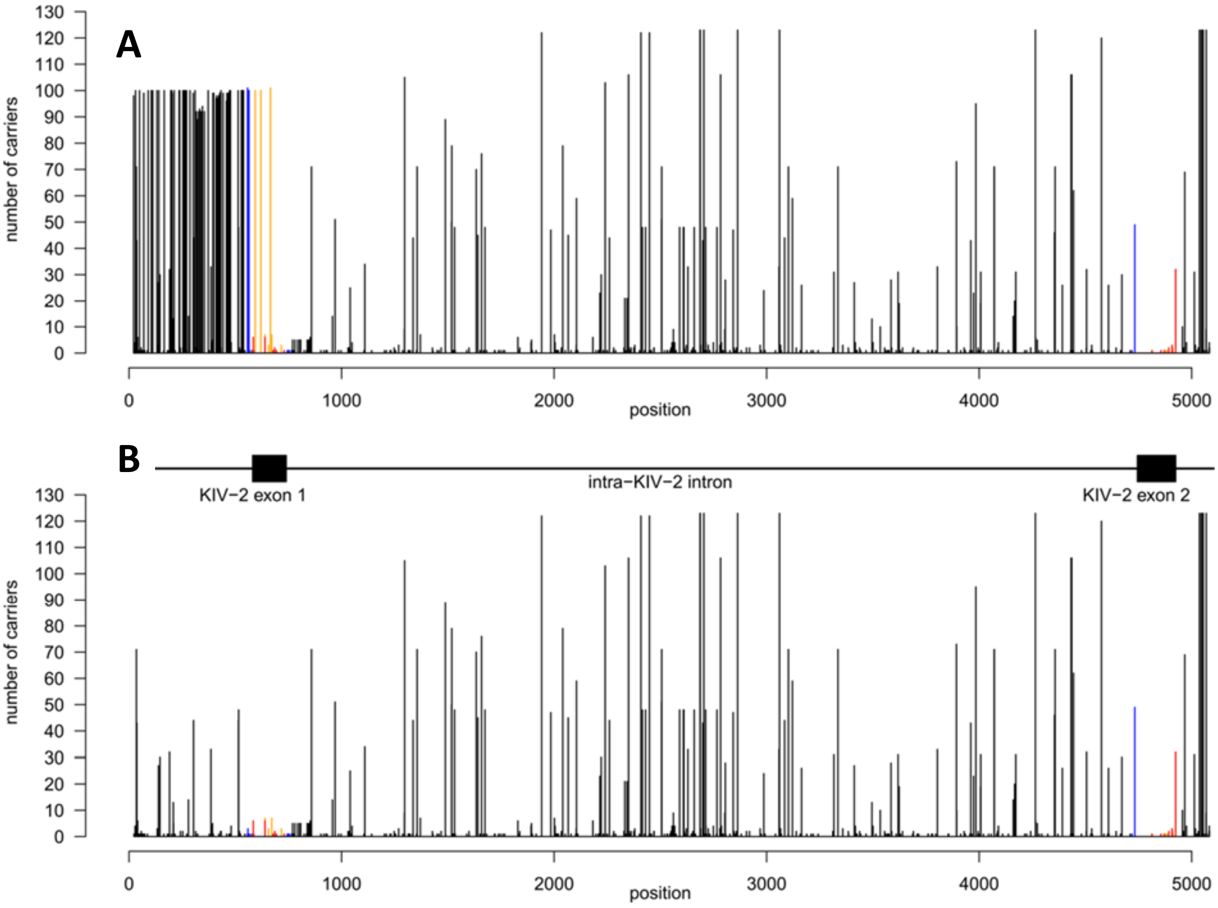

Supplemental Figure S8: Variation levels of the KIV-2 variants found in the discovery set (without KIV-2B variants).

The variation levels (var level, given on the y axis) varied widely, with some variants reaching nearly 100% level. N.b. only individuals showing a variation at the respective position are included in the plot. Therefore each boxplot encompasses a variable number of individuals. The number of carriers, irrespective of the variant level is reported in Supplemental Figure S6.

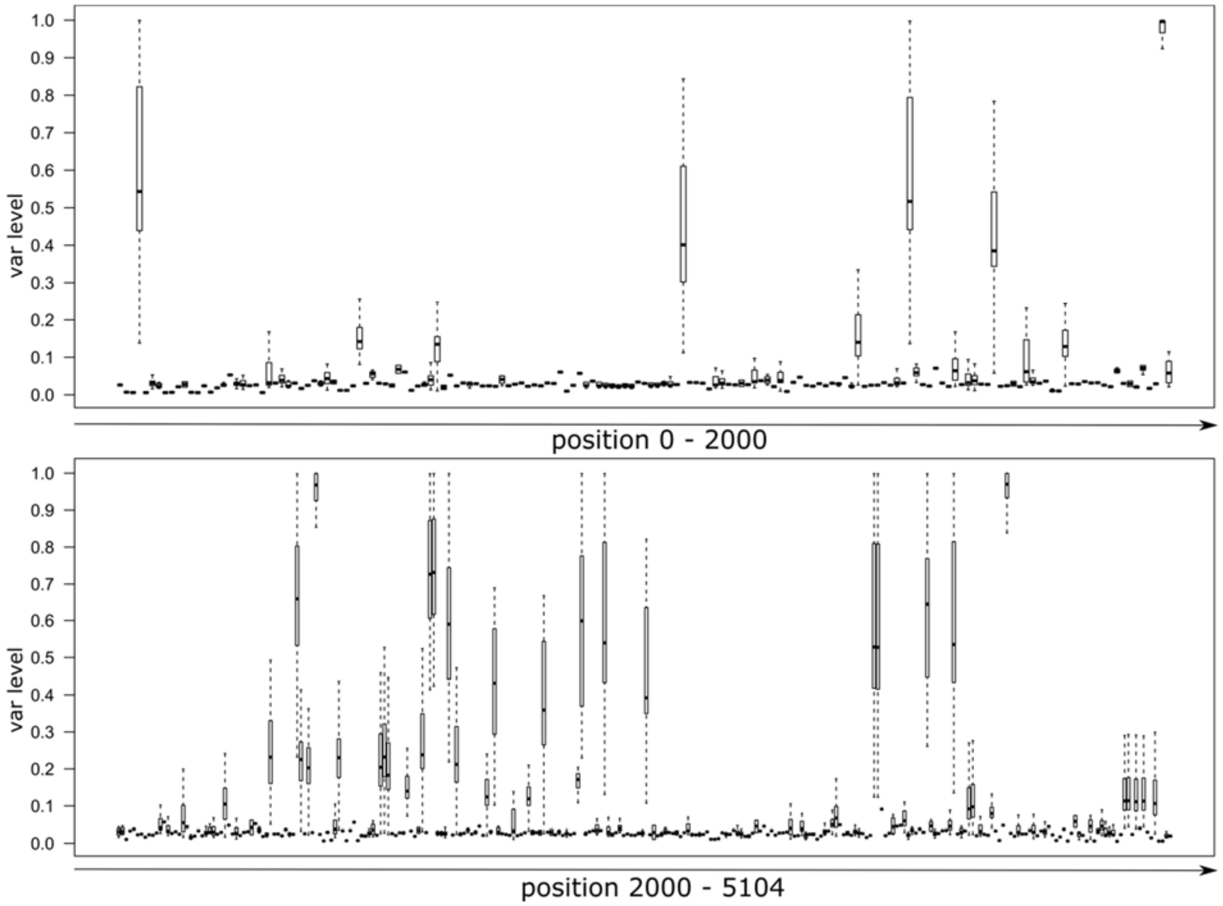

Supplemental Figure S9: Number of discovery samples with a variant at a given exonic position (excluding both KIV-2B).

Red: missense variants, orange: synonymous variants, blue: near-splice site variants (±25 bp). For better readability, the prefixes according to HGVS have been omitted.

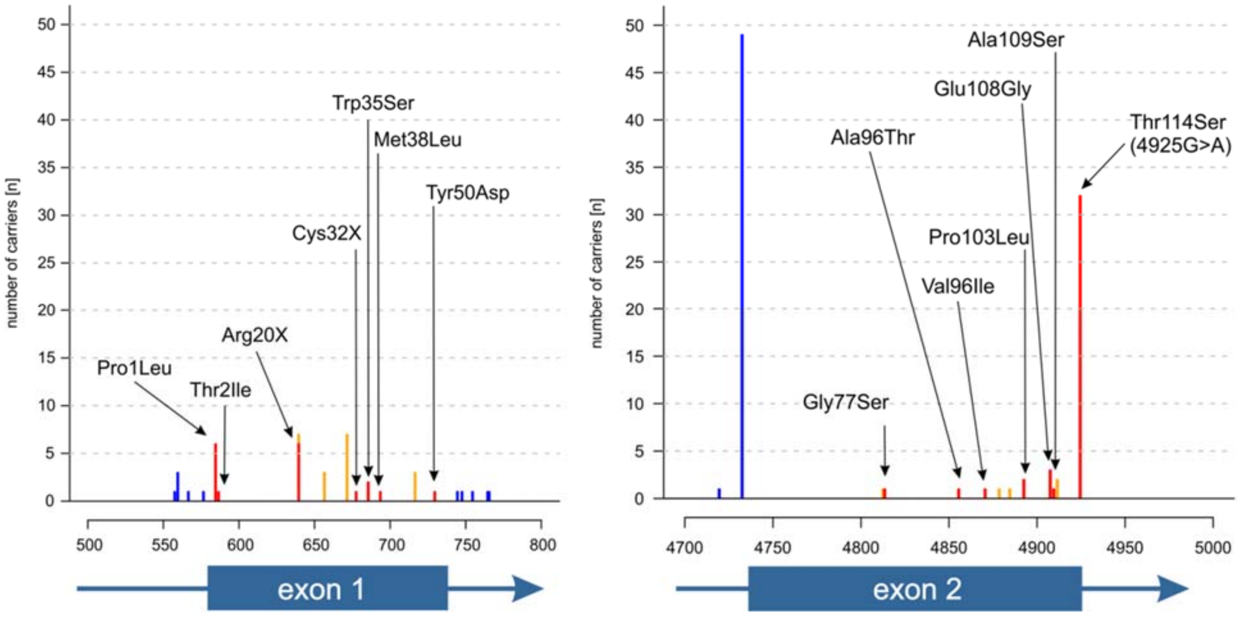

Supplemental Figure S10: KIV-2B mutation levels and number of KIV-2 repeats carrying the KIV-2B haplotype in the discovery sample set.

KIV-2B mutation levels (left) and number of KIV-2 repeats (right) carrying the KIV-2B haplotype in the discovery sample set (n=100). As would be expected, the variant at position 666 shows a lower mutation level, because the variants at position 594 and 621 are carried by both KIV-2B and KIV-2C, while variant 666 is present only on KIV-2B. Therefore less kringles contribute.

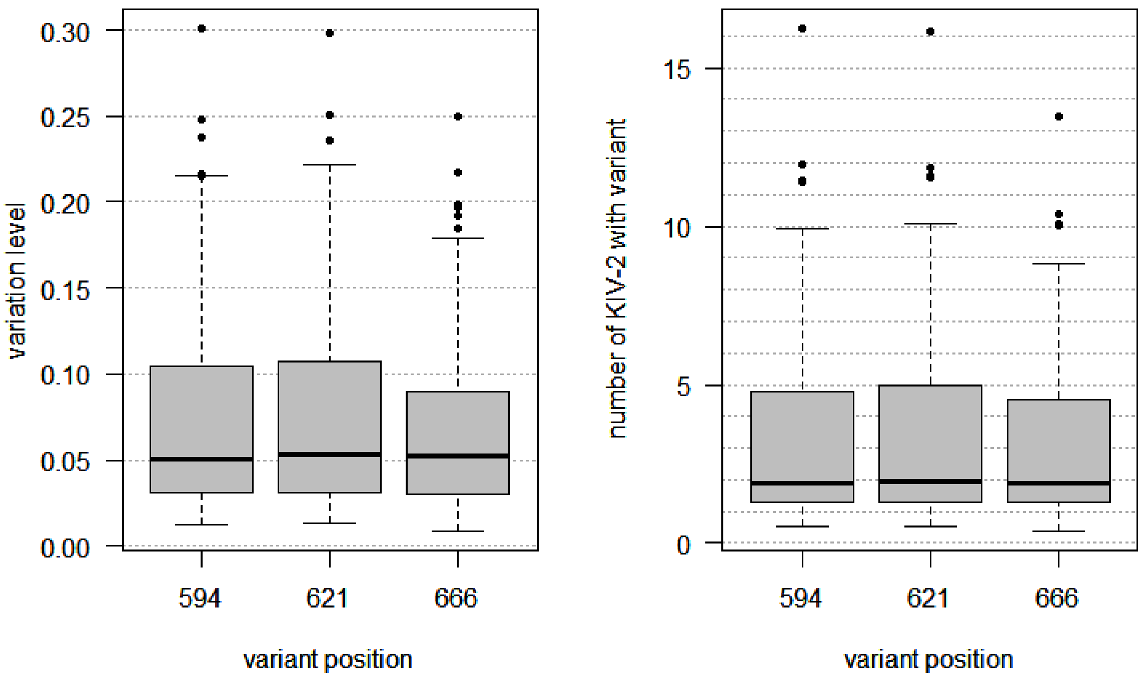

Supplemental Figure S11: 2016G>A validation. DraIII digestion of selected samples carrying the 2106G>A variant.

Wet-lab validation shows that all samples supposed to carry the 2106G>A variant according to NGS ("+") also present the DraIII digestion pattern and fragment sizes according to Mancini et al(8). The "-" sign denotes randomly picked samples, where no 2106G>A variant was detected in NGS. The migration position of the two digestion fragments is marked by red arrows (one per gel). Different intensities of the digestion fragment indicate different mutation level, i.e. a different number of KIV-2 repeats carrying the variant (as in Main Figure 6). The mutation levels in NGS were: sample 1: 13.0%; sample 2: 6.1%; sample 3: 0.0%; sample 4: 0.0%; sample 5: 8.9%; sample 6: 0.0%; sample 7: 4.0%; sample 8: 9.1%; sample 9: 0.0%; sample 10: 0.0%; sample 11: 3.9%; sample 12: 22.4%; sample 13: 0.0%; sample 14: 0.0%. PC: PCR positive control (control DNA; by chance seemingly carrying 2106G>A at very low level, too). DC: Digestion control (commercial Lambda phage DNA; digestion pattern is as expected).

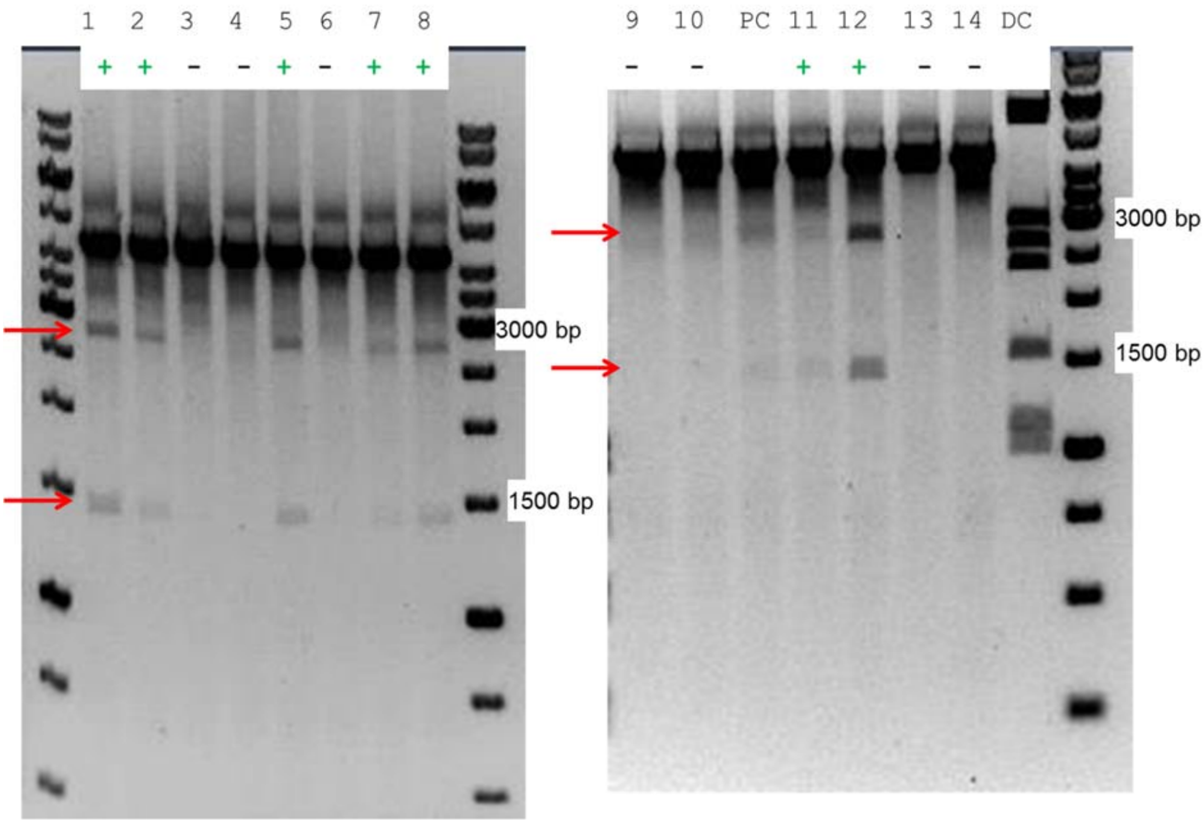

Supplemental Figure S12: Per-base identity between the exons of *LPA* and between the sequences of *LPA* and *LPAL2*.

A) The matrices report the pairwise per-base identity (i.e. the percentage of identical bases in each pairwise the alignment) for all kringle IV repeats and the kringle V. The color code ranges from green (low per-base identity) to dark red (perfect identity of each base in the alignment). All KIV share extreme homologies, which tend to decrease with increasing distance from each other but are still >70% and often even >85%. For the alignments with the intronic contents the sequence has been extended by 200 bp in each direction of the exon. This shows that the homologies extend into the introns, albeit to a lower extent (mostly >50% identical bases), which allows isolating single kringles in the PCR and in the read alignment. **B)** Per-base identity between the transcribed pseudogene LPAL2(9) and LPA KIV-6 to KV. The pseudogene misses the region KIV-1 to KIV-5. K4 stands for KIV, K5 for KV. Sequences based on hg19 aligned using ClustalW(10)

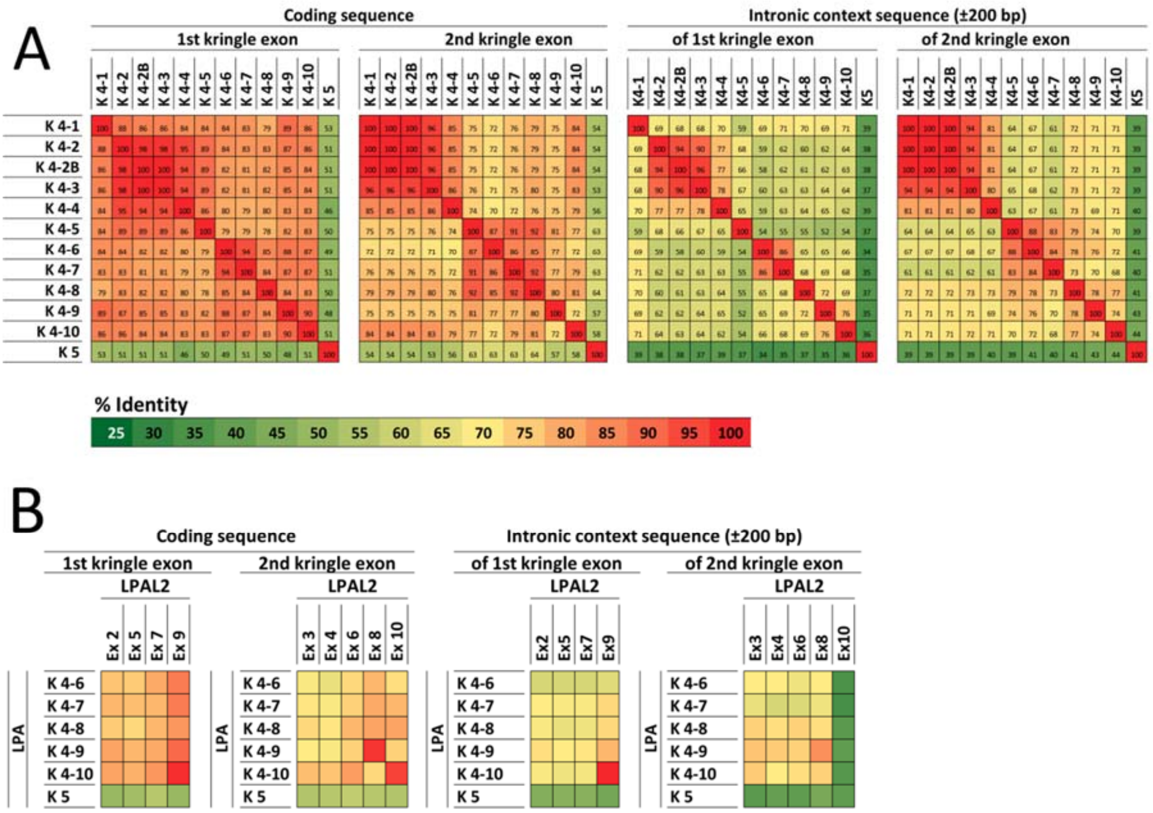

Supplemental Figure S13: Venn diagram showing the overlap of all coding (A and B) and all missense variation (C and D; includes nonsense and splice site variants) based on DNA level.

Panels A and C include all variants (A, C) while panels B and D are restricted to variants observed at least twice. Variants according to Supplemental Table S9.

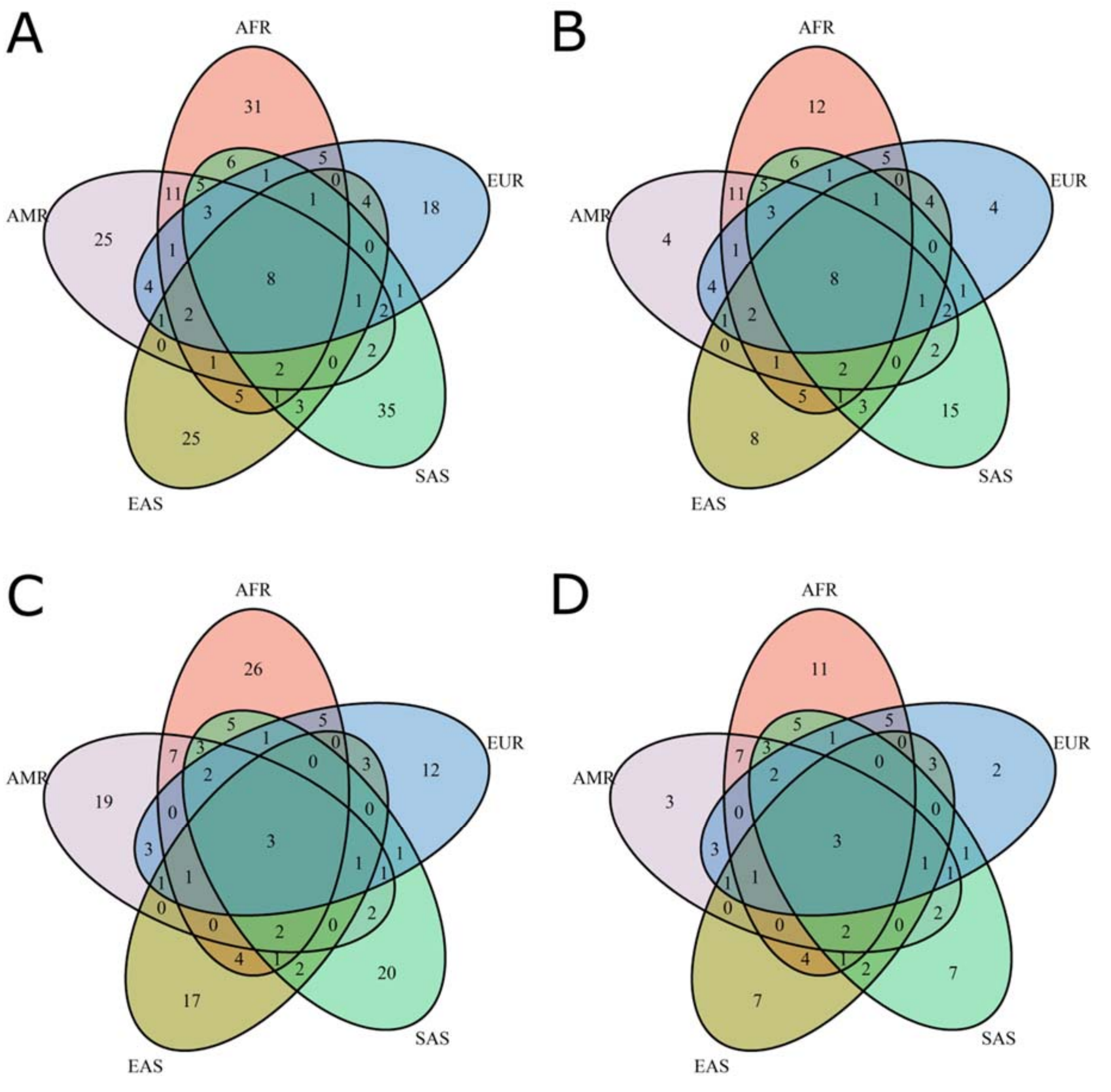

Supplemental Figure S14: Expected digestion pattern of NcoI + BsrI digestion used to determine the KIV-2 subtypes of colony PCR amplicons.

The NcoI and the 5’ BsrI restriction sites are located on the canonical KIV-2B variants 621 and 666

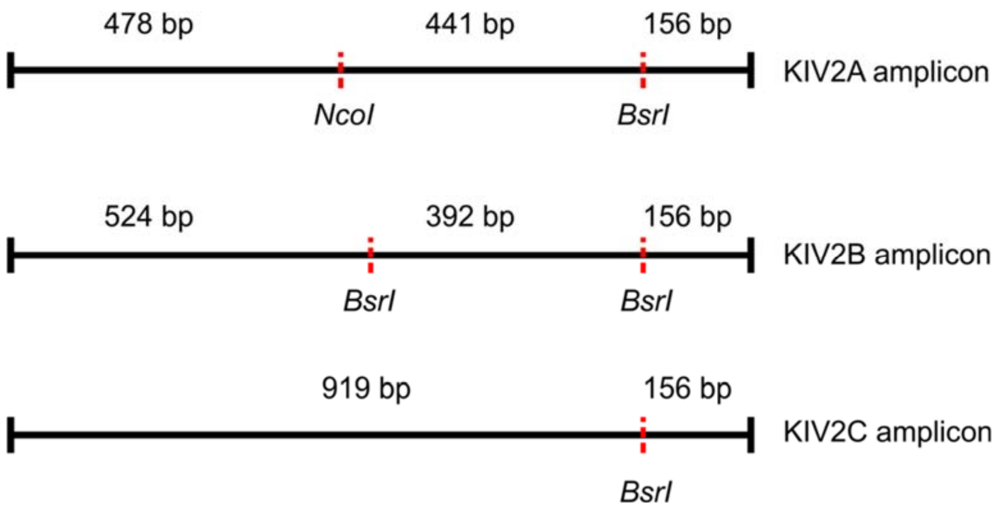

3 Supplemental Methods

3.1 Reference sequence and definition of "KIV-2B-specific variants"

An unambiguous nomenclature according to HGVS (11) is not possible, since the *LPA* RefSeq sequence contains six KIV-2 repeats, but an exact assignment of a variant to a certain kringle repeat is not possible when using batch sequencing. Moreover, the official human genome reference contains 6 KIV-2 repeats: 5 type A and one type B (repeat 3 in transcription direction) (2). However, only about 2% of the population presents such a short allele. The reference repeats all slightly differ in their length and base sequence (Supplemental Table S5, Supplemental alignment). Therefore, we reference all PCR5104 data to the sixth repeat in hg19 (in 5' to 3' direction on the minus strand of hg19; note that *LPA* is encoded on the minus strand) as defined by the binding of the PCR amplification primer (chr6:161,033,785-161,038,888), because this KIV-2A repeat represents the longest reference repeat element in hg19. This minimizes the need of calling insertions during NGS data analysis, which introduces additional difficulties to short-read NGS (12). Accordingly, the PCR2645 reference is the inter-kringle intron preceding the PCR5104 reference repeat as defined by binding of the amplification primers (chr6:161,037,674-161,040,318).

Three KIV-2 subtypes (KIV-2A, KIV-2B and KIV-2C), which are defined by the haplotype of three synonymous variants in the first KIV-2 exon (Supplemental Figure S10) have been described (3, 4). Alignments of the six reference genome repeats show that the KIV-2B variant includes several additional differences also in the introns (exemplified in Supplemental Figure S4 and Supplemental Alignment). In this paper we will henceforth use the wording "KIV-2B variant" for *all* differences to the reference, for which the following three conditions applied: A) the variant was present in the cloned KIV-2B kringle (see next subsection), B) the variant was different from the reference sequence as defined above and C) was not an indel (see column "defined as KIV2-B" in (Supplemental Table S1 and Supplemental Table S2). This leaves 60 and 110 variants for PCR5104 and PCR2645, which were required to be detected in the calibration experiments. This includes also all intronic differences. The three exonic variants described by McLean (4) and by Parson (3) are referred to as "canonical KIV-2B

variants" and are located at exon 1 positions 14, 41 and 86 (2), respectively at positions 594, 621 and 666 in our reference sequence PCR5104).

Sequence alignments were done using ClustalO (6) online and within the Benchling software suite (www.benchling.com)

3.2 Cloning of KIV-2A and KIV-2B

We isolated a KIV-2A and a KIV-2B amplicon by PCR subcloning and used them to generate defined clone mixes differing by several variants over the whole region, which were then used to generate calibration data for the bioinformatic analysis pipeline. Both PCR fragments were amplified using Agilent Herculase II Fusion (Agilent Technologies Inc., Santa Clara, CA, USA) (Supplemental Table S14), cloned into a pCR-XL-TOPO vector using the TOPO® XL PCR Cloning Kit (ThermoFisher Scientific) and transformed into NEB (Ipswich, MA, USA) Turbo E. coli chemically competent cells. Positive clones were subjected to colony PCR (Supplemental Table S16). The PCR fragments were then sequentially digested by NcoI and BsrI (both NEB). The combined restriction pattern allows discriminating KIV-2A, KIV-2B and KIV-2C repeats (Supplemental Figure S13). NcoI digestion was performed in 15 µl reactions with 0.3 U enzyme and NEB Buffer 3.1 (1 h 37°C, then 20 min at 80°C). Two µl of this reaction were replaced by 0.4 U of BsrI in 1.7 µl NEB Diluent B and 0.2 µl Buffer 3.1 (1 h at 65°C, then 20 min at 80 °C). Clone subtype was confirmed by sequencing the canonical KIV-2B positions. One KIV-2A and one KIV-2B clone for both PCR fragments were randomly chosen for further analysis. The complete inserts of all clones were then resequenced using Sanger sequencing (ABI 3130xl sequencer using BigDye v1.1 chemistry according to manufacturer protocol with minor modifications).

3.3 NGS sequencing protocol

The PCR products were purified using the QIAquick PCR Purification Kit (Qiagen, Hilden, Germany) and quantified using the Qubit HS Kit (ThermoFisher Scientific, Waltham, MA, USA). 200 ng

of amplicon were fragmented to ≈700 bp (677 ± 73 bp (mean ± SD)) using NEBNext® dsDNA Fragmentase (NEB, Ipswich, MA, USA) (incubation time 9.3 ± 1.6 min (mean±SD) at 37°C) and subjected to the Illumina TruSeq Nano library generation workflow (Illumina, Inc., San Diego, CA, USA). Sequencing was performed on an Illumina MiSeq system using reagent kit v2 with 500 cycles. A detailed step-by-step pipetting protocol is provided at https://github.com/genepi/lpa-pipeline.

3.4 Populations for de-novo amplicon sequencing ("discovery set")

The populations used to generate KIV-2 variability data have been described earlier (1, 13, 14). The Salzburg Atherosclerosis Prevention Program in subjects at High Individual Risk (**SAPHIR**) is an observational study conducted in the years 1999-2002 involving healthy unrelated subjects: 645 females from 39 to 67 years of age and 1093 males from 39 to 66 years of age. Study participants were recruited by health screening programs in companies in area of the city of Salzburg in Austria. Lp(a) phenotyping by ELISA and Western Blot was available for n=1,522 individuals. The German Chronic Kidney Disease **(GCKD) study** (14) is an ongoing prospective observational national cohort study including 5,217 Caucasian patients with CKD of moderate severity from different etiologies. Lp(a) phenotyping by ELISA and Western Blot was available for n=4,949 participants. All individuals were of Western European origin. The studies were approved by the respective institutional review boards (IRB).

As described previously(1), 123 samples were drawn from these populations following a discordant phenotype rationale to enrich for functional variants: 80 samples which deviated from the known correlation between apo(a) isoforms and Lp(a) concentrations were selected (i.e. samples with low Lp(a) despite LMW or with high Lp(a) despite HMW) and complemented by 43 controls with the expected apo(a) isoform to Lp(a) level correlation. Details are given in reference (1). In all 123 samples, the PCR5104 fragment was screened for mutations by ultra-deep sequencing as described above.

3.5 Lp(a) measurement and *LPA* characterization

Lp(a) concentration was determined by ELISA as described before (15, 16). Lp(a) Western blotting (15) was used as surrogate for genotyping with the advantage of additional information concerning the expression of the protein. Since only 60-70% of all individuals present both apo(a) alleles in plasma, the total number of KIV-2 repeats was estimated by qPCR using the exon 4 protocol of Lanktree et al (17) with minor modifications described in the Supplemental Materials of reference (1). Copy number was calculated using a calibrator sample with the Western Blot allele size 25/30, i.e. 55 KIV repeats. 95% confidence interval of the KIV-2 copy number was calculated according to Weaver et al, 2010 (18).

3.6 1000 Genomes data

The 1000 Genomes project is a freely accessible public data resource including data from 2,504 individuals in its final phase (so called 1000 Genomes Phase 3). High coverage exome sequencing data of all individuals is available at ftp://ftp.1000genomes.ebi.ac.uk/vol1/ftp/phase3/data. Additionally, high coverage whole genome sequencing (WGS) data from 150 of these individuals was made publicly available by the HiSeqX Diversity Panel of the Illumina Polaris project (https://github.com/Illumina/Polaris/wiki/HiSeqX-Diversity-Panel; (https://www.ebi.ac.uk/ena/data/view/PRJEB20654).

We downloaded and extracted all reads mapping to the *LPA* KIV-2 region (GRCh37, chr6:161033785-161066618) from both datasets. For the exome data, SAMtools (19) was used to download the data in BAM format and converted to FASTQ using BEDtools2 (20). For the Polaris data, the sratoolkit (https://www.ncbi.nlm.nih.gov/sra/docs/toolkitsoft/) has been used to download the data in SAM format and converted again to FASTQ using SAMtools and BEDtools2. All necessary scripts are available in our GitHub repository (https://github.com/genepi/lpa-pipeline).

3.7 Restriction digestion to validate the molecular basis of the “DraIII polymorphism”

Amplification was carried out with the primer described by Mancini et al (8) in 25 µl using Agilent Herculase II (0.5 µl and 100 ng of template). Cycling conditions were 95°C 2 min, followed by 30 cycles of 95°C 20 sec, 67.5°C 20 sec, 72°C 2.5 min and a final extension step of 72°C for 3 min. The PCR product was purified using the QIAGEN Qiaquick PCR purification spin column kit, quantified and 600 ng were digested with 2 u DraIII (NEB) in NEB CutSmart buffer (37°C, 6 h). The results were visualized on agarose gels.

4 Supplemental Alignment

Alignment of PCR5104 for all six repeats in the reference sequence

Sequence based on hg19. Differences are highlighted. The number after the subscript refers to the order of the KIV-2 repeat in transcription direction. KIV-2_6 was used as reference sequence. The fragments are defined by the binding of the PCR Primer

CLUSTAL O(1.2.1) multiple sequence alignment

```
KIV2B_3(-)5101bp      TCAGGATGCAGGGCATGAGAAGATTCCCTCCCTCCTCCAAGGGAAGAAGGTTTTGGCGTG
KIV2_5(-)5098bp       tcaggatgcagggcatgagaggattccctctctcctccaagggaagaagcttttggcgtg
KIV2_2(-)5096bp       tcaggatgcagggcatgagaggattccctctctcctccaagggaagaagcttttggcgtg
KIV2_4(-)5096bp       tcaggatgcagggcatgagaggattccctctctcctccaagggaagaagcttttggcgtg
KIV2-1(-)5099bp       tcaggatgcagggcatgagaggattccctctctcctccaagggaagaagcttttggcgtg
KIV2_6(-)5104bp       tcaggatgcagggcatgagaggattccctctctcctccaagggaagaagcttttggcgtg
                      ******************** ********* ****************** **********

KIV2B_3(-)5101bp      CACACATCCTTGAGAAGCAAAGTGTCTTTGCCTTCAGTCAGATATATAGGATCGTTTTCT
KIV2_5(-)5098bp       cacacatccctgagaagcaaagtgtctttgtcttcagtcagatacataggaccgttttct
KIV2_2(-)5096bp       cacacatccctgagaagcaaagtgtctttgtcttcagtcagatacataggaccgttttct
KIV2_4(-)5096bp       cacacatccctgagaagcaaagtgtctttgtcttcagtcagatacataggaccgttttct
KIV2-1(-)5099bp       cacacatccctgagaagcaaagtgtctttgtcttcagtcagatacataggaccgttttct
KIV2_6(-)5104bp       cacacatccctgagaagcaaagtgtctttgtcttcagtcagatacataggaccgttttct
                      ********* ******************** ************* ****** ********

KIV2B_3(-)5101bp      GCCCCATGGCCTGGAAGCCAGAGGCCTTGGCTTTCATGATCAACGATCTAGGGAAACATG
KIV2_5(-)5098bp       gccccatggcccggaagccaaaggccttggctttcatgatcaacggtctagggaaacatg
KIV2_2(-)5096bp       gccccatggcccggaagccaaaggccttggctttcatgatcaacggtctagggaaacatg
KIV2_4(-)5096bp       gccccatggcccggaagccaaaggccttggctttcatgatcaacggtctagggaaacatg
KIV2-1(-)5099bp       gccccatggcccggaagccaaaggccttggctttcatgatcaacggtctagggaaacatg
KIV2_6(-)5104bp       gccccatggcccggaagccaaaggccttggctttcatgatcaacggtctagggaaacatg
                      *********** ******** ************************ **************
```

```
KIV2B_3(-)5101bp      CAAAATTTCCATGTCTTTCCCCTCCTCTGCCCTCGACAGCCAATTACCACCTGCATCCTG
KIV2_5(-)5098bp       caaaatttccatgtctgtcccaaactcttcccccgacagccaattaccacctgcagcccg
KIV2_2(-)5096bp       caaaatttccatgtctgtcccaaactctgcccccgacagccaattaccacctgcagcccg
KIV2_4(-)5096bp       caaaatttccatgtctgtcccaaactctgcccccgacagccaattaccacctgcagcccg
KIV2-1(-)5099bp       caaaatttccatgtctgtcccaaactctgcccccgacagccaattaccacctgcagcccg
KIV2_6(-)5104bp       caaaatttccatgtctgtcccaaactctgcccccgacagccaattaccacctgcagcccg
                      **************** ****   **** *** ********************** ** *

KIV2B_3(-)5101bp      CATTGCCAAATGCAGTGCCCTTTGTATGAACATTCAGTAGAGTTTCATAGAAAGGTGCTA
KIV2_5(-)5098bp       cattgccaaatgcggtgccgtttgcatgaagattcagtagagtttcctagaaaggtgcta
KIV2_2(-)5096bp       cattgccaaatgcggtgccgtttgcatgaagattcagtagagtttcctagaaaggtgcta
KIV2_4(-)5096bp       cattgccaaatgcggtgccgtttgcatgaagattcagtagagtttcctagaaaggtgcta
KIV2-1(-)5099bp       cattgccaaatgcggtgccgtttgcatgaagattcagtagagtttcctagaaaggtgcta
KIV2_6(-)5104bp       cattgccaaatgcggtgccgtttgcatgaagattcagtagagtttcctagaaaggtgcta
                      ************* ***** **** ***** *************** *************

KIV2B_3(-)5101bp      CTTCGTGAGCGCACTTTGCAGTGAGAAGGAGTCTGTTCTGTTCTGTTTTTCTAAGGATTT
KIV2_5(-)5098bp       cctcgtgagctcactttccaatgaggaatctgatc---tgttgtgttt-ctctaaggtgt
KIV2_2(-)5096bp       cctcgtgagctcactttccaatgaggaatctgatc---tgttgtgttt-ctctaaggtgt
KIV2_4(-)5096bp       cctcgtgagctcactttccaatgaggaatctgatc---tgttgtgttt-ctctaaggtgt
KIV2-1(-)5099bp       cctcgtgagctcactttccaatgaggaatctgatc---tgttgtgttt-ctctaaggtgt
KIV2_6(-)5104bp       cctcgtgagctcactttccaatgaggaatctgatc---tgttgtgttt-ctctaaggtgt
                      * ******** ****** ** **** *      *    **** *****     * * * *

KIV2B_3(-)5101bp      CAGGTGAAATATTTCCTAGAACTTACTACAGTTCTAGATTGGTAGGAATCTGTAGGTTTG
KIV2_5(-)5098bp       caggtgaaatatttccaagaacttactacagttctagaatgggaggaatctgttgctttg
KIV2_2(-)5096bp       caggtgaaatatttccaagaacttactacagttctagaatgggaggaatctgttgctttg
KIV2_4(-)5096bp       caggtgaaatatttccaagaacttactacagttctagaatgggaggaatctgttgctttg
KIV2-1(-)5099bp       caggtgaaatatttccaagaacttactacagttctagaatgggaggaatctgttgctttg
KIV2_6(-)5104bp       caggtgaaatatttccaagaacttactacagttctagaatgggaggaatctgttgctttg
                      **************** ********************* *** ********** * ****
```

```
KIV2B_3(-)5101bp      CTGTATGTTTTTTGGTTGGTTTTCTCCCATCCATCTGCCTACAGGTAAGGGAAAGATAAC
KIV2_5(-)5098bp       gtgtttgtttgttggtcggttttctcacatccatctgcctatggataaggaaaagagaac
KIV2_2(-)5096bp       gtgtttgtttgttggtcggttttctcacatccatctgcctatggataaggaaaagagaac
KIV2_4(-)5096bp       gtgtttgtttgttggtcggttttctcacatccatctgcctatggataaggaaaagagaac
KIV2-1(-)5099bp       gtgtttgtttgttggtcggttttctcacatccatctgcctatggataaggaaaagagaac
KIV2_6(-)5104bp       gtgtttgtttgttggtcggttttctcacatccatctgcctatggataaggaaaagagaac
                       *** ***** ***** ********* **************  * ***** ***** ***

KIV2B_3(-)5101bp      GTTCGTAATTCTCATAGACTCCTTTCTGGTTGTGTCATAAATGGCTTCACATATTTCGTT
KIV2_5(-)5098bp       ggtcgtaattctcatagactcctttctggttgtgtcacaaatggcttcacatgtttctct
KIV2_2(-)5096bp       ggtcgtaattctcatagactcctttctggttgtgtcacaaatggcttcacatgtttctct
KIV2_4(-)5096bp       ggtcgtaattctcatagactcctttctggttgtgtcacaaatggcttcacatgtttctct
KIV2-1(-)5099bp       ggtcgtaattctcatagactcctttctggttgtgtcacaaatggcttcacatgtttctct
KIV2_6(-)5104bp       ggtcgtaattctcatagactcctttctggttgtgtcacaaatggcttcacatgtttctct
                      * *********************************** ************** ****  *

KIV2B_3(-)5101bp      ATTCTCAGAGATACTCAGTTT-ATTTCTTGTGTTTTCATTTCAGCACCGACTGAGCAGAG
KIV2_5(-)5098bp       atgctcagagatactcagcttgatttcccgtgttttcatttcagcaccgactgagcaaag
KIV2_2(-)5096bp       atgctcagagatactcagcttgatttcccgtgttttcatttcagcaccgactgagcaaag
KIV2_4(-)5096bp       atgctcagagatactcagcttgatttcccgtgttttcatttcagcaccgactgagcaaag
KIV2-1(-)5099bp       atgctcagagatactcagcttgatttcccgtgttttcatttcagcaccgactgagcaaag
KIV2_6(-)5104bp       atgctcagagatactcagcttgatttcccgtgttttcatttcagcaccgactgagcaaag
                      ** *************** ** *****  **************************** **
```

```
KIV2B_3(-)5101bp      GCCTGGGGTGCAGGAGTGCTACCACGGTAATGGACAGAGTTATCGAGGCACATACTCCAC
KIV2_5(-)5098bp       gcctggggtgcaggagtgctaccatggtaatggacagagttatcgaggcacatactccac
KIV2_2(-)5096bp       gcctggggtgcaggagtgctaccatggtaatggacagagttatcgaggcacatactccac
KIV2_4(-)5096bp       gcctggggtgcaggagtgctaccatggtaatggacagagttatcgaggcacatactccac
KIV2-1(-)5099bp       gcctggggtgcaggagtgctaccatggtaatggacagagttatcgaggcacatactccac
KIV2_6(-)5104bp       gcctggggtgcaggagtgctaccatggtaatggacagagttatcgaggcacatactccac
                      ************************ ***********************************

KIV2B_3(-)5101bp      CACTGTCACTGGAAGAACCTGCCAAGCTTGGTCATCTATGACACCACACTCGCATAGTCG
KIV2_5(-)5098bp       cactgtcacaggaagaacctgccaagcttggtcatctatgacaccacactcgcatagtcg
KIV2_2(-)5096bp       cactgtcacaggaagaacctgccaagcttggtcatctatgacaccacactcgcatagtcg
KIV2_4(-)5096bp       cactgtcacaggaagaacctgccaagcttggtcatctatgacaccacactcgcatagtcg
KIV2-1(-)5099bp       cactgtcacaggaagaacctgccaagcttggtcatctatgacaccacactcgcatagtcg
KIV2_6(-)5104bp       cactgtcacaggaagaacctgccaagcttggtcatctatgacaccacactcgcatagtcg
                      ********* **************************************************

KIV2B_3(-)5101bp      GACCCCAGAATACTACCCAAATGCGTATGTCTTTGTTCTTTACCATAAGAGAAGAAAGGG
KIV2_5(-)5098bp       gaccccagaatactacccaaatgcgtatgtctttgttctttaccataagagaagaaaggg
KIV2_2(-)5096bp       gaccccagaatactacccaaatgcgtatgtctttgttctttaccataagagaagaaaggg
KIV2_4(-)5096bp       gaccccagaatactacccaaatgcgtatgtctttgttctttaccataagagaagaaaggg
KIV2-1(-)5099bp       gaccccagaatactacccaaatgcgtatgtctttgttctttaccataagagaagaaaggg
KIV2_6(-)5104bp       gaccccagaatactacccaaatgcgtatgtctttgttctttaccataagagaagaaaggg
                      ************************************************************

KIV2B_3(-)5101bp      CCAAGTGAAGTTTCTGTTACAAGAGATGTGTCTCAAGCTGAGTTCTCCGAACTCAACTTG
KIV2_5(-)5098bp       ccaagtgaagtttctgttacaagagatgtgtctcaagctgagttctccgaactcaacttg
KIV2_2(-)5096bp       ccaagtgaagtttctgttacaagagatgtgtctcaagctgagttctccgaactcaacttg
KIV2_4(-)5096bp       ccaagtgaagtttctgttacaagagatgtgtctcaagctgagttctccgaactcaacttg
KIV2-1(-)5099bp       ccaagtgaagtttctgttacaagagatgtgtctcaagctgagttctccgaactcaacttg
KIV2_6(-)5104bp       ccaagtgaagtttctgttacaagagatgtgtctcaagctgagttctccgaactcaacttg
                      ************************************************************
```

```
KIV2B_3(-)5101bp      TGACAGATGCAGATGGCGTAGCAAAATGTCTCAGGATGATTGCCTTGGAGCTAAGGGTCT
KIV2_5(-)5098bp       tgacagatgcagatggcgtagcaaaatgtctcaggatgattgccttggagctaagggtct
KIV2_2(-)5096bp       tgacagatgcagatggcgtagcaaaatgtctcaggatgattgccttggagctaagggtct
KIV2_4(-)5096bp       tgacagatgcagatggcgtagcaaaatgtctcaggatgattgccttggagctaagggtct
KIV2-1(-)5099bp       tgacagatgcagatggcgtagcaaaatgtctcaggatgattgccttggagctaagggtct
KIV2_6(-)5104bp       tgacagatgcagatggcgtagcaaaatgtctcaggatgattgccttggagctaagggtct
                      ************************************************************

KIV2B_3(-)5101bp      GAGAGAAGGGAAATGTTAAGCTCCCTCTCCTTCCTCCTAGTTCTATTGAGCAGAAGGGAA
KIV2_5(-)5098bp       gagagaagggaaatgttaagctccctctccttcctcctagttctattgagcagaagggaa
KIV2_2(-)5096bp       gagagaagggaaatgttaagctccctctccttcctcctagttctattgagcagaagggaa
KIV2_4(-)5096bp       gagagaagggaaatgttaagctccctctccttcctcctagttctattgagcagaagggaa
KIV2-1(-)5099bp       gagagaagggaaatgttaagctccctctccttcctcctagttctattgagcagaagggaa
KIV2_6(-)5104bp       gagagaagggaaatgttaagctccctctccttcctcctagttctattgagcagaagggaa
                      ************************************************************

KIV2B_3(-)5101bp      ATCTGGAGGTGAGGAGATCACATTATGAAGAAAGTCAGAATGACAAAGGACCAGACACTT
KIV2_5(-)5098bp       atctggaggtgagaagatcacattatgaagaaagtcagaatgacaaaggaccagacactt
KIV2_2(-)5096bp       atctggaggtgaggagatcacattatgaagaaagtcagaatgacaaaggaccagacactt
KIV2_4(-)5096bp       atctggaggtgaggagatcacattatgaagaaagtcagaatgacaaaggaccagacactt
KIV2-1(-)5099bp       atctggaggtgaggagatcacattatgaagaaagtcagaatgacaaaggaccagacactt
KIV2_6(-)5104bp       atctggaggtgaggagatcacattatgaagaaagtcagaatgacaaaggaccagacactt
                      ************* **********************************************
```

```
KIV2B_3(-)5101bp      AGATTACCCTTCCACAACACCAACTAAACGTCAATGGAGACTTTCCAGTTGGAATTCCGT
KIV2_5(-)5098bp       agattacccttccacaacaccaactaaacgtcaatggagactttccagttggaattccgt
KIV2_2(-)5096bp       agattacccttccacaacaccaactaaacgtcaatggagactttccagttggaattccgt
KIV2_4(-)5096bp       agattacccttccacaacaccaactaaacgtcaatggagactttccagttggaattccgt
KIV2-1(-)5099bp       agattacccttccacaacaccaactaaacgtcaatggagactttccagttggaattccgt
KIV2_6(-)5104bp       agattacccttccacaacaccaactaaacgtcaatggagactttccagttggaattccgt
                      ************************************************************

KIV2B_3(-)5101bp      TATTCTGGCTTCCACTTCCTGAAGGGAAGGTTGCGTTTGCCTTTTCTCTCTGGGTTCAAG
KIV2_5(-)5098bp       tattctggcttccacttcctgaagggaaggttgcgtttgccttttctctctgggttcaag
KIV2_2(-)5096bp       tattctggcttccacttcctgaagggaaggttgcgtttgccttttctctctgggttcaag
KIV2_4(-)5096bp       tattctggcttccacttcctgaagggaaggttgcgtttgccttttctctctgggttcaag
KIV2-1(-)5099bp       tattctggcttccacttcctgaagggaaggttgcgtttgccttttctctctgggttcaag
KIV2_6(-)5104bp       tattctggcttccacttcctgaagggaaggttgcgtttgccttttctctctgggttcaag
                      ************************************************************

KIV2B_3(-)5101bp      AGGAAAGAATAGGTGCTTATTTATGGACAGGTGAATTGATCTGTTTCTATATCTACGTAT
KIV2_5(-)5098bp       aggaaagaataggtgcttatttatggacaggtgaattgatctgtttctatatctacgtat
KIV2_2(-)5096bp       aggaaagaataggtgcttatttatggacaggtgaattgatctgtttctatatctacgtat
KIV2_4(-)5096bp       aggaaagaataggtgcttatttatggacaggtgaattgatctgtttctatatctacgtat
KIV2-1(-)5099bp       aggaaagaataggtgcttatttatggacaggtgaattgatctgtttctatatctacgtat
KIV2_6(-)5104bp       aggaaagaataggtgcttatttatggacaggtgaattgatctgtttctatatctacgtat
                      ************************************************************

KIV2B_3(-)5101bp      ATTCCGATTGTCAGAAAAACACTCGTTCCTAAGTACCAGTGGCCTGAAGGGATACAGGTT
KIV2_5(-)5098bp       attccgattgtcagaaaaacactcgttcctaagtaccagtggcctgaagggatacaggtt
KIV2_2(-)5096bp       attccgattgtcagaaaaacactcgttcctaagtaccagtggcctgaagggatacaggtt
KIV2_4(-)5096bp       attccgattgtcagaaaaacactcgttcctaagtaccagtggcctgaagggatacaggtt
KIV2-1(-)5099bp       attccgattgtcagaaaaacactcgttcctaagtaccagtggcctgaagggatacaggtt
KIV2_6(-)5104bp       attccgattgtcagaaaaacactcgttcctaagtaccagtggcctgaagggatacaggtt
                      ************************************************************
```

```
KIV2B_3(-)5101bp      CCCAGCAAGAGAAGATCCAAGGAAGGAAGGCAGATGAGAGCCAGCACAGAGAGGGATGCT
KIV2_5(-)5098bp       cccagcaagagaagatccaaggaaggaaggcagatgagagtcagcacagagagggatgct
KIV2_2(-)5096bp       cccagcaagagaagatccaaggaaggaaggcagatgagagtcagcacagagagggatgct
KIV2_4(-)5096bp       cccagcaagagaagatccaaggaaggaaggcagatgagagtcagcacagagagggatgct
KIV2-1(-)5099bp       cccagcaagagaagatccaaggaaggaaggcagatgagagtcagcacagagagggatgct
KIV2_6(-)5104bp       cccagcaagagaagatccaaggaaggaaggcagatgagagtcagcacagagagggatgct
                      **************************************** *******************

KIV2B_3(-)5101bp      GAAAAGTAAAAGGGATGGGTGGATGGAGAGAAGCCCGGGTCTGACCACCCAATGGCCAAT
KIV2_5(-)5098bp       gaaaagtaaaagggatgggtggatggagagaagcccgggtctgaccacccaatggccaat
KIV2_2(-)5096bp       gaaaagtaaaagggatgggtggatggagagaagcccgggtctgaccacccaatggccaat
KIV2_4(-)5096bp       gaaaagtaaaagggatgggtggatggagagaagcccgggtctgaccacccaatggccaat
KIV2-1(-)5099bp       gaaaagtaaaagggatgggtggatggagagaagcccgggtctgaccacccaatggccaat
KIV2_6(-)5104bp       gaaaagtaaaagggatgggtggatggagagaagcccgggtctgaccacccaatggccaat
                      ************************************************************

KIV2B_3(-)5101bp      ATTTTGGCCACAAGCGACTACCAGAGACATGGAAAAATGGTTTCTACATGTGGGACAACA
KIV2_5(-)5098bp       attttggccacaagcgactaccagagacatggaaaaatggtttctacatgtgggacaaca
KIV2_2(-)5096bp       attttggccacaagcgactaccagagacatggaaaaatggtttctacatgtgggacaaca
KIV2_4(-)5096bp       attttggccacaagcgactaccagagacatggaaaaatggtttctacatgtgggacaaca
KIV2-1(-)5099bp       attttggccacaagcgactaccagagacatggaaaaatggtttctacatgtgggacaaca
KIV2_6(-)5104bp       attttggccacaagcgactaccagagacatggaaaaatggtttctacatgtgggacaaca
                      ************************************************************

KIV2B_3(-)5101bp      GATGGTAGAGGACCTAGAGAATTGAGAGAGGGGCAATGATGGGCTCCACTCCGCAGATGC
KIV2_5(-)5098bp       gatggtagaggacctagagaattgagagaggggcaatgatgggctccactccgcagatgc
KIV2_2(-)5096bp       gatggtagaggacctagagaattgagagaggggcaatgatgggctccactccgcagatgc
KIV2_4(-)5096bp       gatggtagaggacctagagaattgagagaggggcaatgatgggctccactccgcagatgc
KIV2-1(-)5099bp       gatggtagaggacctagagaattgagagaggggcaatgatgggctccactccgcagatgc
KIV2_6(-)5104bp       gatggtagaggacctagagaattgagagaggggcaatgatgggctccactccgcagatgc
                      ************************************************************
```

```
KIV2B_3(-)5101bp      CTTGGCTTTCTTCCTGGATACCCTTCCTGCACTGAATAGCAAGGAGATGGAGCCCAAGCA
KIV2_5(-)5098bp       cttggctttcttcctggatacccttcctgcactgaatagcaaggagatggagcccaagca
KIV2_2(-)5096bp       cttggctttcttcctggatacccttcctgcactgaatagcaaggagatggagcccaagca
KIV2_4(-)5096bp       cttggctttcttcctggatacccttcctgcactgaatagcaaggagatggagcccaagca
KIV2-1(-)5099bp       cttggctttcttcctggatacccttcctgcactgaatagcaaggagatggagcccaagca
KIV2_6(-)5104bp       cttggctttcttcctggatacccttcctgcactgaatagcaaggagatggagcccaagca
                      ************************************************************

KIV2B_3(-)5101bp      GACTGTAGCCATCTTGCTGAATGGAGGAGAGGGATTGGAGTTTGGGATGACTGTGGTAGC
KIV2_5(-)5098bp       gactgtagccatcttgctgaatggaggagagggattggagtttgggatgactgtggtagc
KIV2_2(-)5096bp       gactgtagccatcttgctgaatggaggagagggattggagtttgggatgactgtggtagc
KIV2_4(-)5096bp       gactgtagccatcttgctgaatggaggagagggattggagtttgggatgactgtggtagc
KIV2-1(-)5099bp       gactgtagccatcttgctgaatggaggagagggattggagtttgggatgactgtggtagc
KIV2_6(-)5104bp       gactgtagccatcttgctgaatggaggagagggattggagtttgggatgactgtggtagc
                      ************************************************************

KIV2B_3(-)5101bp      TGAAATTTTTCTAGGTCTGCTAGAAATAAGAACTGGTTTGT--GGAGGAAAAGAGCTCTA
KIV2_5(-)5098bp       tgaaatttttctaggtctgctagaaataagaactggtttgtgtggaggaaaagagctcta
KIV2_2(-)5096bp       tgaaatttttctaggtctgctagaaataagaactggtttgt--ggaggaaaagagctcta
KIV2_4(-)5096bp       tgaaatttttctaggtctgctagaaataagaactggtttgt--ggaggaaaagagctcta
KIV2-1(-)5099bp       tgaaatttttctaggtctgctagaaataagaactggtttgtgtggaggaaaagagctcta
KIV2_6(-)5104bp       tgaaatttttctaggtctgctagaaataagaactggtttgtgtggaggaaaagagctcta
                      *****************************************  *****************
```

```
KIV2B_3(-)5101bp      CAAATACGCATAGAAGTCTCCTCCAGTCGTTGGCCTGACATGACGCTGCCTGTGCACAGG
KIV2_5(-)5098bp       caaatacgcatagaagtctcctccagtcgttggcctgacatgacgctgcctgtgcacagg
KIV2_2(-)5096bp       caaatacgcatagaagtctcctccagtcgttggcctgacatgacgctgcctgtgcacagg
KIV2_4(-)5096bp       caaatacgcatagaagtctcctccagtcgttggcctgacatgacgctgcctgtgcacagg
KIV2-1(-)5099bp       caaatacgcatagaagtctcctccagtcgttggcctgacatgacgctgcctgtgcacagg
KIV2_6(-)5104bp       caaatacgcatagaagtctcctccagtcgttggcctgacatgacgctgcctgtgcacagg
                      ************************************************************

KIV2B_3(-)5101bp      AAATGGTTCCACGAGAAAGTGTGGCAAAGAACATTTACTGAGAAACAGCAAGTACAAGAG
KIV2_5(-)5098bp       aaatggttccacgagaaagtgtggcaaagaacatttactgagaaacagcaagtacaagag
KIV2_2(-)5096bp       aaatggttccacgagaaagtgtggcaaagaacatttactgagaaacagcaagtacaagag
KIV2_4(-)5096bp       aaatggttccacgagaaagtgtggcaaagaacatttactgagaaacagcaagtacaagag
KIV2-1(-)5099bp       aaatggttccacgagaaagtgtggcaaagaacatttactgagaaacagcaagtacaagag
KIV2_6(-)5104bp       aaatggttccacgagaaagtgtggcaaagaacatttactgagaaacagcaagtacaagag
                      ************************************************************

KIV2B_3(-)5101bp      CACAGGAAGCTCAATAAAGAAGAGAGAGATCACATAGCACTCTGGGATACTGGAGTTCTT
KIV2_5(-)5098bp       cacaggaagctcaataaagaagagagagatcacatagcactctgggatactggagttctt
KIV2_2(-)5096bp       cacaggaagctcaataaagaagagagagatcacatagcactctgggatactggagttctt
KIV2_4(-)5096bp       cacaggaagctcaataaagaagagagagatcacatagcactctgggatactggagttctt
KIV2-1(-)5099bp       cacaggaagctcaataaagaagagagagatcacatagcactctgggatactggagttctt
KIV2_6(-)5104bp       cacaggaagctcaataaagaagagagagatcacatagcactctgggatactggagttctt
                      ************************************************************

KIV2B_3(-)5101bp      CCCAGCTAGACCAGAGAGTCCTCACGGAGCACATTGCCAATTCAGTGGAGACCCCAGAAC
KIV2_5(-)5098bp       cccagctagaccagagagtcctcacggagcacattgccaattcagtggagaccccagaac
KIV2_2(-)5096bp       cccagctagaccagagagtcctcacggagcacattgccaattcagtggagaccccagaac
KIV2_4(-)5096bp       cccagctagaccagagagtcctcacggagcacattgccaattcagtggagaccccagaac
KIV2-1(-)5099bp       cccagctagaccagagagtcctcacggagcacattgccaattcagtggagaccccagaac
KIV2_6(-)5104bp       cccagctagaccagagagtcctcacggagcacattgccaattcagtggagaccccagaac
                      ************************************************************
```

```
KIV2B_3(-)5101bp      AGCCGTAATTTAAAGGTACACTTAGTATATTACTAGAATAAAGTCAGCTGCAGACAACCC
KIV2_5(-)5098bp       agccgtaatttaaaggtacacttagtatattactagaataaagtcagctgcagacaaccc
KIV2_2(-)5096bp       agccgtaatttaaaggtacacttagtatattactagaataaagtcagctgcagacaaccc
KIV2_4(-)5096bp       agccgtaatttaaaggtacacttagtatattactagaataaagtcagctgcagacaaccc
KIV2-1(-)5099bp       agccgtaatttaaaggtacacttagtatattactagaataaagtcagctgcagacaaccc
KIV2_6(-)5104bp       agccgtaatttaaaggtacacttagaatattactagaataaagtcagctgcagacaaccc
                      ************************ ***********************************

KIV2B_3(-)5101bp      CTTGCACAGCTGGAAAGCAAGTGTCCAAGCATCAAATCGGTTTCCAATCAATGAAGTGCC
KIV2_5(-)5098bp       cttgcacagctggaaagcaagtgtccaagcatcaaatcggtttccaatcaatgaagtgcc
KIV2_2(-)5096bp       cttgcacagctggaaagcaagtgtccaagcatcaaatcggtttccaatcaatgaagtgcc
KIV2_4(-)5096bp       cttgcacagctggaaagcaagtgtccaagcatcaaatcggtttccaatcaatgaagtgcc
KIV2-1(-)5099bp       cttgcacagctggaaagcaagtgtccaagcatcaaatcggtttccaatcaatgaagtgcc
KIV2_6(-)5104bp       cttgcacagctggaaagcaagtgtccaagcatcaaatcggtttccaatcaatgaagtgcc
                      ************************************************************

KIV2B_3(-)5101bp      TGTGGGAGGAAATCTCAACTCTCTTTAGAAGTAAACAACAAAGTCGATTGCCTCAGCTAT
KIV2_5(-)5098bp       tgtgagaggaaatctcaactctctttagaagtaaacaacaaagtcgattgcctcagctat
KIV2_2(-)5096bp       tgtgagaggaaatctcaactctctttagaagtaaacaacaaagtcgattgcctcagctat
KIV2_4(-)5096bp       tgtgagaggaaatctcaactctctttagaagtaaacaacaaagtcgattgcctcagctat
KIV2-1(-)5099bp       tgtgagaggaaatctcaactctctttagaagtaaacaacaaagtcgattgcctcagctat
KIV2_6(-)5104bp       tgtgagaggaaatctcaactctctttagaagtaaacaacaaagtcgattgcctcagctat
                      **** *******************************************************
```

```
KIV2B_3(-)5101bp      GCGGTATCCGCAGAGTGAGTCCTAAATTTAAAATCTGACTACATGTAGAAAAGCGTTTCG
KIV2_5(-)5098bp       gcggtatccgcagagtgagtcctaaatttaaaatctgactacatgtagaaaagcgtttcg
KIV2_2(-)5096bp       gcggtatccgcagagtgagtcctaaatttaaaatctgactacatgtagaaaagcgtttcg
KIV2_4(-)5096bp       gcggtatccgcagagtgagtcctaaatttaaaatctgactacatgtagaaaagcgtttcg
KIV2-1(-)5099bp       gcggtatccgcagagtgagtcctaaatttaaaatctgactacatgtagaaaagcgtttcg
KIV2_6(-)5104bp       gcggtatccgcagagtgagtcctaaatttaaaatctgactacatgtagaaaagcgtttcg
                      ************************************************************

KIV2B_3(-)5101bp      TGTGACCCATGACCAGGAAATAAATCGGGTAATACAAACAGGCTCAGGAATGAGAGAAAT
KIV2_5(-)5098bp       tgtgacccatgaccaggaaataaatcgggtaatacaaacaggctcaggaatgagagaaat
KIV2_2(-)5096bp       tgtgacccatgaccaggaaataaatcgggtaatacaaacaggctcaggaatgagagaaat
KIV2_4(-)5096bp       tgtgacccatgaccaggaaataaatcgggtaatacaaacaggctcaggaatgagagaaat
KIV2-1(-)5099bp       tgtgacccatgaccaggaaataaatcgggtaatacaaacaggctcaggaatgagagaaat
KIV2_6(-)5104bp       tgtgacccatgaccaggaaataaatcgggtaatacaaacaggctcaggaatgagagaaat
                      ************************************************************

KIV2B_3(-)5101bp      GATTAGAATTGCGTGAAAATTTGACATATCAGTATGATAACTGATTTCAAATATTTAAAA
KIV2_5(-)5098bp       gattagaattgcgtgaaaatttgacatatcagtatgataactgatttcaaatatttaaaa
KIV2_2(-)5096bp       gattagaattgcgtgaaaatttgacatatcagtatgataactgatttcaaatatttaaaa
KIV2_4(-)5096bp       gattagaattgcgtgaaaatttgaaatatcagtatgataactgatttcaaatatttaaaa
KIV2-1(-)5099bp       gattagaattgcgtgaaaatttgacatatcagtatgataactgatttcaaatatttaaaa
KIV2_6(-)5104bp       gattagaattgcgtgaaaatttgacatatcagtatgataactgatttcaaatatttaaaa
                      ************************ ***********************************

KIV2B_3(-)5101bp      AAACAACATGCAAGAAAGCAGATATCATATCAAGAGAAATTAACAGTACAGAATAGCCAA
KIV2_5(-)5098bp       aaacaacatgcaagaaagcagatatcatatcaagagaaattaacagtacagaatagccaa
KIV2_2(-)5096bp       aaacaacatgcaagaaagcagatatcatatcaagagaaattaacagtacagaatagccaa
KIV2_4(-)5096bp       aaacaacatgcaagaaagcagatatcatatcaagagaaattaacagtacagaatagccaa
KIV2-1(-)5099bp       aaacaacatgcaagaaagcagatatcatatcaagagaaattaacagtacagaatagccaa
KIV2_6(-)5104bp       aaacaacatgcaagaaagcagatatcatatcaagagaaattaacagtacagaatagccaa
                      ************************************************************
```

```
KIV2B_3(-)5101bp     ATTAAATTAAAGAGCTAGTATAAAAAAAGTATGTCTTAATTGAAAAAAATTACTGTATGG
KIV2_5(-)5098bp      attaaattaaagaggtagtataaaaaaagtatgtcttaattgaaaaaaattactgtatgg
KIV2_2(-)5096bp      attaaattaaagagctagtataaaaaaagtatgtcttaattgaaaaaaattactgtatgg
KIV2_4(-)5096bp      attaaattaaagagctagtataaaaaaagtatgtcttaattgaaaaaaattactgtatgg
KIV2-1(-)5099bp      attaaattaaagaggtagtataaaaaaagtatgtcttaattgaaaaaaattactgtatgg
KIV2_6(-)5104bp      attaaattaaagagctagtataaaaaaagtatgtcttaattgaaaaaaattactgtatgg
                     ************** *********************************************

KIV2B_3(-)5101bp     CCGGCTGATCAAATTAGACGTTTCAGAGGAAAACATTACCCAACACACAATTCTAGAGAA
KIV2_5(-)5098bp      ccggctgatcaatttagacgtttcagaggaaaacattacccaacacacaattctagagaa
KIV2_2(-)5096bp      ccggctgatcaatttagacgtttcagaggaaaacattacccaacacacaattctagagaa
KIV2_4(-)5096bp      ccggctgatcaatttagacgtttcagaggaaaacattacccaacacacaattctagagaa
KIV2-1(-)5099bp      ccggctgatcaatttagacgtttcagaggaaaacattacccaacacacaattctagagaa
KIV2_6(-)5104bp      ccggctgatcaaattagacgtttcagaggaaaacattacccaacacacaatttttagagaa
                     ************ *************************************** *******

KIV2B_3(-)5101bp     CCTACAGAATGAGCTACACACACACACACACACA----CACACACACACACTGAAAACAC
KIV2_5(-)5098bp      cctacagaatgagctacacacacacacacaca------cacacacacaaactgaaaacac
KIV2_2(-)5096bp      cctacagaatgagctacacacacacacacaca------cacacacacaaactgaaaacac
KIV2_4(-)5096bp      cctacagaatgagctacacacacacacacaca------cacacacacaaactgaaaacac
KIV2-1(-)5099bp      cctacagaatgagctacacacacacacacacaca----cacacacacacactgaaaacac
KIV2_6(-)5104bp      cctacagaatgagctacacacacacacacacacacacacacacacacaaactgaaaacac
                     ********************************      ********** ***********
```

```
KIV2B_3(-)5101bp      ACCCATACTCACACACACGCAGAAACTCACAAGTTCTAACACACACAGACACGCGCACCC
KIV2_5(-)5098bp       acccatactcacacacacgcagaaactcacaagttctaacacacacagacacgcgcaccc
KIV2_2(-)5096bp       acccatactcacacacacgcagaaactcacaagttctaacacacacagacacgcgcaccc
KIV2_4(-)5096bp       acccatactcacacacacgcagaaactcacaagttctaacacacacagacacgcgcaccc
KIV2-1(-)5099bp       acccatactcacacacacgcagaaactcacaagttctaacacacacagacacgcgcaccc
KIV2_6(-)5104bp       acccatactcacacacacgcagaaactcacaagttctaacacacacagacacgcgcaccc
                      ************************************************************

KIV2B_3(-)5101bp      CTGAAGAAACAGTGAAATATAAAATTAAGCGAGCCTCACAGACATGTAGGAAAATATGAA
KIV2_5(-)5098bp       ctgaagaaacagtgaaatataaaattaagcgagcctcacagacatgtaggaaaatatgaa
KIV2_2(-)5096bp       ctgaagaaacagtgaaatataaaattaagcgagcctcacagacatgtaggaaaatatgaa
KIV2_4(-)5096bp       ctgaagaaacagtgaaatataaaattaagcgagcctcacagacatgtaggaaaatatgaa
KIV2-1(-)5099bp       ctgaagaaacagtgaaatataaaattaagcgagcctcacagacatgtaggaaaatatgaa
KIV2_6(-)5104bp       ctgaagaaacagtgaaatataaaattaagcgagcctcacagacatgtaggaaaatatgaa
                      ************************************************************

KIV2B_3(-)5101bp      AAGATTTCCTGCATGTGGGAAGCAAGTCACAGTAAAGAGCAAGGGAGTTTGGAATAGAAA
KIV2_5(-)5098bp       aagatttcctgcatgtgggaagcaagtcacagtaaagagcaagggagtttggaatagaaa
KIV2_2(-)5096bp       aagatttcctgcatgtgggaagcaagtcacagtaaagagcaagggagtttggaatagaaa
KIV2_4(-)5096bp       aagatttcctgcatgtgggaagcaagtcacagtaaagagcaagggagtttggaatagaaa
KIV2-1(-)5099bp       aagatttcctgcatgtgggaagcaagtcacagtaaagagcaagggagtttataatagaaa
KIV2_6(-)5104bp       aagatttcctgcatgtgggaagcaagtcacagtaaagagcaagggagtttataatagaaa
                      **************************************************  ********

KIV2B_3(-)5101bp      CAAATACCGGAATCAAGGATGGCTGATAACTTTTCAATTACGAAGAACATTAAAAAAAAT
KIV2_5(-)5098bp       caaataccggaatcaaggatggctgataacttttcaattacgaagaacattaaaaaaaat
KIV2_2(-)5096bp       caaataccggaatcaaggatggctgataacttttcaattacgaagaacattaaaaaaaat
KIV2_4(-)5096bp       caaataccagaatcaaggatggctgataacttttcaattacgaagaacattaaaaaaaat
KIV2-1(-)5099bp       caaataccagaatcaaggatggctgataacttttcaattacgaagaacattaaaaaaaat
KIV2_6(-)5104bp       caaataccagaatcaaggatggctgataacttttcaattacgaagaacattaaaaaaaat
                      ******** ***************************************************
```

```
KIV2B_3(-)5101bp      CACAGAATCGTGAAACTCAAGGGATCATATAGGGAATTTCGGAAAAAAAACCCAACCTGT
KIV2_5(-)5098bp       cacagaatcgtgaaactcaagggatcacatagggaatttcggaaaaaaaacccaacctgt
KIV2_2(-)5096bp       cacagaatcgtgaaactcaagggatcacatagggaatttcggaaaaaaaacccaacctgt
KIV2_4(-)5096bp       cacagaatcgtgaaactcaagggatcacatagggaatttcggaaaaaaaacccaacctgt
KIV2-1(-)5099bp       cacagaatcgtgaaactcaagggatcatatagggaatttcggaaaaaaaacccaacctgt
KIV2_6(-)5104bp       cacagaatcgtgaaactcaagggatcatatagggaatttcggaaaaaaaacccaacctgt
                      *************************** ********************************

KIV2B_3(-)5101bp      ATGATGTACTTTTGTACATCACAGTTCGAAGGTAACAAGGCAAAGATATAATAAGAAGAA
KIV2_5(-)5098bp       atgatgtacttttgtacatcacagttcgaaggtaacaaggcaaagatataataagaagaa
KIV2_2(-)5096bp       atgatgtacttttgtacatcacagttcgaaggtaacaaggcaaagatataataagaagaa
KIV2_4(-)5096bp       atgatgtacttttgtacatcacagttcgaaggtaacaaggcaaagatataataagaagaa
KIV2-1(-)5099bp       atgatgtacttttgtacatcacagttcgaaggtaacaaggcaaagatgtaataagaagaa
KIV2_6(-)5104bp       atgatgtacttttgtacatcacagttcgaaggtaacaaggcaaagatgtaataagaagaa
                      *********************************************** ************

KIV2B_3(-)5101bp      ACCTGTCACGAGAAACTGGAGGAAAAAGAGCTGTGTCTTCCTACAAGTACACTGATACAA
KIV2_5(-)5098bp       acctgtcacgagaaactggaggaaaaagagctgtgtcttcctacaagtacactgatacaa
KIV2_2(-)5096bp       acctgtcacgagaaactggaggaaaaagagctgtgtcttcctacaagtacactgatacaa
KIV2_4(-)5096bp       acctgtcacgagaaactggaggaaaaagagctgtgtcttcctacaagtacactgatacaa
KIV2-1(-)5099bp       acctgtcacgagaaactggaggaaaaagagctgtgtcttcctacaagtacactgatacaa
KIV2_6(-)5104bp       acctgtcacgagaaactggaggaaaaagagctgtgtcttcctacaagtacactgatacaa
                      ************************************************************
```

```
KIV2B_3(-)5101bp      ATTGCCAATGTGTTCACCTCAGAAACACTGGAAGCCAGATACCAGGGAATATTGTTAAAA
KIV2_5(-)5098bp       attgccaatgtgttcacctcagaaacactggaagccagataccagggaatattgttaaaa
KIV2_2(-)5096bp       attgccaatgtgttcacctcagaaacactggaagccagataccagggaatattgttaaaa
KIV2_4(-)5096bp       attgccaatgtgttcacctcagaaacactggaagccagataccagggaatattgttaaaa
KIV2-1(-)5099bp       attgccaatgtgttcacctcagaaacactggaagccagataccagggaatattgttaaaa
KIV2_6(-)5104bp       attgccaatgtgttcacctcagaaacactggaagccagataccagggaatattgttaaaa
                      ************************************************************

KIV2B_3(-)5101bp      TGATAATCAGGAACAAAAAGAGATCAACCGGGAATGCTGAATCCAGCAATAAAATGCCTT
KIV2_5(-)5098bp       tgataatcaggaacaaaaagagatcaaccgggaatgctgaatccagcaataaaatgcctt
KIV2_2(-)5096bp       tgataatcaggaacaaaaagagatcaaccgggaatgctgaatccagcaataaaatgcctt
KIV2_4(-)5096bp       tgataatcaggaacaaaaagagatcaaccgggaatgctgaatccagcaataaaatgcctt
KIV2-1(-)5099bp       tgataatcaggaacaaaaagagatcaaccgggaatgctgaatccagcaataaaatgcctt
KIV2_6(-)5104bp       tgataatcaggaacaaaaagagatcaaccgggaatgctgaatccagcaataaaatgcctt
                      ************************************************************

KIV2B_3(-)5101bp      GAAGATCATCCATGTCGGATAAATGCATATTGTGCACTGCCCCAAAGAAAGAAACCGGAA
KIV2_5(-)5098bp       gaaggtcatccatgtcggataaatgcatattgtgcactgccccaaagaaagaaaccggaa
KIV2_2(-)5096bp       gaagatcatccatgtcggataaatgcatattgtgcactgccccaaagaaagaaaccggaa
KIV2_4(-)5096bp       gaagatcatccatgtcggataaatgcatattgtgcactgccccaaagaaagaaaccggaa
KIV2-1(-)5099bp       gaaggtcatccatgtcggataaatgcatattgtgcactgccccaaagaaagaaaccggaa
KIV2_6(-)5104bp       gaaggtcatccatgtcggataaatgcatattgtgcactgccccaaagaaagaaaccggaa
                      **** *******************************************************

KIV2B_3(-)5101bp      ACTGTCAGAATTGGAAATCAGCAGGCTTATGTAACAAGAGAGGTGACCCGAAGGAATTAG
KIV2_5(-)5098bp       actgtaagaattggaaatcagcaggcttatgtaacaagagaggtgacccgaaggaattag
KIV2_2(-)5096bp       actgtaagaattggaaatcagcaggcttatgtaacaagagaggtgacccgaaggaattag
KIV2_4(-)5096bp       actgtaagaattggaaatcagcaggcttatgtaacaagagaggtgacccgaaggaattag
KIV2-1(-)5099bp       actgtaagaattggaaatcagcaggcttatgtaacaagagaggtgacccgaaggaattag
KIV2_6(-)5104bp       actgtaagaattggaaatcagcaggcttatgtaacaagagaggtgacccgaaggaattag
                      ***** ******************************************************
```

```
KIV2B_3(-)5101bp      GTAGAAGAAGAATTGAACAAGAAAGGAACTTTCTGCAGCCCACGTAATGAAGAATCCAGC
KIV2_5(-)5098bp       gtagaagaagaattgaacaagaaaggaactttctgcagcccacgtaatgaagaatccagc
KIV2_2(-)5096bp       gtagaagaagaattgaacaagaaaggaactttctgcagcccacgtaatgaagaatccagc
KIV2_4(-)5096bp       gtagaagaagaattgaacaagaaaggaactttctgcagcccacgtaatgaagaatccagc
KIV2-1(-)5099bp       gtagaagaagaattgaacaagaaaggaactttctgcagcccacgtaatgaagaatccagc
KIV2_6(-)5104bp       gtagaagaagaattgaacaagaaaggaactttctgcagcccacgtaatgaagaatccagc
                      ************************************************************

KIV2B_3(-)5101bp      AATTGGCAAATGTAGATAGATGTAAATGCAAAATATTTTCTTGATCAAATTTCTATATCT
KIV2_5(-)5098bp       aattggcaaatgtagatagatgtaaatgcaaaatattttcttgatcaaatttctatatct
KIV2_2(-)5096bp       aattggcaaatgtagatagatgtaaatgcaaaatattttcttgatcaaatttctatatct
KIV2_4(-)5096bp       aattggcaaatgtagatagatgtaaatgcaaaatattttcttgatcaaatttctatatct
KIV2-1(-)5099bp       aattggcaaatgtagatagatgtaaatgcaaaatattttcttgatcaaatttctatatct
KIV2_6(-)5104bp       aattggcaaatgtagatagatgtaaatgcaaaatattttcttgatcaaatttctatatct
                      ************************************************************

KIV2B_3(-)5101bp      TTGTAAATGAGAGTTGACTACTTGAAACAAAATGATAGCAAGATATTTAACTTCAGCATA
KIV2_5(-)5098bp       ttgtaaatgagagttgactacttgaaacaaaatgatagcaagatatttaacttcagcata
KIV2_2(-)5096bp       ttgtaaatgagagttgactacttgaaacaaaatgatagcaagatatttaacttcagcata
KIV2_4(-)5096bp       ttgtaaatgagagttgactacttgaaacaaaatgatagcaagatatttaacttcagcata
KIV2-1(-)5099bp       ttgtaaatgagagttgactacttgaaacaaaatgatagcaagatatttaacttcagcata
KIV2_6(-)5104bp       ttgtaaatgagagttgactacttgaaacaaaatgatagcaagatatttaacttcagcata
                      ************************************************************
```

```
KIV2B_3(-)5101bp      TGTAGAGGTAAGAATTTGAAATGGTAGCATAAATCACGAAGGGATTAATTCGAAGTGTAC
KIV2_5(-)5098bp       tgtagaggtaagaatttgaaatggtagcataaatcacgaagggattaattcgaagtgtac
KIV2_2(-)5096bp       tgtagaggtaagaatttgaaatggtagcataaatcacgaagggattaattcgaagtgtac
KIV2_4(-)5096bp       tgtagaggtaagaatttgaaatggtagcataaatcacgaagggattaattcgaagtgtac
KIV2-1(-)5099bp       tgtagaggtaagaatttgaaatggtagcataaatcacgaagggattaattcgaagtgtac
KIV2_6(-)5104bp       tgtagaggtaagaatttgaaatggtagcataaatcacgaagggattaattcgaagtgtac
                      ************************************************************

KIV2B_3(-)5101bp      CGTTGTAAGTTTCTTTACCTCATGCACGATGGTGTGTCATATTAATAAAAGGGTACTGTG
KIV2_5(-)5098bp       cgttgtaagtttctttacctcatgcacgatggtgtgtcatattaataaaagggtactgtg
KIV2_2(-)5096bp       cgttgtaagtttctttacctcatgcacgatggtgtgtcatattaataaaagggtactgtg
KIV2_4(-)5096bp       cgttgtaagtttctttacctcatgcacgatggtgtgtcatattaataaaagggtactgtg
KIV2-1(-)5099bp       cgttgtaagtttctttacctcatgcacgatggtgtgtcatattaataaaagggtactgtg
KIV2_6(-)5104bp       cgttgtaagtttctttacctcatgcacgatggtgtgtcatattaataaaagggtactgtg
                      ************************************************************

KIV2B_3(-)5101bp      CGGGTTCGAAGGGATATTGCAAATCCTAGAGCAATCACAAAGGTTTGAACTCTGAGGTTT
KIV2_5(-)5098bp       cgggttcgaagggatattgcaaatcctagagcaatcacaaaggtttgaactctgaggttt
KIV2_2(-)5096bp       cgggttcgaagggatattgcaaatcctagagcaatcacaaaggtttgaactctgaggttt
KIV2_4(-)5096bp       cgggttcgaagggatattgcaaatcctagagcaatcacaaaggtttgaactctgaggttt
KIV2-1(-)5099bp       cgggttcgaagggatattgcaaatcctagagcaatcacaaaggtttgaactctgaggttt
KIV2_6(-)5104bp       cgggttcgaagggatattgcaaatcctagagcaatcacaaaggtttgaactctgaggttt
                      ************************************************************

KIV2B_3(-)5101bp      TTGGTATAATAAGAATAGTCCATGCATTCAAAAGAGGGAAGCCAAGGAAGAACTAGAAGT
KIV2_5(-)5098bp       ttggtataataagaatagtccatgcattcaaaagagggaagccaaggaagaactagaagt
KIV2_2(-)5096bp       ttggtataataagaatagtccatgcattcaaaagagggaagccaaggaagaactagaagt
KIV2_4(-)5096bp       ttggtataataagaatagtccatgcattcaaaagagggaagccaaggaagaactagaagt
KIV2-1(-)5099bp       ttggtataataagaatagtccatgcattcaaaagagggaagccaaggaagaactagaagt
KIV2_6(-)5104bp       ttggtataataagaatagtccatgcattcaaaagagggaagccaaggaagaactagaagt
                      ************************************************************
```

```
KIV2B_3(-)5101bp      CTTTCAAGAGCTCAGGCTCTTATACATCCAGTTGCTCATTGAACCAGCTTCCTGGAATGG
KIV2_5(-)5098bp       ctttcaagagctcaggctcttatacatccagttgctcattgaaccagcttcctggaatgg
KIV2_2(-)5096bp       ctttcaagagctcaggctcttatacatccagttgctcattgaaccagcttcctggaatgg
KIV2_4(-)5096bp       ctttcaagagctcaggctcttatacatccagttgctcattgaaccagcttcctggaatgg
KIV2-1(-)5099bp       ctttcaagagctcaggctcttatacatccagttgctcattgaaccagcttcctggaatgg
KIV2_6(-)5104bp       ctttcaagagctcaggctcttatacatccagttgctcattgaaccagcttcctggaatgg
                      ************************************************************

KIV2B_3(-)5101bp      AGGGTCTGGGGTTGAGACTAGGCCACAAGTCTAGAGTCTCTAGAGAGACAGTGTTGGAAC
KIV2_5(-)5098bp       agggtctggggttgagactaggccacaagtctagagtctctagagagacagtgttggaac
KIV2_2(-)5096bp       agggtctggggttgagactaggccacaagtctagagtctctagagagacagtgttggaac
KIV2_4(-)5096bp       agggtctggggttgagactaggccacaagtctagagtctctagagagacagtgttggaac
KIV2-1(-)5099bp       agggtctggggttgagactaggccacaagtctagagtctctagagagacagtgttggaac
KIV2_6(-)5104bp       agggtctggggttgagactaggccacaagtctagagtctctagagagacagtgttggaac
                      ************************************************************

KIV2B_3(-)5101bp      CCCATGGCCCATAATACATTTCCCATTTTCTCAGGCAGCCAGAGGTCATGAATGTGAGGA
KIV2_5(-)5098bp       cccatggcccataatacatttcccattttctcaggcagccagaggtcatgaatgtgagga
KIV2_2(-)5096bp       cccatggcccataatacatttcccattttctcaggcagccagaggtcatgaatgtgagga
KIV2_4(-)5096bp       cccatggcccataatacatttcccattttctcaggcagccagaggtcatgaatgtgagga
KIV2-1(-)5099bp       cccatggcccataatacatttcccattttctcaggcagccagaggtcatgaatgtgagga
KIV2_6(-)5104bp       cccatggcccataatacatttcccattttctcaggcagccagaggtcatgaatgtgagga
                      ************************************************************
```

```
KIV2B_3(-)5101bp      TACTGGGAGGTTGGAGCAACGTTCTTGGGAGGCATAAGGAAGAGCGAATGCTTCAAGATC
KIV2_5(-)5098bp       tactgggaggttggagcaacgttcttgggaggcataaggaagagcgaatgcttcaagatc
KIV2_2(-)5096bp       tactgggaggttggagcaacgttcttgggaggcataaggaagagcgaatgcttcaagatc
KIV2_4(-)5096bp       tactgggaggttggagcaacgttcttgggaggcataaggaagagcgaatgcttcaagatc
KIV2-1(-)5099bp       tactgggaggttggagcaacgttcttgggaggcataaggaagagcgaatgcttcaagatc
KIV2_6(-)5104bp       tactgggaggttggagcaacgttcttgggaggcataaggaagagcgaatgcttcaagatc
                      ************************************************************

KIV2B_3(-)5101bp      CCCGCAGCCCAAACTACTCGCCTGCTTTGCCCCCTAATGCATTTTTCTCTGCTGCTCCGT
KIV2_5(-)5098bp       cccgcagcccaaactactcgcctgctttgccccctaatgcatttttctctgctgctccgt
KIV2_2(-)5096bp       cccgcagcccaaactactcgcctgctttgccccctaatgcatttttctctgctgctccgt
KIV2_4(-)5096bp       cccgcagcccaaactactcgcctgctttgccccctaatgcatttttctctgctgctccgt
KIV2-1(-)5099bp       cccgcagcccaaactactcgcctgctttgccccctaatgcatttttctctgctgctccgt
KIV2_6(-)5104bp       cccgcagcccaaactactcgcctgctttgccccctaatgcatttttctctgctgctccgt
                      ************************************************************

KIV2B_3(-)5101bp      AGCTGTCCGACCTCTTCAGATCTCTTAGTCCACCCTGCCGTCTTCCTTTATGCCATGGGT
KIV2_5(-)5098bp       agctgtccgacctcttcagatctcttagtccaccctgccgtcttcctttatgccatgggt
KIV2_2(-)5096bp       agctgtccgacctcttcagatctcttagtccaccctgccgtcttcctttatgccatgggt
KIV2_4(-)5096bp       agctgtccgacctcttcagatctcttagtccaccctgccgtcttcctttatgccatgggt
KIV2-1(-)5099bp       agctgtccgacctcttcagatctcttagtccaccctgccgtcttcctttatgccatgggt
KIV2_6(-)5104bp       agctgtccgacctcttcagatctcttagtccaccctgccgtcttcctttatgccatgggt
                      ************************************************************

KIV2B_3(-)5101bp      CCCATTGTTCTTTCAACTCATCCCCCTTTCCCTCAGTCCCGGAGTAGCTGCGGCCAGCAG
KIV2_5(-)5098bp       cccactgttctttcaactcatccccctttccctcagtcccggagtagctgcggccagcag
KIV2_2(-)5096bp       cccactgttctttcaactcatccccctttccctcagtcccggagtagctgcggccagcag
KIV2_4(-)5096bp       cccactgttctttcaactcatccccctttccctcagtcccggagtagctgcggccagcag
KIV2-1(-)5099bp       cccactgttctttcaactcatccccctttccctcagtcccggagtagctgcggccagcag
KIV2_6(-)5104bp       cccactgttctttcaactcatccccctttccctcagtcccggagtagctgcggccagcag
                      **** *******************************************************
```

```
KIV2B_3(-)5101bp      AGGGTAGACTGAGAGCAGGAGAGAAGGACCTGCCTAGGAACCCCTTCTAGAGATACTGCA
KIV2_5(-)5098bp       agggtagactgagagcaggagagaaggacctgcctaggaaccccttctagagatactgca
KIV2_2(-)5096bp       agggtagactgagagcaggagagaaggacctgcctaggaaccccttctagagatactgca
KIV2_4(-)5096bp       agggtagactgagagcaggagagaaggacctgcctaggaaccccttctagagatactgca
KIV2-1(-)5099bp       agggtagactgagagcaggagagaaggacctgcctaggaaccccttctagagatactgca
KIV2_6(-)5104bp       agggtagactgagagcaggagagaaggacctgcctaggaaccccttctagagatactgca
                      ************************************************************

KIV2B_3(-)5101bp      TCCTGCCTGGGAGCAAGTTTTCCAGGGCAGCTTTGAGAAGTCTTGGAGAAACAAACCTAC
KIV2_5(-)5098bp       tcctgcctgggagcaagttttccagggcagctttgagaagtcttggagaaacaaacctac
KIV2_2(-)5096bp       tcctgcctgggagcaagttttccagggcagctttgagaagtcttggagaaacaaacctac
KIV2_4(-)5096bp       tcctgcctgggagcaagttttccagggcagctttgagaagtcttggagaaacaaacctac
KIV2-1(-)5099bp       tcctgcctgggagcaagttttccagggcagctttgagaagtcttggagaaacaaacctac
KIV2_6(-)5104bp       tcctgcctgggagcaagttttccagggcagctttgagaagtcttggagaaacaaacctac
                      ************************************************************

KIV2B_3(-)5101bp      TAAACCTGACAGACAGTAATACTATTTGCACAATGCTTTTCTGTGGGAAAGGTAGAGCCT
KIV2_5(-)5098bp       taaacctgacagacagtaatactatttgcacaatgcttttctgtgggaaaggtagagcct
KIV2_2(-)5096bp       taaacctgacagacagtaatactatttgcacaatgcttttctgtgggaaaggtagagcct
KIV2_4(-)5096bp       taaacctgacagacagtaatactatttgcacaatgcttttctgtgggaaaggtagagcct
KIV2-1(-)5099bp       taaacctgacagacagtaatactatttgcacaatgcttttctgtgggaaaggtagagcct
KIV2_6(-)5104bp       taaacctgacagacagtaatactatttgcacaatgcttttctgtgggaaaggtagagcct
                      ************************************************************
```

```
KIV2B_3(-)5101bp      TTTCACTACGTATTGAGTACATAGAGTGTGAGGGTTGACCTGGAACGGCTATCCTCCTGG
KIV2_5(-)5098bp       tttcactacgtattgagtacatagagtgtgagggttgacctggaacggctatcctcctgg
KIV2_2(-)5096bp       tttcactacgtattgagtacatagagtgtgagggttgacctggaacggctatcctcctgg
KIV2_4(-)5096bp       tttcactacgtattgagtacatagagtgtgagggttgacctggaacggctatcctcctgg
KIV2-1(-)5099bp       tttcactacgtattgagtacatagagtgtgagggttgacctggaacggctatcctcctgg
KIV2_6(-)5104bp       tttcactacgtattgagtacatagagtgtgagggttgacctggaacggctatcctcctgg
                      ************************************************************

KIV2B_3(-)5101bp      ATGACGTGCGTTTTCTGAAGAACTACATGTTCGTTGCAACTCCCACATTAGAATATGAAG
KIV2_5(-)5098bp       atgacgtgcgttttctgaagaactacatgttcgttgcaactcccacattagaatatgaag
KIV2_2(-)5096bp       atgacgtgtgttttctgaagaactacatgttcgttgcaactcccacattagaatatgaag
KIV2_4(-)5096bp       atgacgtgtgttttctgaagaactacatgttcgttgcaactcccacattagaatatgaag
KIV2-1(-)5099bp       atgacgtgtgttttctgaagaactacatgttcgttgcaactcccacattagaatatgaag
KIV2_6(-)5104bp       atgacgtgcgttttctgaagaactacatgttcgttgcaactcccacattagaatatgaag
                      ******** ***************************************************

KIV2B_3(-)5101bp      TCCTACCGAGAGAGATACGGAGACTAGACAGATACAGATGCATTTGCATGTGAATACACA
KIV2_5(-)5098bp       tcctaccgagagagatacggagactagacagatacagatgcatttgcatgtgaatacaca
KIV2_2(-)5096bp       tcctaccgagagagatacggagactagacagatacagatgcatttgcatgtgaatacaca
KIV2_4(-)5096bp       tcctaccgagagagatacggagactagacagatacagatgcatttgcatgtgaatacaca
KIV2-1(-)5099bp       tcctaccgagagagatacggagactagacagatacagatgcatttgcatgtgaatacaca
KIV2_6(-)5104bp       tcctaccgagagagatacggagactagacagatacagatgcatttgcatgtgaatacaca
                      ************************************************************
```

```
KIV2B_3(-)5101bp      ATCCCACAATACAGACGTCAAAACCCATACCAGTTATTCCAGAGAGATGGATTGGGCAGA
KIV2_5(-)5098bp       atcccacaatacagacgtcaaaacccataccagttattccagagagatggattgggcaga
KIV2_2(-)5096bp       atcccacaatacagacgtcaaaacccataccagttattccagagagatggattgggtagg
KIV2_4(-)5096bp       atcccacaatacagacgtcaaaacccataccagttattccagagagatggattgggcaga
KIV2-1(-)5099bp       atcccacaatacagacgtcaaaacccataccagttattccagagagatggattgggcaga
KIV2_6(-)5104bp       atcccacaatacagacgtcaaaacccataccagttattccagagagatggattgggcaga
                      ******************************************************** ** 

KIV2B_3(-)5101bp      AGGCAGAAGGAGAATACTCTGATCGTTTTTCGGCCACGTGTGTGTGTTATCTCAGTGTTT
KIV2_5(-)5098bp       aggcagaaggagaatactctgatcgtttttcggccacgtgtgtgtgttatctcagtgttt
KIV2_2(-)5096bp       aggcagaaggagaatactctgatcgtttttcggccacgtgtgtgtgttatctcagtgttt
KIV2_4(-)5096bp       aggcagaaggagaatactctgatcgtttttcggccacgtgtgtgtgttatctcagtgttt
KIV2-1(-)5099bp       aggcagaaggagaatactctgatcgtttttcggccacgtgtgtgtgttatctcagtgttt
KIV2_6(-)5104bp       aggcagaaggagaatactctgatcgtttttcggccacgtgtgtgtgttatctcagtgttt
                      ************************************************************

KIV2B_3(-)5101bp      CTAAGAAGCGTTTGCTACTTTAGATTTTTTATTTAAAAAAAAATAGTAATAATCTATTAAG
KIV2_5(-)5098bp       ctaagaagcgtttgctactttagattttttatttaaaaaaaaatagtaataatctattaag
KIV2_2(-)5096bp       ctaagaagcgtttgctactttagattttttatttaaaaaaaaatagtaataatctattaag
KIV2_4(-)5096bp       ctaagaagcgtttgctactttagattttttatttaaaaaaaaatagtaataatctattaag
KIV2-1(-)5099bp       ctaagaagcgtttgctactttagattttttatttaa-aaaaaatagtaataatctattaag
KIV2_6(-)5104bp       ctaagaagcgtttgctactttagattttttatttaaaaaaaaatagtaataatctattaag
                      ************************************ ***********************
```

```
KIV2B_3(-)5101bp      TATGAGAGATGTGCAGAGAGGATTAGTGATCGAGAGCCATTTTTGCTGGTGGCAATCATA
KIV2_5(-)5098bp       tatgagagatgtgcagagaggattagtgatcgagagccatttttgctggtggcaatcata
KIV2_2(-)5096bp       tatgagagatgtgcagagaggattagtgatcgagagccatttttgctggtggcaatcata
KIV2_4(-)5096bp       tatgagagatgtgcagagaggattagtgatcgagagccatttttgctggtggcaatcata
KIV2-1(-)5099bp       tatgagagatgtgcagagaggattagtgatcgagagccatttttgctggtggcaatcata
KIV2_6(-)5104bp       tatgagagatgtgcagagacgattagtgatcgagagccatttttgctggtggcaatcata
                      ******************* ****************************************

KIV2B_3(-)5101bp      TGGTACTTTTAATGGGAATATTAGAAAGGCACCGGTAATGACCTTGTTGCAGCACAAAGG
KIV2_5(-)5098bp       tggtacttttaatgggaatattagaaaggcaccggtaatgaccttgttgcagcacaaagg
KIV2_2(-)5096bp       tggtacttttaatgggaatattagaaaggcaccggtaatgaccttgttgcagcacaaagg
KIV2_4(-)5096bp       tggtacttttaatgggaatattagaaaggcaccggtaatgaccttgttgcagcacaaagg
KIV2-1(-)5099bp       tggtacttttaatgggaatattagaaaggcaccggtaatgaccttgttgcagcacaaagg
KIV2_6(-)5104bp       tggtacttttaatgggaatattagaaaggcaccggtaatgaccttgttgcagcacaaagg
                      ************************************************************

KIV2B_3(-)5101bp      AGAGAGTGTGGGGTGCCCCTGCATGTTGTCCCACCTCTTGTGACGTGTATCGTTTTGGAA
KIV2_5(-)5098bp       agagagtgtggggtgcccctgcatgttgtcccacctcttgtgacgtgtatcgttttggaa
KIV2_2(-)5096bp       agagagtgtggggtgcccctgcatgttgtcccacctcttgtgacgtgtatcgttttggaa
KIV2_4(-)5096bp       agagagtgtggggtgcccctgcatgttgtcccacctcttgtgacgtgtatcgttttggaa
KIV2-1(-)5099bp       agagagtgtggggtgcccctgcatgttgtcccacctcttgtgacgtgtatcgttttggaa
KIV2_6(-)5104bp       agagagtgtggggtgcccctgcatgttgtcccacctcttgtgacgtgtatcgttttggaa
                      ************************************************************

KIV2B_3(-)5101bp      TTTCCAGTGGCTTGATCATGAACTACTGCAGGAATCCAGATGCTGTGGCAGCTCCTTATT
KIV2_5(-)5098bp       tttccagtggcttgatcatgaactactgcaggaatccagatgctgtggcagctccttatt
KIV2_2(-)5096bp       tttccagtggcttgatcatgaactactgcaggaatccagatgctgtggcagctccttatt
KIV2_4(-)5096bp       tttccagtggcttgatcatgaactactgcaggaatccagatgctgtggcagctccttatt
KIV2-1(-)5099bp       tttccagtggcttgatcatgaactactgcaggaatccagatgctgtggcagctccttatt
KIV2_6(-)5104bp       tttccagtggcttgatcatgaactactgcaggaatccagatgctgtggcagctccttatt
                      ************************************************************
```

```
KIV2B_3(-)5101bp      GTTATACGAGGGATCCCGGTGTCAGGTGGGAGTACTGCAACCTGACGCAATGCTCAGACG
KIV2_5(-)5098bp       gttatacgagggatcccggtgtcaggtgggagtactgcaacctgacgcaatgctcagacg
KIV2_2(-)5096bp       gttatacgagggatcccggtgtcaggtgggagtactgcaacctgacgcaatgctcagacg
KIV2_4(-)5096bp       gttatacgagggatcccggtgtcaggtgggagtactgcaacctgacgcaatgctcagacg
KIV2-1(-)5099bp       gttatacgagggatcccggtgtcaggtgggagtactgcaacctgacgcaatgctcagacg
KIV2_6(-)5104bp       gttatacgagggatcccggtgtcaggtgggagtactgcaacctgacgcaatgctcagacg
                      ************************************************************

KIV2B_3(-)5101bp      CAGAAGGGACTGCCGTCGCGCCTCCGACTGTTACCCCGGTTCCAAGCCTAGAGGCTCCTT
KIV2_5(-)5098bp       cagaagggactgccgtcgcgcctccgactgttaccccggttccaagcctagaggctcctt
KIV2_2(-)5096bp       cagaagggactgccgtcgcgcctccgactgttaccccggttccaagcctagaggctcctt
KIV2_4(-)5096bp       cagaagggactgccgtcgcgcctccgactgttaccccggttccaagcctagaggctcctt
KIV2-1(-)5099bp       cagaagggactgccgtcgcgcctccgactgttaccccggttccaagcctagaggctcctt
KIV2_6(-)5104bp       cagaagggactgccgtcgcgcctccgactgttaccccggttccaagcctagaggctcctt
                      ************************************************************

KIV2B_3(-)5101bp      CCGAACAAGGTAAGGAGTCTGTGGCCAGACATCTACACGCTTCGATGCTGGGATGAAAAG
KIV2_5(-)5098bp       ccgaacaaggtaaggagtctgtggccagacatctacacgcttcgatgctgggatgaaaag
KIV2_2(-)5096bp       ccgaacaaggtaaggagtctgtggccagacatctacacgcttcgatgctgggatgaaaag
KIV2_4(-)5096bp       ccgaacaaggtaaggagtctgtggccagacatctacacgcttcgatgctgggatgaaaag
KIV2-1(-)5099bp       ccgaacaaggtaaggagtctgtggccagacatctacacgcttcgatgctgggatgaaaag
KIV2_6(-)5104bp       ccgaacaaggtaaggagtctgtggccagacatctacacgcttcgatgctgggatgaaaag
                      ************************************************************
```

```
KIV2B_3(-)5101bp      CCATGGAAATTCCCACTGATGCAGCCGCCTTCAATGGTAAACGGATGCTCGAGTGTTGCC
KIV2_5(-)5098bp       ccatggaaattcccactgatgcagccgccttcaatggtaaacggatgctcgagtgttgcc
KIV2_2(-)5096bp       ccatggaaattcccactgatgcagccgccttcaatggtaaacggatgctcgagtgttgcc
KIV2_4(-)5096bp       ccatggaaattcccactgatgcagccgccttcaatggtaaacggatgctcgagtgttgcc
KIV2-1(-)5099bp       ccatggaaattcccactgatgcagccgccttcaatggtaaacggatgctcgagtgttgcc
KIV2_6(-)5104bp       ccatggaaattcccactgatgcagccgccttcaatggtaaacggatgctcgagtgttgcc
                      ************************************************************

KIV2B_3(-)5101bp      TGAGTTCTACCATGTAGGAGGAAGCCTCCGTGCACTCTCTGGGGGAGCCAGCGGAGTGAT
KIV2_5(-)5098bp       tgagttctaccatgtaggaggaagcctccgtgcactctctgggggagccagcggagtgat
KIV2_2(-)5096bp       ggagttctgccatgtttggggggaagcctccgtgtactctctgggggagccagcggagtgat
KIV2_4(-)5096bp       tgagttctaccatgtaggaggaagcctccgtgcactctctgggggagccagcggagtgat
KIV2-1(-)5099bp       tgagttctaccatgtaggaggaagcctccgtgcactctctgggggagccagcggagtgat
KIV2_6(-)5104bp       tgagttctaccatgtaggaggaagcctccgtgcactctctgggggagccagcggagtgat
                       ******* ****** ** ************* ***************************

KIV2B_3(-)5101bp      TTCTGGTG
KIV2_5(-)5098bp       ttctggtg
KIV2_2(-)5096bp       ttctggtg
KIV2_4(-)5096bp       ttctggtg
KIV2-1(-)5099bp       ttctggtg
KIV2_6(-)5104bp       ttctggtg
                      ********
```

5 References

1. Coassin, S., G. Erhart, H. Weissensteiner, M. Eca Guimarães de Araújo, C. Lamina, S. Schönherr, L. Forer, M. Haun, J. L. Losso, A. Köttgen, K. Schmidt, G. Utermann, A. Peters, C. Gieger, K. Strauch, A. Finkenstedt, R. Bale, H. Zoller, B. Paulweber, K.-U. Eckardt, A. Hüttenhofer, L. A. Huber, and F. Kronenberg. 2017. A novel but frequent variant in LPA KIV-2 is associated with a pronounced Lp(a) and cardiovascular risk reduction. *Eur Heart J*. **38**: 1823–1831.
2. Noureen, A., F. Fresser, G. Utermann, and K. Schmidt. 2015. Sequence Variation within the KIV-2 Copy Number Polymorphism of the Human LPA Gene in African, Asian, and European Populations. *PLoS One*. **10**: e0121582.
3. Parson, W., H. G. Kraft, H. Niederstätter, A. W. Lingenhel, S. Köchl, F. Fresser, and G. Utermann. 2004. A common nonsense mutation in the repetitive Kringle IV-2 domain of human apolipoprotein(a) results in a truncated protein and low plasma Lp(a). *Hum Mutat*. **24**: 474–80.
4. McLean, J. W., J. E. Tomlinson, W. J. Kuang, D. L. Eaton, E. Y. Chen, G. M. Fless, A. M. Scanu, and R. M. Lawn. 1987. cDNA sequence of human apolipoprotein(a) is homologous to plasminogen. *Nature*. **330**: 132–7.
5. Rosby, O., P. Aleström, and K. Berg. 2000. Sequence conservation in kringle IV-type 2 repeats of the LPA gene. *Atherosclerosis*. **148**: 353–64.
6. Sievers, F., A. Wilm, D. Dineen, T. J. Gibson, K. Karplus, W. Li, R. Lopez, H. McWilliam, M. Remmert, J. Soding, J. D. Thompson, and D. G. Higgins. 2014. Fast, scalable generation of high-quality protein multiple sequence alignments using Clustal Omega. *Mol Syst Biol*. **7**: 539–539.
7. Weissensteiner, H., L. Forer, C. Fuchsberger, B. Schöpf, A. Kloss-Brandstätter, G. Specht, F. Kronenberg, and S. Schönherr. 2016. mtDNA-Server: next-generation sequencing data analysis of human mitochondrial DNA in the cloud. *Nucleic Acids Res*. **44**: W64-9.
8. Mancini, F. P., V. Mooser, R. Guerra, and H. H. Hobbs. 1995. Sequence microheterogeneity in apolipoprotein(a) gene repeats and the relationship to plasma Lp(a) levels. *Hum Mol Genet*. **4**: 1535–42.
9. Byrne, C. D., K. Schwartz, K. Meer, J. F. Cheng, and R. M. Lawn. 1994. The human apolipoprotein(a)/plasminogen gene cluster contains a novel homologue transcribed in liver. *Arter Thromb*. **14**: 534–41.
10. Larkin, M. A., G. Blackshields, N. P. Brown, R. Chenna, P. A. McGettigan, H. McWilliam, F. Valentin, I. M. Wallace, A. Wilm, R. Lopez, J. D. Thompson, T. J. Gibson, and D. G. Higgins. 2007. Clustal W and Clustal X version 2.0. *Bioinformatics*. **23**: 2947–2948.
11. den Dunnen, J. T., R. Dalgleish, D. R. Maglott, R. K. Hart, M. S. Greenblatt, J. Mcgowan-Jordan, A. F. Roux, T. Smith, S. E. Antonarakis, and P. E. M. Taschner. 2016. HGVS Recommendations for the Description of Sequence Variants: 2016 Update. *Hum Mutat*.
12. Ratan, A., T. L. Olson, T. P. Loughran, and W. Miller. 2015. Identification of indels in next-generation sequencing data. *BMC Bioinformatics*. **16**: 42.
13. Heid, I. M., S. A. Wagner, H. Gohlke, B. Iglseder, J. C. Mueller, P. Cip, G. Ladurner, R. Reiter, A. Stadlmayr, V. Mackevics, T. Illig, F. Kronenberg, and B. Paulweber. 2006. Genetic architecture of the APM1 gene and its influence on adiponectin plasma levels and parameters of the metabolic syndrome in 1,727 healthy Caucasians. *Diabetes*. **55**: 375–384.
14. Eckardt, K.-U., B. Bärthlein, S. Baid-Agrawal, A. Beck, M. Busch, F. Eitner, A. B. Ekici, J. Floege, O. Gefeller, H. Haller, R. Hilge, K. F. Hilgers, J. T. Kielstein, V. Krane, A. Köttgen, F. Kronenberg, P. Oefner, H.-U. Prokosch, A. Reis, M. Schmid, E. Schaeffner, U. T. Schultheiss, S. A. Seuchter, T. Sitter, C. Sommerer, G. Walz, C. Wanner, G. Wolf, M. Zeier, and S. Titze. 2012. The German Chronic Kidney Disease (GCKD) study: design and methods. *Nephrol Dial Transplant*. **27**: 1454–60.
15. Kronenberg, F., E. Kuen, E. Ritz, R. Junker, P. Konig, G. Kraatz, K. Lhotta, J. F. Mann, G. A. Muller, U. Neyer, W. Riegel, P. Reigler, V. Schwenger, A. Von Eckardstein, P. König, G. Kraatz, K. Lhotta, J. F. Mann, G. A. Müller, U. Neyer, W. Riegel, P. Reigler, V. Schwenger, A. Von Eckardstein, P. Konig, G. Kraatz, K. Lhotta, J. F. Mann, G. A. Muller, U. Neyer, W. Riegel, P. Reigler, V. Schwenger, A. Von Eckardstein, P. König, G. Kraatz, K. Lhotta, J. F. Mann, G. A.

Müller, U. Neyer, W. Riegel, P. Reigler, V. Schwenger, A. Von Eckardstein, P. Konig, G. Kraatz, K. Lhotta, J. F. Mann, G. A. Muller, U. Neyer, et al. 2000. Lipoprotein(a) serum concentrations and apolipoprotein(a) phenotypes in mild and moderate renal failure. *J Am Soc Nephrol*. **11**: 105–15.

16. Erhart, G., C. Lamina, T. Lehtimäki, P. Marques-Vidal, M. Kähönen, P. Vollenweider, O. T. Raitakari, G. Waeber, B. Thorand, K. Strauch, C. Gieger, T. Meitinger, A. Peters, F. Kronenberg, and S. Coassin. 2018. Genetic Factors Explain a Major Fraction of the 50% Lower Lipoprotein(a) Concentrations in Finns. *Arterioscler Thromb Vasc Biol*. **38**: 1230–1241.
17. Lanktree, M. B., C. Rajakumar, J. H. Brunt, M. L. Koschinsky, P. W. Connelly, and R. A. Hegele. 2009. Determination of lipoprotein(a) kringle repeat number from genomic DNA: copy number variation genotyping using qPCR. *J Lipid Res*. **50**: 768–772.
18. Weaver, S., S. Dube, A. Mir, J. Qin, G. Sun, R. Ramakrishnan, R. C. Jones, and K. J. Livak. 2010. Taking qPCR to a higher level: Analysis of CNV reveals the power of high throughput qPCR to enhance quantitative resolution. *Methods*. **50**: 271–276.
19. Li, H., B. Handsaker, A. Wysoker, T. Fennell, J. Ruan, N. Homer, G. Marth, G. R. Abecasis, and R. Durbin. 2009. The Sequence Alignment/Map format and SAMtools. *Bioinformatics*. **25**: 2078–2079.
20. Quinlan, A. R., and I. M. Hall. 2010. BEDTools: A flexible suite of utilities for comparing genomic features. *Bioinformatics*. **26**: 841–842.
